# Epidemiological scenarios for human rabies exposure notified in Colombia during ten years: A challenge to implement surveillance actions with a differential approach on vulnerable populations

**DOI:** 10.1371/journal.pone.0213120

**Published:** 2019-12-27

**Authors:** Marcela Rocío Arias Caicedo, Diego de Arruda Xavier, Catalina Alejandra Arias Caicedo, Etiene Andrade, Isis Abel

**Affiliations:** 1 Laboratório de Epidemiologia e Geoprocessamento, Programa de Pós-Graduação em Saúde Animal na Amazônia, Universidade Federal do Para, Castanhal, Para, Brasil; 2 Ciências da Terra e Ecologia, Programa de Capacitação Institucional, Museu Paraense Emílio Goeldi, Campus de Pesquisa, Belém, Para, Brasil; 3 Dirección de Vigilancia y Análisis de Riesgo en Salud Pública, Instituto Nacional de Salud de Colombia, Bogotá D.C., Colombia; 4 Laboratório de Epidemiologia e Geoprocessamento, Programa de Pós-Graduação em Estudos Antrópicos da Amazônia, Universidade Federal do Pará, Castanhal, Pará, Brasil; Faculty of Science, Ain Shams University (ASU), EGYPT

## Abstract

Based on notified cases of human rabies exposure and human deaths by rabies to Colombia public health surveillance system between 2007 and 2016, we conducted a spatiotemporal analysis to identify epidemiological scenarios of high human rabies exposure due to dogs, cats, bats, or farm animals (n = 666,411 cases). The incidence rate of human rabies exposures was analyzed by using geographical information system (spatiotemporal distribution and Cluster and Outlier Analysis (Anselin Local Moran’s I)) data for all Colombian cities. The incidence rate of human rabies exposures due to dogs and cats showed an increasing trend, while aggression due bats and farm animals fluctuated throughout the analyzed period. Human deaths by rabies transmitted by cat and bat occurred in the Andean and Orinoquia regions, which had urban and rural scenarios. The urban scenario showed the highest exposure to human rabies due to cats and dogs in cities characterized with high human population density and greater economic development. In contrary, the highest human rabies exposure in the rural scenario was observed due to contact of mucosa or injured skin with the infected saliva of farm animals with the rabies virus, principally among workers in the agroforestry area. The inequality scenario showed some outlier cities with high human rabies exposure due to farm animals principally in the Pacific region (characterized by the highest poverty rates in Colombia), being Afro-descendant and indigenous population the most exposed. The highest exposure due to bats bite was observed among indigenous people residing in cities of the Amazon region as a dispersed population (Amazonian scenario). None of the high exposure scenarios were related to human deaths by rabies due to dogs aggression. The identified scenarios can help develop better surveillance systems with a differential approach to the vulnerable population and strengthening them in areas with rabies viral circulation.

## Introduction

Rabies is a globally notorious infectious disease which causes viral encephalitis with high mortality in humans. Rabies Virus (RABV) belongs to the order *Mononegavirales*, family *Rhabdoviridae*, genus *Lyssavirus*, and genotype 1 [1]. It is believed that RABV appeared more than 4000 years ago with the bat as a reservoir species and adapted to different geographical areas and new hosts in its evolutionary process [2]. RABV can be classified by its antigenic composition so that the primary reservoir is identified [3], the main reservoirs where the antigenic variants (V) were found are the dog, the mongoose, the hematophagous bat, the *Tadarida brasillensis* bat, the *Lasiurus cinereus* bat, the Arizona fox, the skunk, the California skunk, the gray wolf and the polar fox [4]. It is transmitted by saliva contact contaminated with the rabies virus through a bite, scratch, or lick from bat, dog, cat, and other mammals to humans [5]. Few cases of transmission by sick mothers in the periparto [6], due to organ transplants [7,8] and by airborne particles[9] are also known. Its transmission by dog bites is the most heard news and generates the greatest interest in the human population. Statistical information also support this type of transmission as the main global risk, with 95% of human deaths by rabies (HDR) caused by dog bites, mainly in the African and Asian continents [10, 11].The remaining 5% of HDR are caused by wild animal’s bites and are of high concern for public health, especially in the Americas, where wild animals are considered as the main transmitters of the disease [12]. HDR transmitted by bat bites have recently emerged in the Americas, mainly occurring as outbreaks in highly vulnerable human populations [13] and in areas where the following hematophagous bat species that are only found in America exist: *Desmodus rotundus*, *Dyphilla ecaudata*, *and Diaemus young* [14].

Historically; Colombia, like other countries of Latin America, presented HDR caused by dogs bites, although these HDR considerably decreased from 1981 to 2004 [15]. This change was due to intense institutional and governmental work in the country and in the Americas to eliminate rabies transmitted by dogs to humans [16]. After 1995, the HDR presents in Colombia were caused by variants V1 and V2 related to dog as a reservoir and principal transmitter followed by antigenic variants V3, V5 and V atypical related to hematophagous bat as a reservoir and transmitter, antigenic variant V4 related to the bat *Tadarida brasillensis* as a reservoir and antigenic variant V8 related to the skunk *Mephitis mephitis* e *Spilogale putorius* as a reservoir and the cat as transmitter [17].

To prevent HDR, the Colombian government monitors the aggression of animals to humans from the beginning of the clinical care of a patient injured through the public health surveillance system called SIVIGILA [18]. The system involves hospitals and health units as entities responsible for the care and generation of reports, which makes it possible to obtain information for surveillance, follow up, control and prevention action conducted by the local health secretariats. The National Health Institute (INS, for its acronym in Spanish) analyzes the national data, generating the pertinent investigative, preventive and surveillance actions and the Ministry of Health and Social Protection (MSPS, for its acronym in Spanish) together with the INS generate the necessary projects and policies to prevent human rabies in Colombia [18].

The World Health Organization (WHO) has worked hard with Colombia government to reduce social inequities in health care, promoting strategies and policies adapted to each national context [19]. Colombia has, in recent years, created policies in favor of social equity in health and incorporated into its development plans the differential approach, which has been defined by the Colombian Government as “a way of analyzing, acting, assessing and guaranteeing the development of a population, based on its differential characteristics from a perspective of equity and diversity.” These are expected to be reflected in future health analyses based on its effective implementation in each territorial health entity of the country [20]. An example of that is the Model of surveillance strategy, prevention and control of wild rabies in communities of high risk created in 2012 to serve populations located in areas of difficult access executed between 2012 and 2014 in the departments of Vaupés, Cauca, Nariño and Chocó [21] with the principal objective of apply anti-rabies pre-exposure scheme in this population [22].Even so, the focus of control and prevention; derived from public politics, are generalized for the entire country, impacting mainly areas of population concentration and not integrating the complexity of the Colombian territory [23]. To an effective surveillance it is necessary to analyze SIVIGILA data in deep and a spatial approach should be important to improve surveillance measures.

Spatial analysis helps understand the behavior of diseases from a geographical perspective to identify information on significant clusters and associated factors. These analytical tools can be used to monitor epidemiological indicators over time, to identify risk factors and clusters of high endemicity and to indicate where additional resources should be targeted [24]. Studies on the distribution and spatial analysis of human rabies infection or human rabies exposure (HRE) in Colombia are few; they are frequent on the topics of livestock rabies and in molecular epidemiology [25, 26].

Based on the reported cases of HRE and HDR via SIVIGILA between 2007 and 2016, this study aims to realize a spatiotemporal analysis to identify epidemiological trends and areas with high incidence of HRE due to dogs, cats, farm animals or bats to determine the epidemiological scenarios of high incidence of HRE characterized by the sociodemographic and aggression information included in the notification. This analysis is oriented in order to improve surveillance and prevention programs, looking to focus in design operational strategies according to geographical and sociodemographic conditions where differential approach and strengthening social equity in health becoming relevant.

## Materials and methods

### Study area

**Fig 1:**
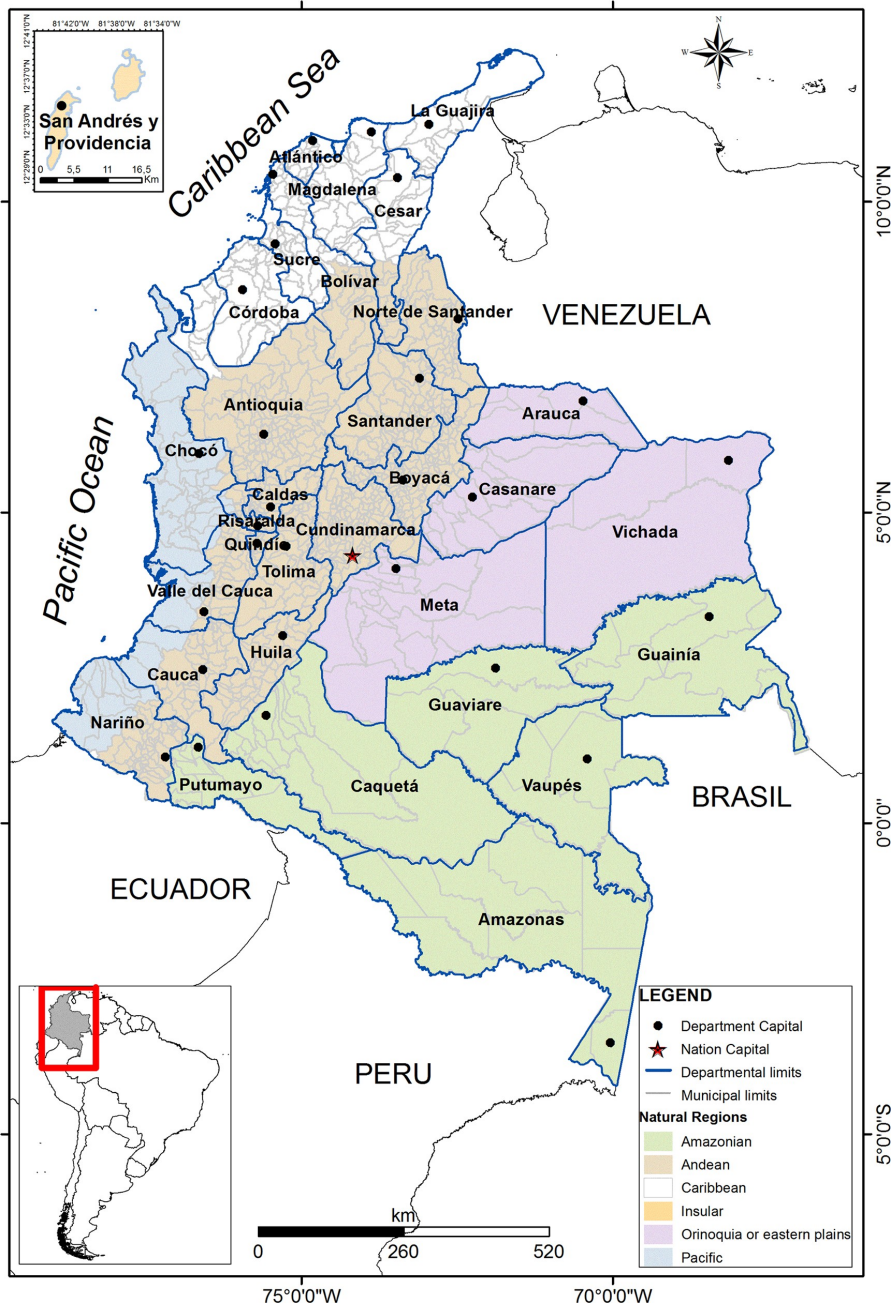
Political division and natural regions of Colombia. Amazonian region (green), Orinoquia region (violet), Andean region (brown), Pacific region (blue), Caribbean region (white), and Insular region (orange).

Colombia is located at the northwest of the South American continent. According to National Administrative Department of Statistics (DANE, for its acronym in Spanish) Colombia in 2017, presents a population of 49,291,609 and an area of 1,143,407 km2. It is divided into 32 departments, and subdivided into 1,122 cities (1,102 municipalities and 20 non-municipalized areas called corregimientos) [27]. Cities are organized into six regions divided by topographical variety, relief, vegetation, weather, biota and geology [28]: Amazonian, Andean, Orinoquia or Eastern plains, Caribbean, Pacific, and Insular regions [28] (Fig 1).

### Regional epidemiological context in Colombia

The last HDR transmitted by dogs in Colombia (four cases) with V1 identified, occurred between 2006 and 2007 in Magdalena Department [29], inserted in Caribbean region; where the most human deaths by rabies transmitted by dogs and dog’s rabies have been reported in Colombia history [30]. The Caribbean region is located on the Caribbean coast formed by cities with a high tourist influx and an economy based on livestock activity and extraction of coal, banana and nickel [28]. It showed the highest Adjusted Incidence of Multidimensional Poverty Rates, AIMPR, (a low AIMPR indicates higher per capita income, better quality of life conditions and better access to health) in Colombia during 2010 to 2014 (20.7, 19.0, 18.4, 16.5 and 14.9 respectively) according to DANE [31].

The first reported of HDR due bats bites (3 cases) in Colombia were in Chocó Department, located in Pacific region in 1996 [32]. Another HDR due bats were reported in Chocó between 2005 and 2006 with 14 cases in indigenous population and 3 in Afro-descendant communities with V3 detected. The cases were observed in forest area with difficult access, with problems of public order and low access to health [33]. The Pacific region is located in Chocó forest [34] in Pacific coast characterized for being in its majority Afro-descendant and indigenous population who survives from the mining and forestry exploitation [28,35]. During 2015 and 2016 showed the highest AIMPR (14.6, and 14.0 respectively) according to DANE [31].

Until the year 1998; the Amazonian and Andean region did not present HDR. The first case of human rabies in Andean region was in 2003 in Quipile city, caused by a cat and the V8 was confirmed without more cases until 2006 [32], this region is located at center-east of the country in the Andes Mountain, separating Chocó Forest from Amazon forest [34].Its principal characteristic is that concentrates the largest economic activities and the largest population in the country [28]. In Amazon region the population is mainly indigenous who subsists of family farming and forestry exploitation in the Amazon forest [28,35]. It has only been reported two cases of human rabies transmitted by dog with V1 identified in Putumayo city (Colombia-Ecuador border) in 1999 and 2000 [32].

Orinoquia and Insular region did not present HDR before the period of analysis of the present study [32]. The Orinoquia Region is characterized by great plains where livestock and African palm cultivation are established as the principal economic activities [28]. According to DANE, presented from 2010 to 2016 the lowest AIMPR with 12.1, 12.1, 11.7, 10.2, 7.3, 7.0 and 6.7 respectively [31]. The insular region is located in the islands present in Caribbean maritime waters; its main economic activity is tourism and fishing [28].

### Data collection and analysis

The SIVIGILA for Rabies in Colombia is strengthened since 1983; with the objective of eliminating the human rabies transmitted by dogs-V1 [36]. In 2010 has adhered to the objective of controlling human rabies of wild origin [32].With the recommendations given by Pan American Health Organization (PAHO) and Rabies experts; the Protocol for integrated surveillance of human rabies at the national level, is established and updated periodically [37]. The protocol establishes the HRE cases care as a medical emergency with immediate notification of HRE and confirmed cases of HDR. An HRE is defined as: "the probability of penetration and replication of the rabies virus in the body of a person who has suffered an injury due to the aggression of an animal potentially transmitting rabies (any wound or injury caused by bites, scratches, whatever their number, extent or depth, in any part of a person's body), or contact of injured skin or mucosa with saliva or tissue of an animal or human infected or presumably infected with the rabies virus, either accidentally or through improper biosecurity practices in zoonosis centers, caves with bats, diagnostic laboratories, research or preparation of rabies vaccine, among others" [38]. The report is made through SIVIGILA software, where the mandatory and immediate notification of a HRE or HDR forms is loaded by the patient's health care entity [20]. The notification forms consist of four sections: 1-Basic patient data, 2-Exposure and immunization history, 3-Clinical care data and 4-Follow-up and research data [18].

Data of reported cases of HRE since 2007 to 2016 were obtained from SIVIGILA and exported to IBM SPSS software version 20.0 for statistical analysis. Data without details about the aggressor species, aggressions by animals not analyzed in the present study and people who were exposed to RABV in a different country were excluded (n = 12,232 cases; 1.8%), resulting in 666,411 valid cases (98.2%). For the analyses were used the sociodemographic variables originated in the section of basic patient data of the notification form: age, sex, occupation, ethnicity, area of occurrence of the case and city where aggression or contact occurred. The variables used from other sections were aggressor species, aggression type, patient’s final condition and variant detected. The cases of HDR and variant detected were confirmed from final report of HDR in Colombia in 2016 [15].

For descriptive statistical analysis, age variable was categorized in ten-year intervals, and occupation variable was categorized according to the International Standard Industrial Classification of All Economic Activities (ISIC) adapted to Colombia [39]. Gender, area and ethnicity variables remained classified according to the Rabies surveillance protocol [18]. For aggressor species variable, the aggression data due to dogs, cats, bats, bovines, ovine, porcines and equines were used. Bovine, ovine, porcine and equine species were analyzed in a single group called farm animals.

### Spatiotemporal analysis

The population distribution data by year/municipalities [40] and Colombia shapefiles were obtained from DANE [27] and used as a basis for spatial analysis. The process was conducted to see first the incidence distribution and then obtain the high incidence with statistical significance.

The incidence means of HRE due to dogs, cats, bats, and farm animals from 2007 to 2016 and incidence rate of HRE by year/100,000 habitants were estimated and included in the spatiotemporal analysis to see its geographical distribution. The incidence rates were subdivided into levels according to quartiles (Q)—denominated: 0: No incidence; Q1: Low incidence; Q2: Moderate incidence; Q3: High Incidence and Q4: Very high incidence [41]. Also were included HDR data to show its distribution by time and by cities. Maps were created in ArcGIS 10.3 software, and temporal graphics were developed in Microsoft Excel.

Regarding spatial statistical analysis, Moran’s global index was applied to check spatial pattern of the HRE incidences by aggressor species type (if it was grouped, scatter or random). Only HRE incidences that showed spatial pattern grouped (z< -1.96 o > + 1.96 and P <0.05) were selected to spatial autocorrelation by Cluster and Outlier Analysis (Anselin Local Moran’s I) for determining hot spots, cold spots and statistically significant spatial outliers lower case p for p-value (P <0.05) [42,43]. Both analyses were conducted in ArcGIS 10.3 software. Once spatial statistics analysis results were obtained, we proceeded to analyze the High-High cluster (HH) and High-Low outlier (HL) by aggressor species. Descriptive analysis was performed to describe the sociodemographic variables and aggression information according to the data involved in cities located in HH and HL using IBM SPSS software version 20.0.

To calculate the population density, the population means by each city from 2007 to 2016 (obtaining from DANE) [40] were divided with the size of the area in ArcGIS software of each HH and HL by animal aggressor type. Epidemiological scenarios were determined as a result of high aggression analysis in a geographic area (scenario) with similar trend and sociodemographic characteristics included in SIVIGILA during the study period (epidemiological characteristics).

### Ethical considerations

To do the research, the National Institute of Health of Colombia gave permission to use the data, approved the study and transferred the information for use it in the period of 2007 to 2016. Data from SIVIGILA database were obtained in a fully anonymized and de-identified manner.

## Results

### Descriptive statistics

Between 2007 and 2016 the HRE due to dogs was the most reported, with 582,539/666,304 cases (87.4%) (Table 1). The age group that was most frequently exposed was 0–9 years old (25.8%; 172,277/666,304), while for HRE due to farm animals, the age group of 30–39 years had the highest proportion (17.5%; 1,303/7,442). Although most HRE cases occur among men, the women were more exposed to cat, constituting 61% of all reported cases of HRE due to cats (44,811/73,281). The occupation most reported was student (35.5%; 236,372/666,304), and bite was the more frequent aggression type (89.6%, 601,178/666,304). Within the ethnic population, the Afro-descendant population was the most affected (3.9%, 26,344/666,411), principally by HRE due dogs; however, it represented less than 5% of the total population exposed to RABV (3.5%; 23,180/666,304).

**Table 1:** Characteristics of population exposed to rabies according to type of aggressor species in Colombia 2007–2016.

VARIABLES	AGGRESSOR SPECIES			
	DOG	CAT	BAT	FARM ANIMALS
**AGE N = 666,304**	n = 582,539 (%)	n = 73,272 (%)	n = 3,051 (%)	n = 7,442 (%)
0–9 year	157,018 (27.0)	14,670 (20.0)	589 (19.3)	819 (11.0)
10–19 years	126,489 (21.7)	11,709 (16.0)	576 (18.9)	1,254 (16.9)
20–29 years	77,776 (13.4)	10,778 (14.7)	559 (18.3)	1,287 (17.3)
30–39 years	58,487 (10.0)	8,156 (11.1)	366 (12.0)	1,303 (17.5)
40–49 years	51,574 (8.9)	8,371 (11.4)	327 (10.7)	1171 (15.7)
50–59 years	47,946 (8.2)	8,025 (11.0)	311 (10.2)	838 (11.3)
60–69 years	33,136 (5.7)	5,525 (7.5)	167 (5.5)	517 (6.9)
70–79 years	21,133 (3.6)	3,822 (5.2)	112 (3.7)	184 (2.5)
80 years and over	8,980 (1.5)	2,216 (3.0)	44 (1.4)	69 (0.9)
**GENDER N = 666411**	n = 582,636%	n = 73,281%	n = 3,051%	n = 7,443%
Female	253,919 (43.6)	44,811 (61.1)	1,267 (41.5)	1,949 (26.2)
Male	328,717 (56.4)	28,470 (38.9)	1,784 (58.5)	5,494 (73.8)
**AGRESSION TYPE N = 666411**				(0.0)
Bite	536,277 (92.0)	58,171 (79.4)	2,811 (92.1)	3,919 (52.7)
Scratch	44,354 (7.6)	14,857 (20.3)	188 (6.2)	224 (3.0)
Contact of mucosa or skin injured with saliva, nervous tissue, biological material or secretions infected with rabies virus	1,756 (0.3)	1,960 (0.2)	38 (1.2)	3,055 (41.0)
Other	249 (0.0)	57 (0.1)	14 (0.0)	245 (3.3)
**OCUPATION N = 666411**				
Student	210,340 (36.1)	22,898 (31.2)	959 (31.4)	2,175 (29.2)
Homemaker	84,658 (14.5)	13,954 (19.0)	418 (13.7)	1,025 (13.8)
Underage	55,839 (9.6)	6,290 (8.6)	303 (9.9)	640 (8.6)
Professionals, technicians and others from the agroforestry and livestock area	26,511 (4.6)	2,574 (3.5)	144 (4.7)	1,171 (15.7)
Professionals, technicians and others in organization, administration, law, financial analysis and related	16,692 (2.9)	2,647 (3.6)	91 (3.0)	175 (2.4)
Professionals, technicians and workers of the biological sciences, medicine and health	13,975 (2.4)	2,275 (3.1)	102 (3.3)	207 (2.8)
Others	17,4621 (30.0)	22,643 (30.9)	1,034 (33.9)	2,050 (27.5)
**ETHNICITY N = 666411**				
Indigenous	10,409 (1.8)	748 (1.0)	460 (15.1)	388 (5.2)
Afrodescendant	23,180 (4.0)	2,144 (2.9)	300 (9.8)	720 (9.7)
Others	3,585 (0.6)	418 (0.6)	21 (0.7)	68 (0.9)
Population without ethnic group	545,462 (93.6)	69,971 (95.5)	2,270 (74.4)	6,267 (84.2)

### Temporal analyses

**Fig 2:**
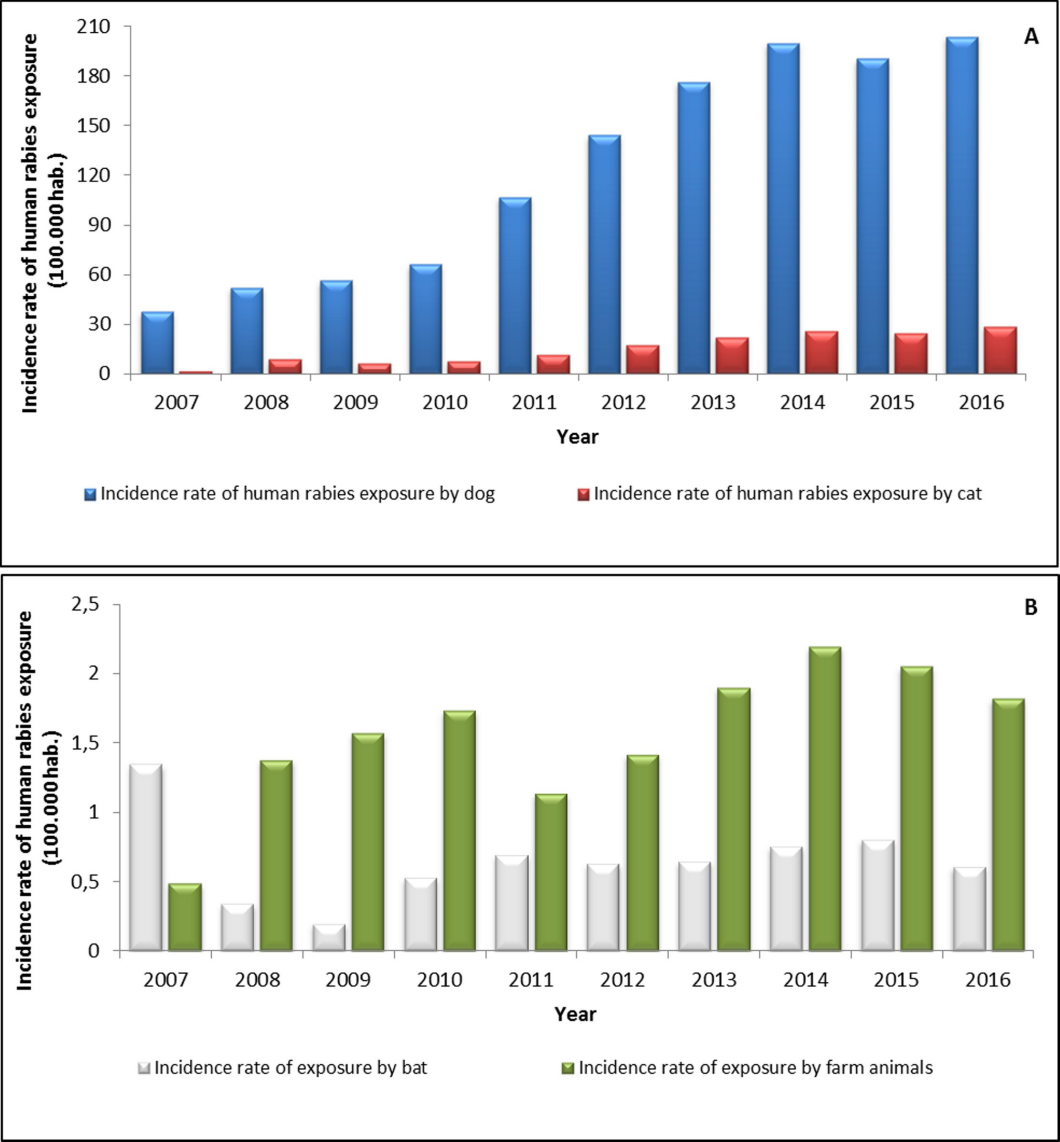
Temporal distribution of incidence rates of HRE due to dogs, cats, bats, and farm animals per 100.000 habitants in Colombia, 2007–2016. **(**A) Incidence rates and cases of HRE due to cats and dogs. (B) Incidence rates and cases of HRE due to farm animals and bats.

The HRE due to dogs and cats increased from 2007 to 2016 with the highest incidence presented in 2016 by HRE due to dogs (203.81 x 100,000 habitants) (Fig 2A). The incidence rate of HRE due to bats and farm animals fluctuated throughout the analyzed period, with peaks occurring for HRE due to bats in 2007 (1.33/100,000 habitants), 2011 (0.68/100,000 habitants), and 2015 (0.79/100,000 habitants), and for HRE due to farm animals in 2010 (1.73/100,000 habitants) and 2014 (2.19/100,000 habitants) (Fig 2B).

**Fig 3:**
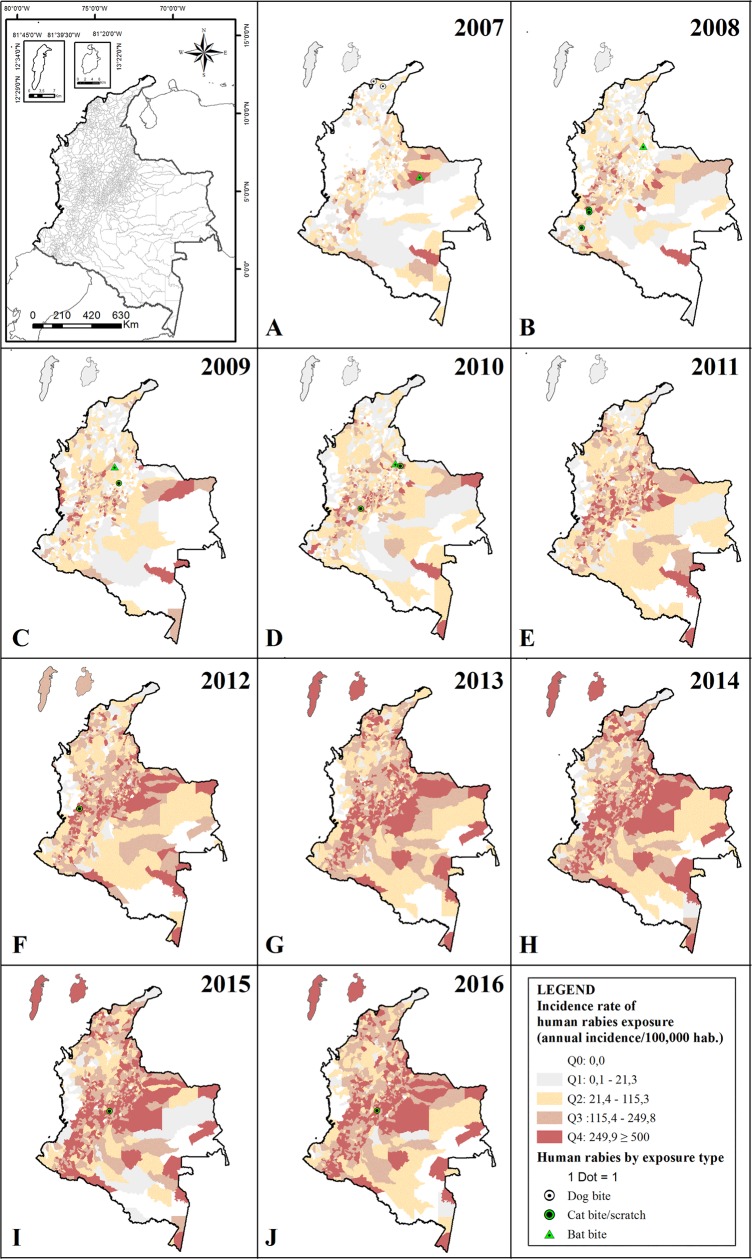
Spatiotemporal distribution of incidence rates of HRE and HDR in Colombia (2007–2016). Incidence rates of HDE and cases of HDR by animal aggressors in (A) 2007, (B) 2008, (C) 2009, (D) 2010, (E) 2012, (G) 2013, (H) 2014, (I) 2015, (J) 2016. The colored dot of HDR by aggressor species represents the variant involved: White for V1 variant of dog and Green for variants of bat (V3, V4, and atypical).

The overall incidence rates of HRE showed an increase in all the cities in the period analyzed, the HRE incidence rates increased from 40.9/100,000 habitants in 2007 to 234.9/100,000 habitants in 2016 (Fig 3). The lowest incidence rate was observed in most of the cities of the Chocó department during every studied year (83%; 26/31 cities between 0 and 21.3/100,000 habitants). In fact, some cities of Amazonian region in the Amazonas (9%, 1/11) and Guainia departments (55%; 5/9), and in Caribbean region in Chocó department (6.4%; 2/31) registered absence of incidence during all the analyzed periods (S1 Table).

The HDR transmitted by dogs only occurred in 2007, when two cases were notified at Caribbean region in Magdalena city with the V1 involved (Fig 3A). Four cases of HDR transmitted by bats occurred in two departments located in northeastern of Colombia in Orinoquia and Andean region (Casanare and Santander department respectively) with the V3 involved, in the cities San Luis de Palenque (2007), Floridablanca (2008), Barrancabermeja (2009) and Piedecuesta (2010) (Fig 3A, 3B, 3C and 3D). Ten cases of HDR transmitted by cat were recorded in six departments located in the Andean region, with the V3, V4 and V atypical involved. The V3 was identified in four HDR in Cauca and Santander departments, two in Quilichao city (2008), one HDR in Bolivar city (2008) and one in Enciso city (2010). The V4 was identified in three HDR in Boyacá and Valle del Cauca departments; one in Moniquira city (2009) and two in Roldanillo city (2012), finally the V atypical was identified in three HDR in Tolima and Cundinamarca departments; one in San Luis city (2009), one in Mesitas del Colegio city (2015) and the last in Girardot city (2016). (Fig 3B, 3C, 3D, 3F, 3I and 3J).

### Spatial analyses

**Fig 4:**
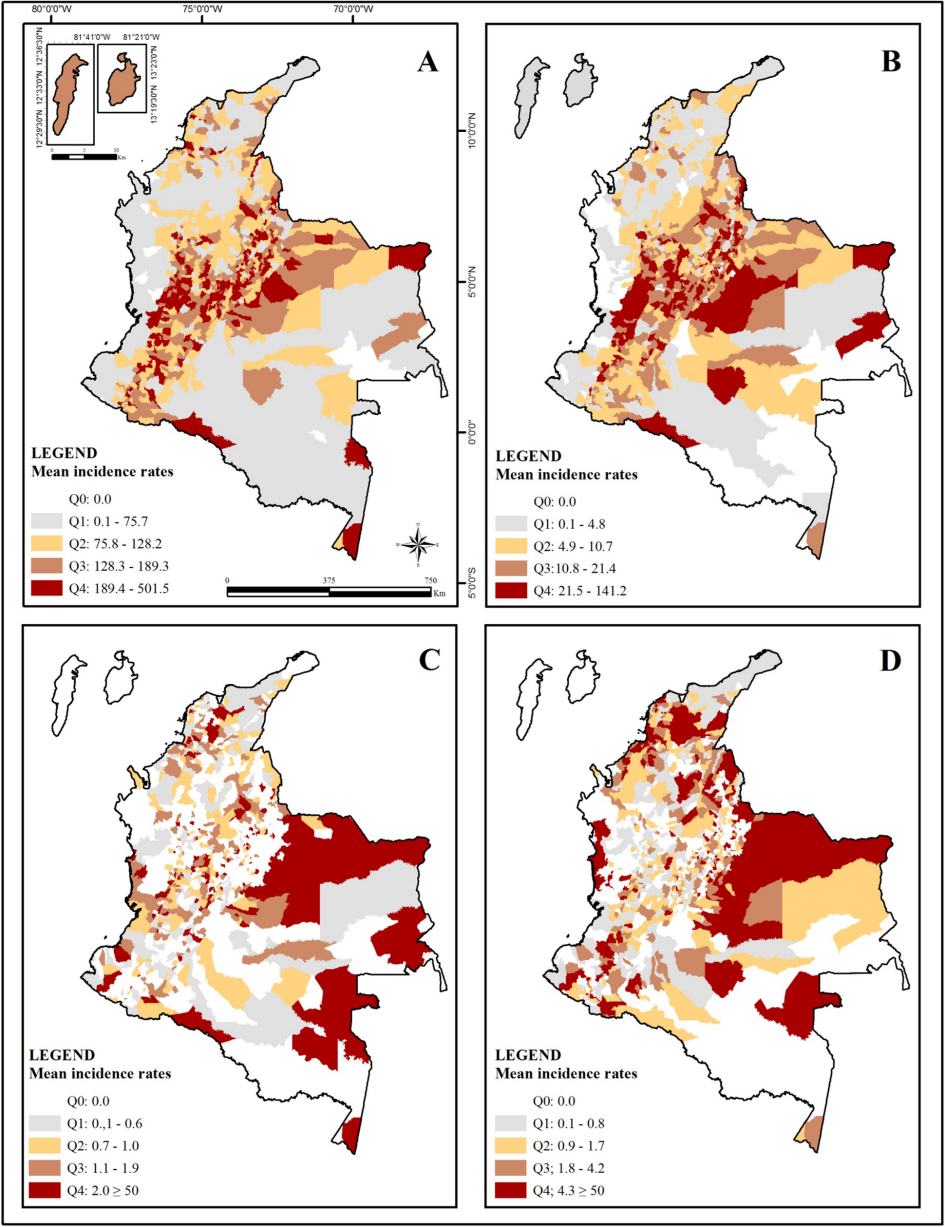
Spatial distribution of the mean incidence of HRE by aggressor species from 2007 to 2016 in all 1122 Colombian cities. (A) Mean incidence rates of HRE due to dogs (B) Mean incidence rates of HRE due to cats (C) Mean incidence rates of HRE due to bats. For this map section, Q4 presents 3 cities with incidence between 50 and 1,100/100,000 habitants. (D) Mean incidence rates of HRE due to farm animals. For this map section, Q4 presents 10 cities with incidence between 50 and 142.8/100,000 habitants. Incidence levels according to quartiles (Q): Q0—No incidence; Q1—Low incidence; Q2—Moderate incidence; Q3—High incidence and Q4—Very high incidence.

The geographic distribution of incidences rates of HRE due to dogs and cats showed a changing concentration from moderate to very high in municipalities located in the Andean Region, north of the Orinoquia region, and some municipalities of the Amazon and Caribbean regions (Fig 4A and 4B). Low incidences were present in some cities located in the Pacific, Amazon, and Caribbean regions. Very high, high, moderate, and low incidence ranges of HRE due to bats and farm animals were observed in all the regions (Fig 4C and 4D). The highest incidence rate of HRE among all animals’ species was observed in exposure due to bats in the Taraira municipality of Vaupés Department in Amazon region (1,100.5/100,000 habitants) (S2 Table)

**Fig 5:**
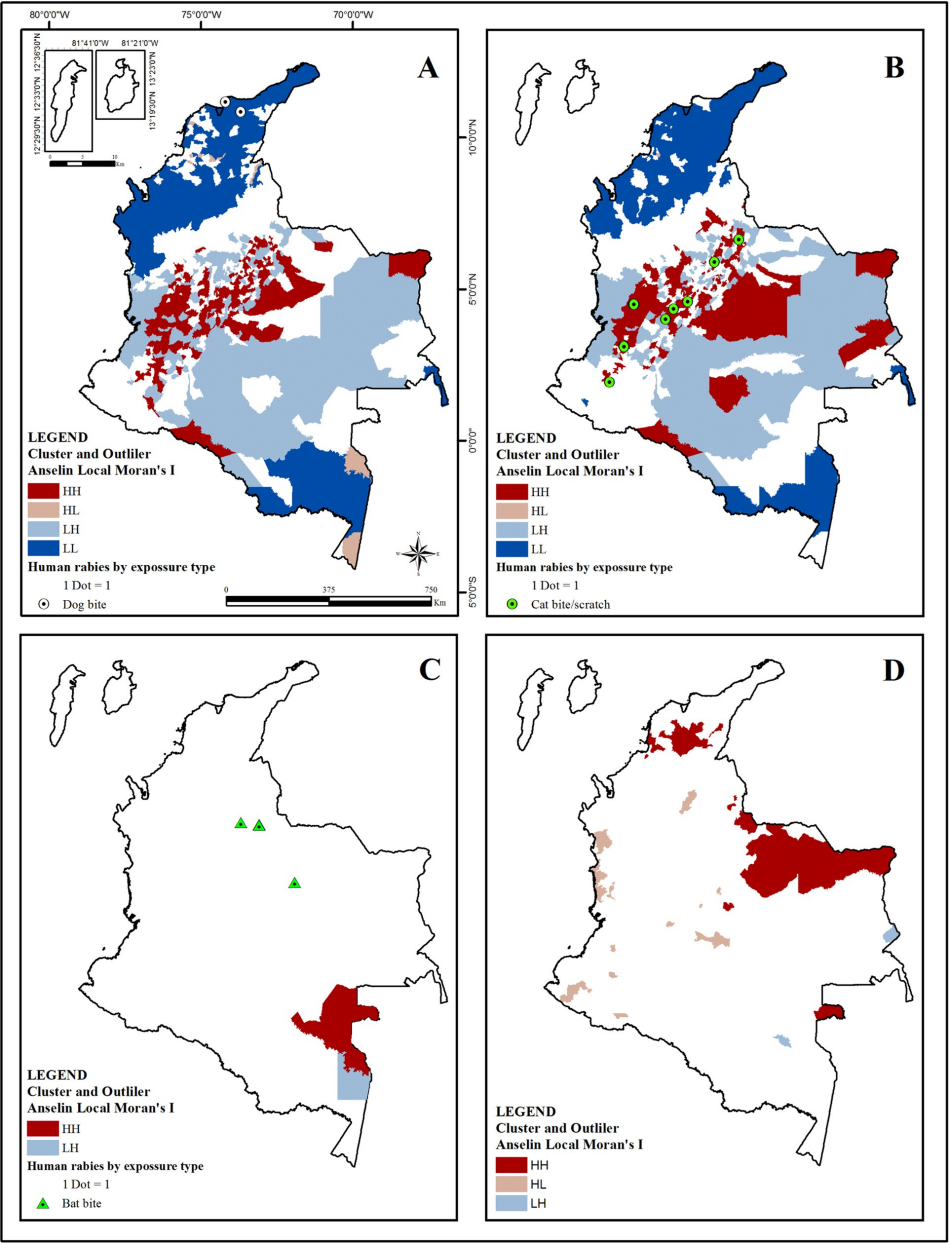
Distribution of cluster and outlier analysis (Anselin Local Moran’s I) for HRE by aggressor specie and HDR according to exposure type in Colombia (2007–2016). (A) Cluster and Outlier (Anselin Local Moran’s I) for HRE due to dog with HDR transmitted by dog bite. (B) Cluster and Outlier (Anselin Local Moran’s I) for HRE due to cat with HDR transmitted by cat bite or scratch. (C) Cluster and Outlier (Anselin Local Morans I) for HRE due to bat with HDR transmitted by bat bite. (D) Cluster and Outlier (Anselin Local Moran’s I) for HRE due to farm animals. Cluster and Outlier levels according to Anselin Local Moran’s I classification: HH (High-high Cluster), HL (High-low Outlier), LH (Low-high Outlier) and LL (Low-low Outlier). The colorless areas have no statistical significance. The colored dot of HDR represents the variant involved: White for V1 variant of dog and Green for variants of bat (V3, V4, and atypical).

Moran’s global index indicated significant spatial clustering of incidence rates for all aggressor species. Full and complete details of Spatial Autocorrelation Report are provided in the appendix (S1 Appendix). Low-Low cluster of HRE due to dogs occurred in the Caribbean region that present the two cases of HDR related to dog aggression (Fig 5A); all cases of HDR transmitted by bats occurred in cities without statistical significance for HRE due to bats (Fig 5C) and all HDR transmitted by cats occurred in the HH for HRE due to cats located in the Andean region (Fig 5B).

The HH and HL showed a 33.6% of the total of the analyzed cases (224,051/666,411cases) located in the 59.4% of Colombia Cities (667/1122 cities) and in the 42.5% of the national area (487,145/1,143,402 Km^2^) (Table 2). The cities were observed in a 47.3% (316/667cities) in HRE due to dogs (Fig 5A), 43.1% (288/667cities) in HRE due cats (Fig 5B), 8.8% (59/667cities) in HRE due to farm animals (Fig 5D) and 0.5% (4/667cities) in HRE due to bats (Fig 5C).

**Table 2:** Sociodemographic and geographical description of high HRE incidence by epidemiological scenario in Colombia (2007–2016).

VARIABLES	EPIDEMIOLOGICAL SCENARIOS						
	URBAN		RURAL	AMAZONIAN	INEQUALITY		
	HH DOG	HH CAT	HH FARM ANIMALS	HH BAT	HL DOG	HL CAT	HL FARM ANIMALS
N = 224,051	n = 181,540 (%)	n = 34,370 (%)	n = 2,241 (%)	n = 391 (%)	n = 4,670 (%)	n = 134 (%)	n = 705 (%)
**AGE**							
0–9 years	47,423 (26.1)	7,242 (21.1)	125 (5.6)	153 (39.1)	1,389 (29.7)	23 (17.29	183 (26.0)
10–19 years	38,636 (21.3)	5,357 (15.6)	337 (15.0)	109 (27.9)	1,090 (23.3)	24 (17.9)	158 (22.4)
20–29 years	22,813 (12.6)	4,690 (13.6)	407 (18.2)	50 (12.8)	484 (10.4)	19 (14.2)	108 (15.3)
30–39 years	18,327 (10.1)	3,730 (10.9)	485 (21.6)	26 (6.6)	446 (9.6)	15 (11.2)	96 (13.6)
40–49 years	16,668 (9.2)	3,877 (11.3)	417 (18.6)	17 (4.3)	394 (8.4)	19 (14.2)	69 (9.8)
50–59 years	15,868 (8.7)	3,748 (10.9)	256 (11.4)	23 (5.9)	399 (8.5)	14 (10.4)	50 (7.1)
60–69 years	11,294 (6.2)	2,694 (7.8)	152 (6.8)	9 (2.3)	238 (5.1)	8 (6.0)	29 (4.1)
70–79 years	7,412 (4.1)	1,875 (5.5)	47 (2.1)	4 (1.0)	163 (3.5)	9 (6.7)	10 (1.4)
80 years and over	3,073 (1.7)	1,151 (3.3)	15 (0.7)	0 (0.0)	67 (1.4)	3 (2.2)	2 (0.3)
Without information	26 (0.0)	6 (0.0)	0 (0.0)	0 (0.0)	0 (0.0)	0 (0.0)	0 (0.0)
**GENDER**							
Female	79,932 (44.0)	21,019 (61.2)	357 (15.9)	171 (43.7)	1,853 (39.7)	76 (56.7)	244 (34.6)
Male	101,608 (56.0)	13,351 (38.8)	1,884 (84.1)	220 (56.3)	2,817 (60.3)	58 (43.3)	461 (65.4)
**AGRESSION TYPE**							
Bite	168,224 (92.7)	27,227 (79.2)	470 (21.0)	390 (99.7)	4,238 (90.7)	110 (82.1)	410 (58.2)
Scratch	12,668 (7.0)	6,985 (20.3)	34 (1.5)	1 (0.3)	412 (8.8)	24 (17.9)	2 (0.3)
Contact of mucosa or skin injured with saliva, nervous tissue, biological material, or secretions infected with rabies virus	645 (0.4)	158 (0.5)	1,737 (77.5)	0 (0.0)	20 (0.4)	0 (0.0)	293 (41.5)
Others	3 (0.0)	0 (0.0)	0 (0.0)	0 (0.0)	0 (0.0)	0 (0.0)	0 (0.0)
**OCUPATION**							
Student	64,77 (44.6)	8,978 (26.1)	324 (14.5)	135 (34.5)	1,926 (41.2)	40 (29.9)	192 (27.2)
Homemaker	26,856 (14.8)	7,928 (23.1)	179 (8.0)	37 (9.5)	721 (15.4)	43 (32.1)	60 (8.5)
Underage	1,793 (1.0)	3,271 (9.5)	64 (2.9)	84 (21.5)	442 (9.5)	7 (5.2)	141 (20.0)
Professionals, technicians, and workers from the agroforestry and livestock areas	9,842 (5.4)	815 (2.4)	957 (42.7)	66 (16.9)	375 (8.0)	3 (2.2)	182 (25.8)
Others	62,142 (34.2)	13,378 (38.9)	717 (32.0)	69 (17.6)	1,206 (25.8)	41 (30.6)	130 (18.4)
**ETHNICITY**							
Indigenous	3,184 (1.8)	221 (0.6)	69 (3.1)	385 (98.5)	519 (11.1)	0 (0.0)	58 (8.2)
Afrodescendant	5815 (3.2)	993 (2.9)	105 (4.7)	0 (0.0)	132 (2.8)	1 (0.7)	376 (53.3)
Others	126 (0.1)	237 (0.7)	36 (1.6)	1 (0.3)	16 (0.3)	0 (0.0)	3 (0.4)
Population without ethnicity	171,281 (94.3)	32,919 (95.8)	2031 (90.6)	5 (1.3)	4,003 (85.7)	133 (99.3)	268 (38.0)
**ZONE**							
Urban	141,926 (78.2)	30,564 (88.9)	671 (29.9)	141 (36.1)	4,026 (86.2)	122 (91.0)	129 (18.3)
Rural	39,614 (21.8)	3,806 (11.1)	1,570 (70.1)	250 (63.9)	644 (13.8)	12 (9.0)	576 (81.7)
MUNICIPALITIES BY REGION*							
**MUNICIPALITIES N = 667**	n = 304 (%)	n = 285 (%)	n = 48 (%)	n = 4 (%)	n = 12 (%)	n = 3 (%)	n = 11 (%)
Amazonian	2 (0.7)	3 (1.1)	1 (2.1)	4 (100.0)	2 (16.7)	0 (0.0)	0 (0.0)
Andean	277 (91.1)	253 (88.8)	9 (18.8)	0 (0.0)	1 (8.3)	0 (0.0)	5 (45.5)
Caribbean	0 (0.0)	0 (0.0)	22 (45.8)	0 (0.0)	9 (75.0)	3 (100.0)	0 (0.0)
Orinoquia	25 (8.2)	29 (10.2)	16 (33.3)	0 (0.0)	0 (0.0)	0 (0.0)	2 (18.2)
Pacific	0 (0.0)	0 (0.0)	0 (0.0)	0 (0.0)	0 (0.0)	0 (0.0)	4 (36.4)
**AREA Km**^**2**^ **N:1,143,407**	124,957 (10.9)	178,113 (15.6)	106,365 (9.3)	41,182 (3.6)	15,253 (1.3)	292 (0.0)	20,985 (1.8)
POPULATION** N = 46,322,690	7,896,000 (17.0)	11,873,761 (25.6)	852,829 (1.8)	38,001 (0.1)	160,891 (0.3)	54,002 (0.1)	218,274 (0.5)
Population Density	63.2	66.7	8.0	0.9	10.5	185.0	10.4

HH: High-High Cluster HL: High-Low Outlier

*The insular region was excluded because no municipalities’ clustering or outliers were observed.

**Population mean determined by municipalities and corregimientos from census projection in Colombia during 2007–2016.

**Fig 6:**
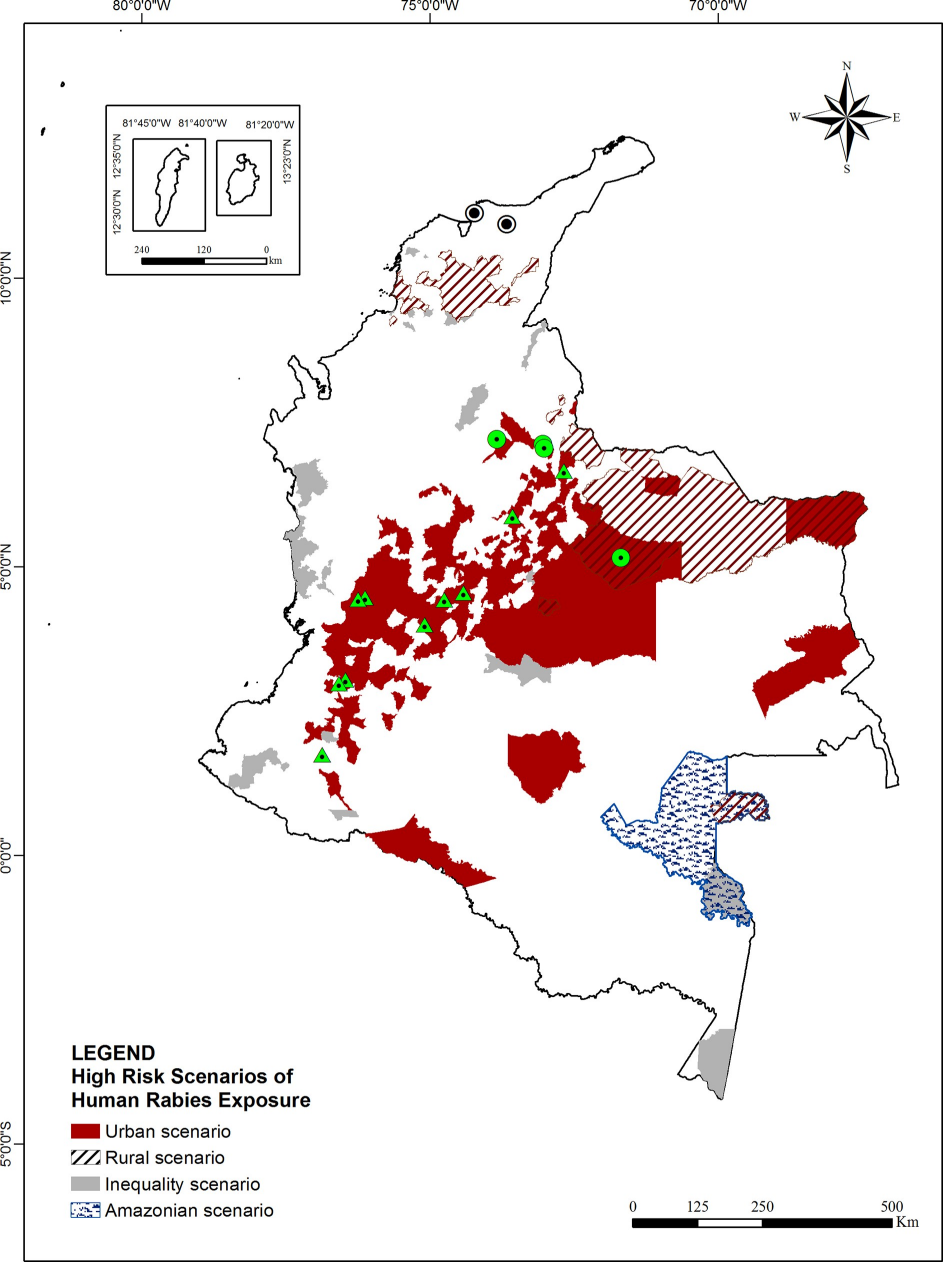
High-incidence scenarios of HRE in Colombia 2007–2016. The colored dot of HDR represents the variant involved: White for V1 variant of dog and Green for variants of bat (V3, V4, and atypical).

Cluster and Outlier Analysis (Anselin Local Moran’s I) showed various areas of high-incidence (Fig 5). Once analyzed the values of HH and HL variables by HRE and the trend and spatial localization of HH and HL, four scenarios were defined according to similar characteristics (Fig 6).

The first scenario was called Urban (Fig 6). This scenario was located in the most cities of HH cluster, corresponding to the cities where the HRE incidence due to dogs and cats occurred (45%; 304/667 and 42.7%; 285/667 respectively). It was observed in cities with high population density (63.2 habitants/Km^2^ and 66.7 habitants/ Km^2^ respectively) in Andean region (91.1%; 277/304 and 88; 253/285 cities respectively), Orinoquia region (8.2%; 25/304 and 10.2%; 29/285 cities respectively) and Amazonian Region (0.7%; 2/304 and 1.1%; 3/285 respectively), with the cases of HRE due dogs and cats notified mainly in urban area (78%; 141,926/178,540 cases and 88,9%; 30,564/34,370 cases respectively) (Table 2). Children aged 0 to 9 years old were most attacked (26.1%; 47,423/181540 by dog and 21.1%; 7,402/34,370 by cat), HRE due to cats were most observed in woman (61.1%; 21,019/34,370) and due to dogs in men (56%; 101,608/181,540), students were the occupation more related (44.6%; 64,770/181,540 cases by dogs and 26.1%; 8978/34,370 cases by cats) and the most frequent aggression type was bite by dogs with 92,7% (168,224/181,540) and by cats with 79.2% (27,227/ 34,370). Population without ethnicity was more than 94% for both. All HDR caused by HRE due to cats and bats were observed in this scenario in the years 2007, 2008, 2009, 2010, 2012, 2015 and 2016 (Fig 3).

The second scenario was called Rural (Fig 6). It was observed for HH of HRE incidence due to farm animals in cities with dispersed population (8.0 habitants/Km^2^) of Caribbean (45.8%; 22/48cities), Orinoquia (33.3%; 16/48cities), Andean (18.8%; 9/48cities) and Amazonian regions (2.1%; 1/48cities) with most cases located in rural area (70.1%; 1570/2241) (Fig 6) (Table 2). The Caribbean region registered only HH for farm animals (Fig 5D). The trend fluctuated for HRE due to farm animals (Fig 2B) and this scenario showed one case of HDR caused by HRE due to bat in 2007. The highest clustering-incidence of HRE due to farm animals was observed mainly among professionals, technicians, and workers from the agroforestry and livestock area (42.7% - 957/2,241), who were more frequently 30 to 39 years old (21.6% - 485/2,241) and men (84.1%; 1,884/2241), with the most frequent aggression type being contact of mucosa or skin injured with saliva infected with rabies virus (74.5% - 1,669/2,241) and more than 90% without ethnicity (Table 2).

A third scenario was observed and was called Amazonian. This showed HH cluster for HRE due to bats, exclusive in cities of the Vaupés department in Amazon Region (100%; 4/4 cities) (Fig 5C) and the highest incidence rate of HRE in Colombia (1,100.5/100,000 habitants by HRE due to bats) (Fig 4C). The Amazonian Scenario (Fig 6) shows the students as the most attacked by bats (34.5% - 135/391). Indigenous ethnicity was the population most affected (98.5%; 385/391) mainly in aged 0–9 years old (39.1%; 153/391). These cases fluctuated in time and were recorded principally in rural area (63.9%; 250/391) of cities with the most dispersed population (0.9 habitants/km^2^) observed in the study (Table 2).

Finally the last scenario was called Inequality (Fig 6). It points to HRE due to dogs, cats and farm animals present in outlier cities in the Pacific, Andean, Amazonian, Caribbean and Orinoquia regions. Students who were the most frequent exposed to dog bite lived in isolated municipalities with dispersed population (10.5 habitants/km^2^) in Caribbean (0.8%; 9/1122) and the Amazonian region (0.2%; 2/1122) with 85.7% (4003/4670) belonging to population without ethnicity. Homemaker (32%; 43/134) and students (29%; 40/134) were the most affected by HRE by cat bite (82%; 110/134) in urban area (91%; 122/134) of three cities in the Caribbean region with the highest density population in the study (185 habitants/km^2^). Additionally, students (27.2%; 192/705) were exposed to bite (58.2% 410/705) and contact with mucosa or skin injured with saliva of farm animals infected with rabies virus(41.5%; 293/705) in the Pacific region (0.2%; 4/1,122) and Andean region (0.4%; 5/1,122) registering the most frequency in Afro-descendants (55%;376/705). This is the only scenario present in Pacific region.

None of the high risk scenarios of HRE was related to HDR caused by dog aggression in the cities in Caribbean region.

## Discussion

Incidence rates with values present between Q1 to Q4 were distributed geographically for most of the national territory for HRE due to dogs and cats indicating the underreporting present in a large part of the national territory for other aggressor species where several cities were observed without incidences, principally in the cities of the Amazonian and Caribbean regions (Fig 4).

The major scenario was the Urban Scenario. It present a situation similar to worldwide, where is most reported dog bites in children and cat bites among women [44]. According to DANE, in the Andean region is located most of the principal cities in Colombia and reported the highest human population growth and the highest population density in the last seven years. Also the MSPS showed the increase in the growth of dog and cat population in urban areas of principal cities of Colombia [45, 46] which would explain the greater concentration of domestic animals there. The lowest AIMPR in Andean and Orinoquia region indicated better quality of life conditions and higher chances of receiving medical attention that can be seen reflected in the increase of HRE notifications due dogs and cats in Colombia in this scenario during the analyzed period [47]. Additionally, the HDR transmitted by cats and bats bite, only were reported in Andean and Orinoquia Region [48–50]. It could have increased the number of notifications of HRE, like a population response to education programs and TV news about HDR in this area. Here, we observed how the cat makes a difference in rabies transmission, as the main transmitter of wild rabies variants to humans in Colombia [11–13], different from others countries that are usually transmitted by bat bite [51]. This is probably occurring due to the urban expansion in the Andean and Orinoquia regions that have modified the use of peri-urban and rural land. Two phenomena can be observed there: large population migrations to peripheral areas in search of job and low land costs for urbanization, and a high demand for rural land near Colombian principal large cities for the construction of country houses and places for tourist and recreational activities [52,53]. In this urban-rural transition zone, cats are in close contact with bats that inhabit the Andean and Orinoquia regions, mainly in municipalities with wild rabies circulation (rural scenario) and where the cases of HDR occurred due to bat bite [15].

Rural scenario was observed mainly in the Orinoquia and Caribbean regions, which present the highest livestock population in the country [54,55]. The distribution of wild rabies outbreaks in farm animals were presented in Caribbean, Andean, Pacific, and Orinoquia regions according to the Colombian Agricultural Institute (ICA) [56,57]. Notification of HRE due to farm animals is made when a person at risk of becoming infected with rabies virus is identified. The rabies surveillance protocol indicates that when an animal is diagnosed with rabies virus, both SIVIGILA and the animal health surveillance system conduct an active search of people who were in contact with the animal, evaluating the type of contact and making the medical treatment according to the risk [38]. In fact, HRE in farm animals showed a similar trend than focus of farm animals diagnosed with rabies virus in Colombia during the same period [58,59], showing the importance of protocol compliance. The similarities demonstrated that people affected did not have appropriate animals management practices, when animals present nervous symptomatology [56], and the High incidence by HRE due to farm animals was caused for wild rabies outbreaks in farm animals. The great importance of this scenario is confirming the closeness of wild rabies with urban scenario, and that both scenarios share cities were cases of HDR by bats occur.

The increase of the incidence rate of HRE due to bat in the Amazon scenario could be related to the implementation of the strategy model of surveillance, prevention, and control of wild rabies in high-risk communities where a pilot project was conducted with the objective of applying the human rabies pre-exposure vaccination scheme in dispersed populations of difficult access in five departments (Chocó, Cauca, Vaupés, Vichada and Nariño) of Colombia during the years from 2012 to 2015. The final report indicated that people who lived in cities with dispersed population in departments of Cauca, Vaupés, Vichada and Nariño received human rabies vaccination and there were notifications and reports about people attacked by bats [21,22,60]. The execution of this project provided an opportunity to show a high incidence of HRE due to bats bite in an area where access is difficult, without communication routes, with low access to education and information media, mainly inhabited by indigenous population in the Amazon rainforest and where access to health services is of high cost for population [35] This project was conducted only in the Vaupés department of the Amazonian region. Therefore, the other cities that showed the most low incidence rates for all type of aggressor animals raise questions about the real vulnerability of indigenous population who are part of more than 60% of the population present in the Amazon region [35]. This scenario shows an area that may have been displayed to SIVIGILA by a non-continuous prevention project realized in populations of difficult access that would be worthwhile to study more thoroughly. In addition, the values reported by the department of Vaupés considerably exceed the distribution of the incidences rates by quartiles shown in Fig 4, where Q4 represented high rate incidences at the national level, being the rank of this quartile from 2/100,000 to 1,100/100,000 habitants. Of this range, only the municipalities of the department of Vaupés were superior to the 10 /100,000 habitants, excluding the other municipalities where the observed high rate of incidence was classified according to quartiles and excluded cases of HDR due to bat bite from areas of high incidence rates classified by quartiles.

The scenario Inequality is present near the cities that reported the lower incidence of HRE for all the studied periods; the lower incidence for HRE due to cats, dogs, and bats; and also the cities with the highest AIMPR in Colombia (Pacific and Caribbean region) [47]. In Caribbean region, HRE due to dog and cat showed a good surveillance system in cities that are located nearseveral cities with underreporting to SIVIGILA. It is possible that local campaigns focused principally on prevention of rabies transmitted by dogs and cats bite in these cities, where Colombia has historically presented HDR due dogs [30]. However, Caribbean region did not have an increase as would be expected in incidence rate of HRE in the years of analysis despite having presented HDR caused by dog. In Pacific region, only HRE due to farm animals were observed for all High incidence evaluated for HRE, specifically in Chocó department. Overall, HRE due to farm animals occurs due to outbreaks of animal rabies and the patient was sought for medical treatment [38]. The population here is different from the rural scenario because they are related to ethnic groups that lives in the department with the most HDR by bat bite in Colombia history [33] and that are sustained by agricultural production carried out in forest areas, who live far from health centers in areas with rabies viral circulation, with high poverty rate, with difficult access to health information and probably with child labor involved to help in the family economy [35]. These characteristics expose them to be in contact with RABV and also illustrate why they are not looking for medical attention when they are exposed to RABV due to animals other than farm animals. Therefore, this low incidence for all HRE could be the result of a population with high vulnerability that may not be receiving medical attention due to problems of public order [33], their levels of poverty and difficult access to health [13].

It is possible that the underreporting of cases may modify the reality of the scenarios found, taking into account that a large part of the national territory has incidence rates at level Q1 or does not present incidence rates (both by animal aggressor and in the behavior of the notification during the 10 years analyzed).

## Conclusions

Spatiotemporal analysis with specific sociodemographic characteristics provided us the opportunity to determine four epidemiological scenarios for high incidence of human rabies exposures with human deaths by rabies notified to SIVIGILA in Colombia. These scenarios allowed us to visualize critical areas where the lack of knowledge of the population about forms of transmission and reservoirs of rabies virus, the geographical difficulties and barriers to access to healthcare in indigenous and afrodescendant communities, did not allow a good notification of the cases of HRE to SIVIGILA.

Ethno-cultural education campaigns and improving training in animal management practices could increase the notification of human rabies exposure in all Colombia regions. However, improving the patient's conditions to access medical care is a more complex and necessary action to decrease the underreporting.

The HRE due to cats is positioned as the main form of transmission of rabies variants of wild origin to humans, in regions where the notification of HDR had not been reported or was not frequent. This change completely modifies the history of human rabies in Colombia and demonstrates the adaptability of the virus to different mammal species and environments. Studies of the speed of displacement and Rabies viral circulation in all regions, mainly in the Amazon region are required, where the indigenous population is the most exposed to the principal reservoir of rabies variants of wild origin, the bats.

Finally, It is important to highlight Colombia as a multicultural country and with great geographical differences. Further investigation into the functionality of national rabies surveillance and prevention plans at the regional level and by animal aggressor type is necessary, where variables as ethnical population, risk factors in children, traditional knowledge, economic conditions and location of health centers can be analyzed, and where possible, include alternatives of differential approach on vulnerable populations in the Rabies protocol with the objective to break the barrier between the patient and opportune medical care.

## Supporting information

S1 Table Incidence rates of HRE per 100.000 habitants by year in all cities of Colombia, 2007–2016. (PDF)

S2 Table Mean incidence of HRE by animal aggressor type in all cities of Colombia (2006–2017). (PDF)

S1 Appendix Spatial autocorrelation report. (PDF)
