# Supplementary material for: Epidemiological scenarios for human rabies exposure notified in Colombia during ten years: A challenge to implement surveillance actions with a differential approach on vulnerable populations
Source: PLoS One. 2019 Dec 27;14(12):e0213120. doi: 10.1371/journal.pone.0213120 (PMC6934280; doi:10.1371/journal.pone.0213120)
Supplement: S1 Table — (PDF) [file pone.0213120.s001.pdf]

S1_TABLE. Incidence rates of HRE per 100.000 habitants by year in all cities of Colombia, 2007-2016.

DEPARMENT	CITY	2007	2008	2009	2010	2011	2012	2013	2014	2015	2016
Amazonas	El Encanto	0,00	0,00	21,88	0,00	85,78	0,00	0,00	0,00	20,66	0,00
Amazonas	La Chorrera	0,00	0,00	0,00	27,72	0,00	0,00	26,53	26,15	0,00	25,43
Amazonas	La Pedrera	25,33	0,00	0,00	46,31	0,00	0,00	84,91	20,64	0,00	19,51
Amazonas	La Victoria	0,00	0,00	0,00	0,00	0,00	0,00	0,00	0,00	0,00	0,00
Amazonas	Leticia	101,01	5,13	185,67	302,52	297,44	361,91	334,37	348,78	510,57	384,26
Amazonas	Miriti - Paraná	123,99	0,00	0,00	0,00	0,00	0,00	0,00	65,02	65,32	0,00
Amazonas	Puerto Alegría	0,00	0,00	0,00	0,00	60,79	116,62	55,93	0,00	0,00	0,00
Amazonas	Puerto Arica	0,00	0,00	0,00	0,00	71,99	0,00	0,00	0,00	0,00	0,00
Amazonas	Puerto Nariño	0,00	0,00	13,41	13,20	130,02	76,84	126,18	49,73	98,02	277,81
Amazonas	Puerto Santander	80,65	0,00	0,00	75,59	0,00	0,00	0,00	0,00	0,00	33,43
Amazonas	Tarapacá	0,00	0,00	151,90	25,05	0,00	24,55	0,00	120,37	119,19	23,62
Antioquia	Abejorral	0,00	14,98	4,99	74,92	134,85	134,85	189,79	124,86	184,80	214,76
Antioquia	Abriaquí	0,00	0,00	0,00	0,00	0,00	43,67	89,65	459,77	375,94	240,96
Antioquia	Alejandría	0,00	26,85	27,11	136,91	27,64	0,00	84,51	114,22	173,11	320,23
Antioquia	Amagá	0,00	146,69	188,00	137,16	146,53	131,50	185,46	204,51	426,32	302,32
Antioquia	Amalfi	0,00	76,24	56,77	4,70	0,00	92,53	142,41	145,91	167,51	197,73
Antioquia	Andes	0,00	137,68	50,82	144,12	185,73	177,24	171,13	184,95	157,16	190,39
Antioquia	Angelópolis	0,00	24,89	24,54	0,00	35,65	233,89	138,25	113,56	44,71	55,05
Antioquia	Angostura	0,00	16,43	58,11	83,82	50,80	94,06	103,64	148,29	211,38	160,14
Antioquia	Anorí	0,00	70,49	101,19	87,36	86,22	18,24	114,06	106,70	163,88	132,92
Antioquia	Anza	0,00	13,39	26,72	40,00	26,61	92,97	172,35	79,41	0,00	79,16
Antioquia	Apartadó	0,00	18,02	27,56	26,74	12,65	86,55	103,04	135,83	135,20	117,57
Antioquia	Arboletes	0,00	122,81	119,56	110,76	99,59	153,54	173,23	199,42	137,00	82,51
Antioquia	Argelia	0,00	0,00	21,02	85,27	140,69	186,65	89,21	226,32	183,93	128,24

Antioquia	Armenia	0,00	123,00	251,31	235,39	262,12	379,13	342,23	255,99	237,53	316,30
Antioquia	Barbosa	0,00	8,96	15,41	86,62	112,88	270,32	344,31	257,74	343,64	365,88
Antioquia	Bello	0,00	51,68	69,89	73,82	77,57	93,95	115,83	167,49	165,18	157,55
Antioquia	Belmira	0,00	31,42	140,19	123,48	76,52	30,35	15,05	119,24	29,59	87,94
Antioquia	Betania	0,00	60,13	131,57	61,36	41,29	281,66	158,11	276,95	193,84	130,61
Antioquia	Betulia	0,00	105,93	111,23	0,00	139,17	225,21	224,02	177,39	193,82	136,32
Antioquia	Briceño	0,00	34,24	11,42	11,44	34,34	126,03	137,61	91,84	91,93	126,55
Antioquia	Buriticá	0,00	0,00	0,00	73,64	59,19	89,34	134,55	210,43	196,94	182,76
Antioquia	Cáceres	0,00	6,38	3,11	51,45	64,80	25,81	22,33	86,95	15,87	30,89
Antioquia	Caicedo	0,00	25,54	63,40	63,01	75,08	74,53	209,90	73,52	36,56	169,18
Antioquia	Caldas	0,00	170,10	116,48	9,58	159,31	202,58	190,83	122,21	274,90	261,57
Antioquia	Campamento	0,00	0,00	21,19	74,58	32,15	32,36	43,44	65,58	187,00	88,54
Antioquia	Cañasgordas	0,00	77,25	130,80	101,14	136,84	172,69	131,11	125,17	89,48	161,18
Antioquia	Caracolí	0,00	167,33	168,39	423,19	404,26	321,13	172,19	281,63	239,39	175,09
Antioquia	Caramanta	0,00	54,75	91,59	165,41	92,03	184,84	203,89	316,57	130,55	224,72
Antioquia	Carepa	0,00	49,28	25,06	12,21	53,56	131,50	143,27	99,26	100,38	173,02
Antioquia	Carolina	0,00	0,00	0,00	0,00	26,60	241,03	81,17	109,53	192,89	167,46
Antioquia	Caucasia	0,00	71,97	66,07	31,22	22,60	23,01	84,20	53,88	49,03	77,46
Antioquia	Chigorodó	0,00	54,90	116,18	90,91	114,74	134,47	173,91	133,23	140,42	165,07
Antioquia	Cisneros	0,00	241,95	169,51	245,33	322,06	216,29	43,52	252,17	209,76	355,63
Antioquia	Ciudad Bolívar	64,17	82,33	150,98	90,26	25,38	123,83	146,33	69,83	140,30	211,45
Antioquia	Cocorná	0,00	26,54	13,27	0,00	66,51	6,66	26,65	33,38	26,72	106,92
Antioquia	Concepción	0,00	119,85	196,90	303,18	25,97	266,24	300,63	224,28	288,77	266,67
Antioquia	Concordia	0,00	150,90	0,00	9,49	180,97	248,54	206,30	154,24	145,26	179,92
Antioquia	Copacabana	0,00	117,23	29,29	19,76	12,00	167,29	173,89	285,71	326,35	313,93
Antioquia	Dabeiba	0,00	50,15	50,29	71,44	63,23	84,59	127,33	85,21	64,16	55,84
Antioquia	Don Matías	0,00	199,85	179,87	115,53	112,91	81,62	65,74	82,69	31,47	39,60

Antioquia	Ebéjico	0,00	87,93	175,79	135,78	103,88	159,80	223,54	167,70	223,73	127,90
Antioquia	El Bagre	0,00	27,57	88,39	114,88	109,93	70,00	75,64	140,11	38,32	76,13
Antioquia	El Carmen de Viboral	0,00	84,37	53,20	88,99	283,76	248,93	94,34	236,10	440,63	414,03
Antioquia	El Santuario	0,00	33,86	11,25	26,16	182,60	156,08	126,01	140,43	143,81	401,09
Antioquia	Entrerrios	0,00	134,56	88,35	217,18	128,26	84,20	217,53	81,57	120,60	148,49
Antioquia	Envigado	0,00	40,43	15,05	17,72	91,42	105,17	127,66	146,77	154,19	175,71
Antioquia	Fredonia	0,00	151,77	192,93	121,79	194,97	401,17	247,51	248,99	23,19	219,36
Antioquia	Frontino	0,00	52,82	32,29	93,18	94,90	119,41	162,22	230,20	216,67	208,45
Antioquia	Giraldo	0,00	0,00	0,00	0,00	24,45	269,81	270,67	24,73	0,00	124,72
Antioquia	Girardota	0,00	178,53	240,09	271,64	275,31	320,27	332,16	315,06	326,33	315,37
Antioquia	Gómez Plata	0,00	127,65	92,46	0,00	8,19	8,10	15,98	86,87	117,10	169,70
Antioquia	Granada	0,00	10,19	91,67	111,97	40,72	30,49	10,16	81,18	131,86	91,22
Antioquia	Guadalupe	0,00	159,92	79,87	95,74	223,14	95,53	190,87	238,32	47,62	15,86
Antioquia	Guarne	0,00	88,21	137,98	6,88	4,50	37,57	316,73	383,38	179,93	435,69
Antioquia	Guatapé	0,00	422,83	88,92	89,83	562,92	311,47	537,63	674,41	1117,64	974,96
Antioquia	Heliconia	0,00	108,81	62,97	222,89	161,06	114,04	181,52	133,67	253,98	137,06
Antioquia	Hispania	0,00	0,00	0,00	41,28	268,10	288,42	452,77	246,71	349,15	266,72
Antioquia	Itagui	0,00	78,56	74,32	44,42	85,37	102,89	140,64	159,00	185,92	170,17
Antioquia	Ituango	0,00	67,28	77,05	47,93	62,12	94,82	96,52	126,33	100,02	121,19
Antioquia	Jardín	0,00	84,35	84,64	148,86	142,42	186,10	309,35	274,69	429,15	329,12
Antioquia	Jericó	0,00	214,17	191,46	152,41	161,37	243,43	253,08	410,78	446,17	582,56
Antioquia	La Ceja	0,00	136,84	8,18	6,06	253,23	291,31	322,67	337,88	288,30	301,72
Antioquia	La Estrella	0,00	77,48	49,58	26,11	56,49	178,45	202,03	223,25	261,44	247,89
Antioquia	La Pintada	0,00	274,53	203,84	87,96	368,95	312,50	435,11	695,92	335,47	215,15
Antioquia	La Unión	0,00	152,86	97,64	96,96	149,93	212,85	248,61	268,28	177,83	358,83
Antioquia	Liborina	0,00	0,00	0,00	0,00	31,58	84,15	52,55	63,00	20,98	188,56
Antioquia	Maceo	0,00	242,49	149,82	151,43	222,87	168,97	242,13	259,48	218,82	265,68

Antioquia	Marinilla	2,13	6,28	123,49	76,98	155,50	176,63	189,31	157,92	153,63	225,15
Antioquia	Medellín	0,04	37,58	48,81	28,60	74,78	124,19	142,80	148,95	146,04	207,34
Antioquia	Montebello	0,00	0,00	0,00	131,43	89,34	91,21	31,01	47,44	80,68	32,91
Antioquia	Murindó	0,00	0,00	0,00	0,00	0,00	23,17	22,72	44,52	43,54	0,00
Antioquia	Mutatá	0,00	5,67	0,00	37,97	116,67	202,24	111,60	158,68	92,18	208,76
Antioquia	Nariño	18,88	93,48	141,89	24,42	66,45	53,80	94,60	128,66	150,37	114,38
Antioquia	Nechí	0,00	26,87	0,00	76,66	29,06	28,34	51,36	107,94	41,37	51,40
Antioquia	Necoclí	0,00	98,06	88,03	87,59	92,47	119,53	128,31	93,78	104,23	157,83
Antioquia	Olaya	0,00	100,03	32,98	0,00	32,26	63,86	63,11	312,79	278,04	274,56
Antioquia	Peñol	0,00	6,18	49,55	74,49	105,79	218,48	412,89	420,41	446,85	283,95
Antioquia	Peque	0,00	0,00	19,71	19,47	105,66	94,91	131,22	148,16	128,15	72,31
Antioquia	Pueblorrico	0,00	265,69	179,83	65,33	172,83	364,77	371,29	489,92	284,50	274,84
Antioquia	Puerto Berrío	0,00	256,92	292,59	179,78	185,71	204,81	137,05	54,28	89,58	115,26
Antioquia	Puerto Nare	0,00	138,27	131,14	112,81	33,49	99,43	32,81	102,87	42,89	5,31
Antioquia	Puerto Triunfo	0,00	97,79	78,90	82,81	91,93	132,47	103,83	142,45	164,49	131,82
Antioquia	Remedios	0,00	89,49	87,25	116,00	150,89	206,09	143,72	213,91	109,59	113,72
Antioquia	Retiro	0,00	85,00	0,00	27,65	27,35	329,69	219,11	385,92	533,81	440,19
Antioquia	Rionegro	0,00	172,93	63,68	18,13	28,49	164,48	215,84	231,69	251,98	318,25
Antioquia	Sabanalarga	0,00	24,41	170,90	109,86	122,09	24,42	146,50	24,42	97,67	134,29
Antioquia	Sabaneta	0,00	83,33	100,98	8,29	69,39	106,58	136,79	230,67	281,53	310,16
Antioquia	Salgar	0,00	110,85	161,24	100,41	100,75	162,88	169,11	124,47	204,45	222,36
Antioquia	San Andrés de Cuerquía	0,00	0,00	58,04	14,74	90,00	76,27	108,54	236,74	417,60	261,18
Antioquia	San Carlos	0,00	18,92	62,91	56,54	12,54	131,45	137,43	174,61	311,25	223,80
Antioquia	San Francisco	0,00	16,53	50,51	17,13	122,10	88,89	72,45	129,15	206,84	19,15
Antioquia	San Jerónimo	0,00	375,16	529,76	303,63	317,85	420,41	329,16	422,61	474,87	424,39
Antioquia	San José de La Montaña	0,00	221,73	125,87	31,25	155,09	123,08	549,28	241,98	299,76	118,76
Antioquia	San Juan de Urabá	0,00	49,76	53,30	0,00	4,28	189,07	49,48	178,11	23,84	11,69

Antioquia	San Luis	0,00	145,64	163,83	136,70	0,00	18,24	18,25	27,40	91,42	100,57
Antioquia	San Pedro	0,00	0,00	12,58	0,00	8,08	3,97	0,00	65,06	191,79	255,00
Antioquia	San Pedro de Uraba	0,00	60,93	117,51	49,95	92,46	75,32	136,43	122,43	76,73	85,61
Antioquia	San Rafael	0,00	218,32	300,98	188,84	333,89	380,89	489,45	307,20	431,43	387,00
Antioquia	San Roque	0,00	219,58	158,87	183,00	282,40	197,51	327,93	324,73	273,99	348,08
Antioquia	San Vicente	0,00	111,84	10,78	0,00	5,52	5,59	17,00	17,22	75,59	277,01
Antioquia	Santa Bárbara	0,00	129,48	147,76	201,18	92,46	243,84	250,03	305,71	298,97	287,45
Antioquia	Santa Rosa de Osos	0,00	107,80	127,57	8,99	2,96	8,75	20,14	133,52	244,04	263,14
Antioquia	Santafé de Antioquia	4,31	192,69	191,31	189,97	280,83	357,96	351,21	447,29	297,36	404,47
Antioquia	Santo Domingo	0,00	89,13	54,04	54,59	64,37	120,83	253,52	218,53	182,41	252,62
Antioquia	Segovia	0,00	81,86	32,30	63,75	107,46	82,77	140,44	85,72	52,27	34,41
Antioquia	Sonson	0,00	137,60	144,18	145,49	144,10	145,42	199,42	254,55	231,61	216,80
Antioquia	Sopetrán	0,00	86,86	186,49	99,52	169,10	174,50	269,84	329,29	306,21	384,59
Antioquia	Támesis	0,00	352,71	318,19	244,33	409,44	413,98	371,90	376,14	380,12	315,96
Antioquia	Tarazá	0,00	17,06	58,08	61,91	44,51	117,18	7,43	2,41	0,00	11,40
Antioquia	Tarso	0,00	95,97	353,79	107,98	307,77	185,63	262,61	480,89	282,92	178,05
Antioquia	Titiribí	0,00	168,52	94,46	86,49	85,84	170,31	169,03	174,89	125,06	131,09
Antioquia	Toledo	34,46	136,38	67,41	83,28	32,93	0,00	64,31	111,16	125,51	139,19
Antioquia	Turbo	0,00	49,85	48,54	67,32	58,58	40,07	76,08	98,60	50,86	66,66
Antioquia	Uramita	0,00	0,00	0,00	60,42	108,85	84,74	109,05	109,14	48,56	0,00
Antioquia	Urrao	0,00	86,30	80,27	52,77	115,95	158,70	142,74	106,71	127,67	159,06
Antioquia	Valdivia	0,00	80,61	125,81	102,27	164,55	121,57	47,41	87,86	36,07	65,92
Antioquia	Valparaíso	0,00	254,01	254,61	351,33	352,23	481,77	386,54	371,45	437,32	130,04
Antioquia	Vegachí	0,00	9,35	28,52	58,08	68,99	110,38	255,47	249,61	190,52	215,68
Antioquia	Venecia	0,00	470,85	276,84	337,10	457,55	262,88	218,13	195,86	143,36	241,86
Antioquia	Vigía del Fuerte	0,00	0,00	0,00	0,00	0,00	0,00	0,00	0,00	17,90	17,90
Antioquia	Yalí	0,00	0,00	25,27	12,54	186,73	111,14	232,64	230,58	228,42	202,38

Antioquia	Yarumal	0,00	130,41	117,27	108,96	121,02	194,79	98,38	75,59	81,08	200,27
Antioquia	Yolombó	0,00	94,54	78,92	59,26	103,00	114,39	168,52	161,32	212,87	106,63
Antioquia	Yondó	0,00	118,01	127,72	172,74	221,54	137,12	139,92	109,66	26,86	84,25
Antioquia	Zaragoza	0,00	124,56	98,32	114,40	191,60	91,17	123,38	141,61	58,56	73,89
Arauca	Arauca	155,93	11,31	1,24	4,87	157,01	208,93	210,48	260,20	218,13	172,78
Arauca	Arauquita	61,02	18,35	0,00	10,24	93,62	85,03	116,24	244,67	208,19	191,65
Arauca	Cravo Norte	139,20	28,07	0,00	143,14	259,97	320,89	442,48	178,52	330,23	425,14
Arauca	Fortul	115,36	8,74	0,00	25,43	137,76	209,81	178,40	211,80	204,89	163,14
Arauca	Puerto Rondón	305,19	127,62	0,00	77,06	180,32	284,16	440,30	415,37	338,19	469,12
Arauca	Saravena	203,49	0,00	2,24	57,61	234,74	358,65	249,87	284,05	258,46	325,67
Arauca	Tame	137,77	14,24	2,01	35,85	246,40	407,79	328,43	290,78	263,42	298,50
San Andrés	Providencia	1,40	2,77	8,25	4,09	9,47	119,40	272,73	275,72	329,66	378,72
Atlántico	Baranoa	0,00	0,00	14,78	3,65	10,83	33,91	222,45	251,56	292,17	246,47
Atlántico	Barranquilla	0,69	5,72	67,69	77,38	60,49	81,30	147,23	157,39	161,84	157,57
Atlántico	Campo de La Cruz	5,42	5,52	0,00	5,71	5,81	124,18	264,77	367,53	299,25	260,23
Atlántico	Candelaria	16,47	49,20	8,16	48,82	8,11	282,87	265,85	369,63	272,57	391,81
Atlántico	Galapa	0,00	5,71	13,86	5,39	7,85	106,87	249,88	329,68	220,04	218,70
Atlántico	Juan de Acosta	139,93	45,98	6,47	19,14	6,29	105,39	110,04	108,56	166,61	223,12
Atlántico	Luruaco	0,00	0,00	0,00	7,94	3,92	104,40	221,32	339,09	357,06	323,15
Atlántico	Malambo	1,90	5,59	3,66	7,19	17,66	39,04	77,59	167,66	192,12	159,82
Atlántico	Manatí	0,00	0,00	6,87	6,78	6,69	145,30	293,39	321,90	171,72	169,67
Atlántico	Palmar de Varela	0,00	20,66	8,20	20,37	20,23	108,56	227,66	345,35	390,72	368,86
Atlántico	Piojó	0,00	39,58	0,00	0,00	0,00	39,18	234,74	195,05	389,56	194,17
Atlántico	Polonuevo	0,00	0,00	6,91	6,84	6,78	228,36	299,64	488,26	333,70	344,00
Atlántico	Ponedera	0,00	5,02	9,87	9,71	4,78	79,99	333,58	342,28	332,63	256,85
Atlántico	Puerto Colombia	0,00	14,44	28,96	25,41	40,04	36,51	380,83	459,29	439,07	670,64
Atlántico	Repelón	0,00	0,00	0,00	8,19	0,00	87,71	251,77	182,46	226,06	272,32

Atlántico	Sabanagrande	0,00	7,36	3,60	17,59	13,77	20,20	355,71	396,66	306,21	228,88
Atlántico	Sabanalarga	1,12	16,63	9,85	8,65	5,34	59,05	160,47	142,16	95,75	109,83
Atlántico	Santa Lucía	0,00	32,76	8,24	16,60	0,00	126,40	407,54	436,53	250,35	443,79
Atlántico	Santo Tomás	0,00	0,00	0,00	4,06	20,17	236,62	291,22	420,67	335,62	322,21
Atlántico	Soledad	0,81	6,53	9,22	10,08	10,89	39,87	123,72	121,20	95,86	101,39
Atlántico	Suan	0,00	10,61	0,00	43,29	21,86	110,41	346,21	564,46	479,89	519,39
Atlántico	Tubará	54,88	45,67	9,12	18,21	36,39	209,03	226,98	190,55	353,90	471,70
Atlántico	Usiacurí	0,00	0,00	11,04	0,00	10,90	0,00	279,81	407,16	351,44	328,60
Bogotá, D.C.	Bogotá D.C.	76,64	47,94	38,06	41,17	63,77	117,57	190,90	222,93	220,59	240,50
Bolívar	Achí	93,65	160,31	4,79	51,86	217,97	269,25	193,01	123,53	104,12	68,23
Bolívar	Altos del Rosario	67,99	83,46	0,00	0,00	86,87	92,96	75,99	119,28	65,84	35,85
Bolívar	Arenal	0,00	0,00	0,00	5,88	0,00	5,65	44,24	75,75	90,06	103,62
Bolívar	Arjona	190,07	67,47	4,62	25,72	157,45	239,13	22,90	110,99	186,17	117,74
Bolívar	Arroyohondo	0,00	11,00	0,00	0,00	74,26	94,40	165,70	81,78	40,38	89,66
Bolívar	Barranco de Loba	12,81	18,95	43,56	36,75	30,13	23,71	227,13	120,27	140,70	154,74
Bolívar	Calamar	0,00	37,39	87,78	22,84	31,58	22,29	96,83	65,19	85,81	55,06
Bolívar	Cantagallo	123,93	36,56	48,00	11,80	23,19	102,68	269,09	198,41	129,88	191,63
Bolívar	Cartagena	1,10	10,29	12,64	10,06	26,89	21,30	30,45	54,94	70,58	83,88
Bolívar	Cicuco	0,00	0,00	234,81	261,80	153,33	288,68	189,10	378,04	287,82	341,60
Bolívar	Clemencia	270,66	0,00	25,04	0,00	8,23	179,59	113,45	458,09	159,49	316,13
Bolívar	Córdoba	23,26	15,60	47,06	0,00	31,68	7,95	103,78	72,14	48,25	24,22
Bolívar	El Carmen de Bolívar	140,41	1,43	1,42	4,22	182,31	177,56	103,46	146,71	157,02	230,11
Bolívar	El Guamo	51,37	0,00	12,88	12,89	90,16	219,33	450,57	309,16	425,42	205,92
Bolívar	El Peñón	12,37	12,15	47,69	11,69	68,73	89,75	153,86	86,10	200,34	113,47
Bolívar	Hatillo de Loba	17,32	8,63	60,17	0,00	59,66	127,25	194,16	134,37	83,54	124,55
Bolívar	Magangué	177,66	86,59	80,70	100,07	116,96	142,73	230,00	296,88	282,86	259,22
Bolívar	Mahates	243,59	4,23	0,00	8,25	16,31	16,10	75,55	7,85	38,78	72,72

Bolívar	Margarita	0,00	21,17	0,00	41,95	10,43	10,36	0,00	61,27	40,50	150,51
Bolívar	María La Baja	10,94	106,64	99,58	23,67	40,62	67,96	67,50	29,32	62,40	51,61
Bolívar	Mompós	107,36	4,75	82,62	37,54	90,91	187,56	308,10	264,81	213,04	143,95
Bolívar	Montecristo	0,00	5,52	10,81	5,29	0,00	0,00	4,94	81,96	0,00	18,40
Bolívar	Morales	26,32	36,40	20,54	40,57	10,00	34,52	19,45	19,16	9,44	23,25
Bolívar	Norosí	0,00	0,00	0,00	0,00	18,77	0,00	0,00	57,30	19,22	38,63
Bolívar	Pinillos	4,32	0,00	34,02	12,65	41,77	16,54	28,66	20,27	0,00	7,94
Bolívar	Regidor	10,98	32,38	0,00	20,85	10,24	0,00	39,57	19,41	66,74	9,36
Bolívar	Río Viejo	4,67	12,34	12,16	5,99	58,91	46,40	39,97	16,86	27,66	0,00
Bolívar	San Cristóbal	15,24	0,00	0,00	0,00	0,00	90,59	391,39	736,51	554,81	627,43
Bolívar	San Estanislao	6,48	0,00	0,00	12,72	18,96	31,38	105,94	92,89	98,42	85,53
Bolívar	San Fernando	68,78	0,00	0,00	0,00	37,36	14,85	51,64	109,83	50,90	14,44
Bolívar	San Jacinto	0,00	0,00	60,58	0,00	46,61	121,10	32,56	120,82	232,17	366,25
Bolívar	San Jacinto del Cauca	0,00	0,00	16,91	0,00	32,44	15,89	70,00	68,49	37,24	65,58
Bolívar	San Juan Nepomuceno	0,00	15,27	21,32	3,04	33,31	190,21	529,63	422,84	239,05	223,06
Bolívar	San Martín de Loba	204,04	80,21	65,57	25,73	157,60	141,95	169,08	200,95	190,81	146,98
Bolívar	San Pablo	0,00	17,44	0,00	0,00	42,57	153,86	128,62	168,87	90,11	170,42
Bolívar	Santa Catalina	171,75	0,00	0,00	0,00	0,00	0,00	309,38	306,72	250,59	255,68
Bolívar	Santa Rosa	47,38	25,78	25,23	14,82	275,55	397,31	629,11	561,21	424,93	441,46
Bolívar	Santa Rosa del Sur	0,00	0,00	0,00	0,00	116,87	132,16	199,00	146,02	102,37	253,72
Bolívar	Simití	0,00	21,34	0,00	20,97	67,47	133,42	60,83	220,13	64,13	92,34
Bolívar	Soplaviento	108,41	0,00	24,00	107,89	11,96	0,00	59,52	225,57	94,78	11,81
Bolívar	Talaigua Nuevo	35,92	71,76	313,42	339,59	196,08	133,33	274,87	318,08	281,94	201,98
Bolívar	Tiquisio	15,53	20,41	50,30	4,95	97,45	19,16	51,78	50,85	31,76	31,15
Bolívar	Turbaco	174,60	102,17	25,58	98,00	231,40	148,78	162,42	101,16	157,96	102,49
Bolívar	Turbaná	21,85	0,00	0,00	0,00	42,01	131,62	192,10	47,51	161,26	206,24
Bolívar	Villanueva	66,84	49,58	5,44	10,77	202,34	494,48	317,15	668,11	584,02	371,43

Bolívar	Zambrano	0,00	0,00	26,77	124,34	132,50	131,78	322,89	321,04	111,96	136,89
Boyacá	Almeida	0,00	47,24	48,40	148,88	358,61	367,07	540,54	554,94	285,06	117,72
Boyacá	Aquitania	24,41	0,00	18,58	93,64	214,11	279,67	269,63	557,97	590,51	723,77
Boyacá	Arcabuco	0,00	0,00	0,00	0,00	401,84	305,93	114,64	267,33	438,93	514,97
Boyacá	Belén	11,51	11,74	11,96	0,00	12,44	228,54	401,66	608,30	337,84	523,78
Boyacá	Berbeo	0,00	0,00	104,17	156,09	155,93	207,68	363,07	103,63	414,08	258,53
Boyacá	Betéitiva	0,00	0,00	86,10	0,00	89,25	136,43	92,76	94,65	193,33	296,44
Boyacá	Boavita	47,41	12,12	24,73	227,27	348,30	448,25	256,41	289,78	437,91	404,98
Boyacá	Boyacá	161,36	81,83	41,39	62,81	361,70	386,51	457,52	685,54	581,40	362,48
Boyacá	Briceño	36,79	36,97	37,22	74,68	37,66	75,73	152,79	115,12	77,40	77,88
Boyacá	Buenavista	0,00	0,00	0,00	0,00	171,41	446,51	344,12	275,81	259,11	415,44
Boyacá	Busbanzá	322,23	0,00	0,00	0,00	0,00	93,98	273,97	177,46	173,01	419,46
Boyacá	Caldas	0,00	25,43	25,68	233,34	26,27	106,10	375,94	217,45	384,83	361,51
Boyacá	Campohermoso	0,00	0,00	175,75	126,17	177,57	229,77	154,44	103,33	155,97	209,81
Boyacá	Cerinza	0,00	0,00	0,00	0,00	351,14	178,03	309,04	365,82	345,56	430,80
Boyacá	Chinavita	0,00	27,09	54,47	54,76	247,93	443,70	474,60	337,46	425,17	400,11
Boyacá	Chiquinquirá	10,39	13,63	60,35	67,67	141,42	180,94	236,66	216,09	231,33	231,11
Boyacá	Chíquiza	0,00	16,95	0,00	190,28	122,33	159,04	214,25	198,38	164,11	128,84
Boyacá	Chiscas	0,00	0,00	0,00	373,06	381,28	173,76	221,93	272,60	209,74	261,90
Boyacá	Chita	0,00	19,15	0,00	0,00	268,71	554,60	408,75	320,74	303,92	180,72
Boyacá	Chitaraque	0,00	31,30	31,78	177,76	262,77	283,76	407,61	466,24	404,43	464,62
Boyacá	Chivatá	37,94	55,72	54,61	196,15	227,27	188,32	268,77	131,56	80,66	95,01
Boyacá	Chivor	0,00	47,57	0,00	0,00	305,97	52,22	53,39	271,59	278,55	114,16
Boyacá	Ciénega	0,00	0,00	59,14	0,00	20,10	40,62	20,53	249,32	378,63	340,86
Boyacá	Cómbita	0,00	0,00	36,75	21,80	172,22	212,80	189,12	249,26	280,21	276,80
Boyacá	Coper	0,00	0,00	0,00	25,41	334,53	235,36	423,28	402,90	273,00	277,01
Boyacá	Corrales	0,00	0,00	0,00	83,02	294,12	680,85	300,43	390,46	307,96	266,55

Boyacá	Covarachía	31,05	0,00	0,00	64,85	229,96	433,04	406,50	68,63	454,39	566,37
Boyacá	Cubará	45,15	0,00	284,86	44,90	29,89	29,84	89,42	193,51	163,57	29,72
Boyacá	Cucaita	0,00	0,00	0,00	86,15	172,08	300,56	214,36	192,64	234,69	298,70
Boyacá	Cuítiva	150,38	0,00	0,00	661,24	460,36	874,49	310,56	208,44	262,33	369,98
Boyacá	Duitama	4,60	29,26	46,40	66,11	239,83	302,60	322,89	367,95	448,12	447,37
Boyacá	El Cocuy	0,00	0,00	18,35	92,34	241,46	224,51	244,87	170,58	190,80	288,63
Boyacá	El Espino	0,00	0,00	0,00	121,45	314,31	216,71	215,88	71,72	214,54	428,06
Boyacá	Firavitoba	0,00	16,14	0,00	81,71	115,34	231,98	250,17	369,75	220,08	494,54
Boyacá	Floresta	0,00	0,00	21,01	21,17	213,36	279,63	303,82	459,82	198,98	223,31
Boyacá	Gachantivá	133,78	0,00	0,00	34,75	141,44	323,51	656,22	629,16	866,62	764,23
Boyacá	Gameza	54,57	0,00	0,00	75,97	328,06	255,15	657,50	425,19	267,71	418,76
Boyacá	Garagoa	24,04	5,99	23,90	17,88	249,67	100,86	284,36	337,08	295,09	347,59
Boyacá	Guacamayas	0,00	0,00	102,72	0,00	698,92	606,06	282,33	287,85	59,07	301,75
Boyacá	Guateque	19,86	0,00	0,00	40,34	40,56	418,62	493,52	382,35	551,91	628,14
Boyacá	Guayatá	0,00	0,00	0,00	69,67	320,74	528,23	297,90	419,29	253,61	259,33
Boyacá	Güicán	0,00	0,00	13,32	53,94	164,14	138,66	42,19	57,04	28,95	147,04
Boyacá	Iza	0,00	45,72	0,00	224,22	222,12	87,68	607,38	172,04	425,71	126,42
Boyacá	Jenesano	13,37	13,32	53,10	105,86	237,84	224,07	302,15	341,03	196,34	470,04
Boyacá	Jericó	0,00	0,00	90,09	0,00	93,17	165,76	192,22	122,22	149,63	227,50
Boyacá	La Capilla	0,00	0,00	0,00	70,50	252,25	441,99	263,36	461,01	235,29	320,13
Boyacá	La Uvita	89,05	30,71	31,86	32,93	102,60	141,64	257,45	343,64	554,89	246,41
Boyacá	La Victoria	0,00	0,00	0,00	0,00	238,95	59,74	179,21	119,47	238,95	298,69
Boyacá	Labranzagrande	0,00	56,78	38,02	210,04	115,12	96,41	96,94	77,99	156,89	0,00
Boyacá	Macanal	0,00	0,00	0,00	167,57	208,99	271,06	291,36	394,76	331,88	352,19
Boyacá	Maripí	63,76	0,00	12,89	12,98	52,20	52,57	145,44	132,96	213,90	228,99
Boyacá	Miraflores	20,63	0,00	0,00	10,27	10,27	318,14	338,39	337,94	368,21	388,59
Boyacá	Mongua	0,00	0,00	0,00	40,05	303,89	122,72	248,34	293,26	84,80	171,78

Boyacá	Monguí	0,00	0,00	40,04	0,00	160,26	200,40	300,66	501,20	220,62	521,56
Boyacá	Moniquirá	32,13	0,00	36,84	198,44	513,39	519,31	557,83	573,19	406,50	576,25
Boyacá	Motavita	28,44	0,00	27,46	161,97	159,15	208,58	141,08	327,74	173,55	244,05
Boyacá	Muzo	0,00	0,00	183,77	155,15	251,65	212,34	118,50	207,51	254,42	358,99
Boyacá	Nobsa	38,81	12,84	6,37	82,33	270,42	412,45	466,33	457,38	546,99	513,67
Boyacá	Nuevo Colón	48,52	16,04	0,00	157,65	297,71	233,06	200,93	353,14	335,42	212,06
Boyacá	Oicatá	0,00	0,00	70,70	211,71	423,73	353,23	776,29	353,11	635,14	353,23
Boyacá	Otanche	0,00	0,00	9,47	103,96	179,26	178,96	253,93	244,20	168,86	140,57
Boyacá	Pachavita	0,00	0,00	69,93	356,76	402,64	224,30	342,99	507,61	598,09	285,36
Boyacá	Páez	0,00	0,00	31,41	127,06	290,70	393,06	266,13	168,58	411,95	209,28
Boyacá	Paipa	0,00	17,41	17,23	54,57	141,86	478,42	497,08	640,33	627,85	496,44
Boyacá	Pajarito	88,73	0,00	0,00	49,00	0,00	316,12	380,43	337,84	116,35	239,52
Boyacá	Panqueba	0,00	57,64	58,89	0,00	369,69	251,41	127,96	328,73	538,00	137,74
Boyacá	Pauna	28,67	0,00	0,00	47,10	168,89	168,30	195,77	213,85	167,01	138,93
Boyacá	Paya	0,00	0,00	114,68	0,00	0,00	116,01	233,01	351,15	156,86	39,42
Boyacá	Paz de Río	0,00	19,62	0,00	100,48	630,34	432,28	396,16	486,05	405,98	324,82
Boyacá	Pesca	31,93	0,00	0,00	168,80	378,66	304,34	406,26	426,67	286,35	356,14
Boyacá	Pisba	0,00	0,00	0,00	0,00	281,89	71,68	72,25	146,63	297,62	226,76
Boyacá	Puerto Boyacá	1,94	0,00	3,81	5,66	114,06	194,68	202,24	182,36	204,39	201,10
Boyacá	Quípama	0,00	0,00	0,00	0,00	36,30	85,72	123,85	87,88	139,70	205,76
Boyacá	Ramiriquí	75,15	37,84	295,35	172,70	164,46	273,04	216,15	455,58	409,39	302,24
Boyacá	Ráquira	15,67	0,00	84,69	61,10	151,66	165,60	149,51	237,42	242,86	197,34
Boyacá	Rondón	0,00	0,00	0,00	0,00	34,54	104,24	104,90	105,60	354,36	286,12
Boyacá	Saboyá	15,56	0,00	15,70	63,05	166,26	175,10	104,02	313,56	210,15	308,72
Boyacá	Sáchica	0,00	0,00	52,04	26,08	365,82	471,33	524,93	710,34	633,08	687,83
Boyacá	Samacá	0,00	21,80	10,77	15,96	68,36	93,56	421,31	513,19	442,06	392,72
Boyacá	San Eduardo	0,00	0,00	156,74	52,47	368,03	158,48	265,53	320,86	590,76	321,37

Boyacá	San José de Pare	35,47	0,00	36,05	90,91	458,80	352,18	112,36	340,84	459,68	445,91
Boyacá	San Luis de Gaceno	16,35	0,00	0,00	0,00	106,95	255,52	279,85	248,33	253,91	120,10
Boyacá	San Mateo	0,00	22,55	23,12	190,07	560,02	702,28	359,90	607,82	434,55	502,51
Boyacá	San Miguel de Sema	0,00	0,00	65,30	65,37	65,46	131,09	218,82	175,32	263,39	395,78
Boyacá	San Pablo de Borbur	0,00	0,00	0,00	83,73	112,07	150,05	75,31	37,86	38,01	76,35
Boyacá	Santa María	0,00	0,00	0,00	69,69	212,41	359,88	97,44	247,40	226,13	944,36
Boyacá	Santa Rosa de Viterbo	15,02	0,00	89,80	22,42	358,64	321,09	209,06	335,85	462,58	328,19
Boyacá	Santa Sofía	0,00	0,00	0,00	206,26	243,73	777,93	286,74	546,45	147,93	338,60
Boyacá	Santana	0,00	12,80	64,12	12,85	373,42	438,77	672,62	427,90	520,02	365,01
Boyacá	Sativanorte	0,00	0,00	0,00	0,00	397,93	365,41	124,07	251,05	384,78	260,19
Boyacá	Sativasur	0,00	0,00	0,00	325,20	334,45	422,30	173,01	264,78	270,27	91,41
Boyacá	Siachoque	22,44	11,21	0,00	11,19	178,89	89,39	323,81	357,14	290,05	200,74
Boyacá	Soatá	33,83	0,00	47,41	230,55	572,78	510,59	484,42	510,34	634,05	707,31
Boyacá	Socha	79,95	0,00	40,37	27,09	286,03	274,09	386,69	319,71	70,03	366,61
Boyacá	Socotá	0,00	0,00	0,00	10,88	89,33	239,81	210,75	96,00	98,43	327,70
Boyacá	Sogamoso	3,43	103,19	70,73	281,23	352,63	499,67	478,93	505,46	504,88	614,42
Boyacá	Somondoco	0,00	0,00	0,00	0,00	0,00	233,28	371,75	567,11	247,80	815,75
Boyacá	Sora	0,00	0,00	0,00	133,02	132,85	99,47	198,74	132,36	363,64	396,43
Boyacá	Soracá	0,00	34,67	17,50	17,73	178,89	343,64	146,36	593,14	579,11	492,52
Boyacá	Sotaquirá	11,47	11,63	0,00	0,00	133,80	259,58	163,23	306,40	168,63	184,75
Boyacá	Susacón	0,00	0,00	116,11	206,49	300,03	398,04	217,80	349,87	96,93	132,14
Boyacá	Sutamarchán	0,00	0,00	0,00	49,73	232,87	217,03	318,47	302,93	185,94	373,51
Boyacá	Sutatenza	0,00	0,00	45,81	46,20	23,42	236,80	359,28	411,92	220,26	396,73
Boyacá	Tasco	0,00	14,78	29,81	195,28	302,98	244,57	216,12	389,23	314,42	412,96
Boyacá	Tenza	0,00	0,00	0,00	45,49	299,40	373,31	236,52	550,50	316,15	394,87
Boyacá	Tibaná	20,78	0,00	231,09	211,35	329,47	406,16	236,69	291,96	555,19	372,15
Boyacá	Tibasosa	38,64	7,64	0,00	14,95	118,26	322,06	420,44	365,96	284,43	415,61

Boyacá	Tinjacá	0,00	0,00	33,44	166,67	265,87	397,75	462,96	396,04	461,29	526,49
Boyacá	Tipacoque	0,00	0,00	0,00	56,66	202,20	234,67	508,98	335,06	374,30	348,10
Boyacá	Toca	9,52	0,00	9,59	0,00	28,99	29,13	146,20	147,02	226,44	277,06
Boyacá	Togüí	0,00	0,00	0,00	58,64	275,10	335,90	159,05	500,10	362,46	202,80
Boyacá	Tópaga	0,00	27,11	0,00	108,34	135,39	406,06	135,35	351,92	324,85	730,91
Boyacá	Tota	0,00	0,00	17,92	36,03	253,58	382,51	293,20	202,88	278,50	317,88
Boyacá	Tunja	2,48	10,34	46,49	111,61	149,52	218,57	298,21	338,09	382,74	438,72
Boyacá	Tununguá	59,95	59,21	0,00	0,00	0,00	282,33	55,68	384,62	271,74	107,47
Boyacá	Turmequé	0,00	0,00	0,00	29,07	118,68	333,43	216,92	427,08	420,58	297,52
Boyacá	Tuta	0,00	21,71	10,78	42,77	116,83	158,21	251,23	291,21	268,79	226,01
Boyacá	Tutazá	0,00	0,00	0,00	48,45	196,75	200,30	254,58	416,23	423,28	323,62
Boyacá	Umbita	39,29	0,00	19,53	97,42	165,31	213,63	194,02	242,41	252,08	164,70
Boyacá	Ventaquemada	0,00	0,00	6,73	26,72	192,54	244,02	275,25	254,09	278,46	270,41
Boyacá	Villa de Leyva	23,37	0,00	0,00	113,27	665,39	412,59	896,66	550,58	631,14	989,17
Boyacá	Viracachá	0,00	0,00	29,54	267,54	360,69	363,86	396,58	430,64	372,44	374,88
Boyacá	Zetaquira	0,00	0,00	0,00	20,62	41,77	338,84	341,88	346,77	307,22	311,87
Caldas	Aguadas	193,00	194,86	34,21	4,32	17,43	233,19	271,07	183,91	240,03	205,81
Caldas	Anserma	134,88	141,16	98,35	69,68	104,93	204,83	220,24	265,33	245,62	139,59
Caldas	Aranzazu	270,96	209,80	16,32	16,51	83,54	228,00	299,20	354,49	411,49	407,33
Caldas	Belalcázar	222,66	397,55	270,13	202,34	435,01	349,43	542,30	465,33	506,31	390,33
Caldas	Chinchiná	145,11	393,37	362,53	240,05	409,20	403,10	427,74	384,94	452,50	442,67
Caldas	Filadelfia	209,61	155,47	66,40	25,27	153,79	78,01	219,99	321,43	271,89	229,91
Caldas	La Dorada	16,26	39,08	48,25	16,00	94,15	141,15	183,76	169,77	218,29	281,82
Caldas	La Merced	369,97	204,63	128,33	294,74	184,07	221,84	226,52	177,84	236,02	185,43
Caldas	Manizales	140,57	206,92	180,69	193,83	218,93	224,19	280,29	323,09	321,66	325,56
Caldas	Manzanares	76,84	126,33	57,48	53,79	158,43	151,23	245,51	183,39	4,30	298,55
Caldas	Marmato	174,48	265,44	45,84	170,80	158,23	134,74	256,47	188,34	87,95	251,04

Caldas	Marquetalia	188,50	221,80	33,55	20,11	33,47	167,16	180,36	253,64	213,45	293,33
Caldas	Marulanda	0,00	0,00	57,95	29,04	58,16	87,49	0,00	29,33	117,44	29,45
Caldas	Neira	174,51	131,54	30,90	68,12	111,53	107,28	99,83	181,61	117,98	240,73
Caldas	Norcasia	191,18	207,41	223,91	90,36	258,01	275,65	385,74	295,49	345,15	411,26
Caldas	Pácora	117,33	304,27	210,27	274,64	235,63	373,74	366,83	392,03	435,07	376,94
Caldas	Palestina	383,16	228,04	16,71	256,68	413,64	442,21	370,08	606,91	388,51	507,99
Caldas	Pensilvania	170,34	162,82	45,45	11,37	34,11	238,82	204,78	128,98	140,36	269,52
Caldas	Riosucio	159,38	207,00	164,28	65,59	148,40	146,61	176,49	212,18	297,39	274,50
Caldas	Risaralda	411,25	338,20	175,85	69,12	89,76	50,43	71,43	154,75	177,40	475,13
Caldas	Salamina	149,09	162,52	101,58	141,73	238,98	345,84	312,27	359,50	432,82	465,80
Caldas	Samaná	109,02	252,93	31,11	89,40	283,62	132,04	186,34	159,11	209,49	166,77
Caldas	San José	224,36	171,57	145,12	171,39	171,53	144,93	329,64	276,50	263,57	302,83
Caldas	Supía	35,67	137,68	62,48	38,78	19,24	38,21	22,76	48,98	389,11	260,13
Caldas	Victoria	453,44	300,84	44,92	215,13	479,73	288,18	337,52	529,10	606,06	636,94
Caldas	Villamaría	122,38	152,54	159,58	162,36	149,65	238,98	247,34	387,48	277,07	355,51
Caldas	Viterbo	316,24	364,94	62,50	94,29	86,88	126,95	239,02	95,95	88,22	297,48
Caquetá	Albania	31,24	31,19	0,00	46,71	15,57	15,58	0,00	31,11	124,42	171,02
Caquetá	Belén de los Andaquies	8,95	35,68	0,00	26,59	149,99	79,08	166,26	95,83	77,98	146,54
Caquetá	Cartagena del Chairá	6,76	0,00	13,12	0,00	22,28	43,88	67,93	82,11	65,89	132,71
Caquetá	Curillo	26,71	35,41	17,62	26,32	113,44	95,51	95,04	77,37	188,31	178,92
Caquetá	El Doncello	46,12	4,60	9,17	9,14	59,28	122,81	181,50	208,25	176,18	103,68
Caquetá	El Paujil	60,83	5,46	0,00	53,10	83,81	160,08	193,46	175,60	163,17	194,86
Caquetá	Florencia	35,55	45,45	43,98	32,38	61,72	186,10	211,01	257,48	269,78	279,35
Caquetá	La Montañita	0,00	17,73	13,21	13,13	13,05	8,64	4,29	63,95	67,74	96,68
Caquetá	Milán	43,31	51,88	0,00	0,00	34,38	51,46	25,66	42,67	34,06	25,48
Caquetá	Morelia	0,00	0,00	0,00	0,00	0,00	26,62	132,52	158,27	288,49	208,55
Caquetá	Puerto Rico	6,13	12,24	24,41	27,38	15,16	18,14	18,09	27,06	17,99	128,56

Caquetá	San José del Fragua	21,29	7,04	20,98	76,39	130,99	136,88	129,15	87,74	227,87	226,23
Caquetá	San Vicente del Caguán	3,40	11,69	8,20	8,05	26,88	71,42	120,45	148,23	158,84	167,60
Caquetá	Solano	19,78	0,00	4,76	13,99	22,86	26,88	21,97	34,47	12,68	49,73
Caquetá	Solita	32,82	54,68	65,65	65,58	153,01	54,65	54,69	10,93	164,11	76,56
Caquetá	Valparaíso	35,68	53,27	0,00	8,80	35,03	113,31	86,78	103,65	34,40	51,34
Casanare	Aguazul	152,25	64,12	21,75	253,08	447,13	419,66	448,60	376,79	423,05	327,75
Casanare	Chameza	50,81	49,04	0,00	46,04	44,76	0,00	85,03	332,23	203,25	199,12
Casanare	Hato Corozal	104,79	112,16	64,26	99,02	141,59	217,18	281,59	276,61	65,86	218,38
Casanare	La Salina	150,72	0,00	0,00	0,00	73,05	72,41	215,21	0,00	70,42	139,47
Casanare	Maní	143,18	340,44	35,86	44,81	152,41	412,52	430,80	565,58	457,85	377,36
Casanare	Monterrey	219,90	29,87	110,21	180,88	185,26	428,40	339,43	375,84	465,24	386,13
Casanare	Nunchía	246,86	257,58	0,00	255,01	184,50	827,21	492,10	409,60	317,21	248,11
Casanare	Orocué	343,03	88,35	237,83	49,73	407,31	441,34	207,14	169,47	132,39	335,41
Casanare	Paz de Ariporo	40,64	70,34	144,62	59,45	223,42	354,49	250,55	359,93	326,99	418,33
Casanare	Pore	75,39	75,51	302,15	327,50	592,61	289,93	479,74	479,80	492,36	606,44
Casanare	Recetor	34,58	0,00	0,00	0,00	57,59	0,00	0,00	0,00	0,00	402,65
Casanare	Sabanalarga	119,80	333,64	122,74	218,14	378,91	512,66	292,87	428,19	200,87	373,77
Casanare	Sácama	0,00	0,00	54,67	53,71	634,25	313,15	411,52	455,93	99,95	147,49
Casanare	San Luis de Palenque	628,85	239,87	132,73	66,04	328,60	235,42	247,14	271,77	270,37	243,28
Casanare	Támara	211,24	127,01	28,26	14,15	28,32	368,59	184,37	298,04	369,11	326,89
Casanare	Tauramena	46,14	33,52	48,76	215,51	275,31	346,28	360,06	438,39	371,44	426,82
Casanare	Trinidad	305,96	209,63	141,43	7,66	89,71	233,46	249,18	264,11	271,20	271,43
Casanare	Villanueva	283,67	330,40	70,79	74,54	308,53	434,86	413,95	524,49	377,22	415,32
Casanare	Yopal	104,88	117,32	82,45	59,17	213,96	316,29	307,01	265,23	259,78	280,46
Cauca	Almaguer	9,68	4,82	81,52	219,74	195,15	90,15	170,30	188,73	188,30	192,61
Cauca	Argelia	0,00	35,89	130,49	82,33	120,47	100,07	118,23	128,43	104,81	211,30
Cauca	Balboa	41,69	91,07	123,20	4,08	100,99	216,29	222,45	193,06	207,12	193,77

Cauca	Bolívar	0,00	38,59	38,54	76,96	135,53	171,36	150,75	240,29	342,96	243,85
Cauca	Buenos Aires	0,00	10,57	6,93	78,25	133,61	147,57	273,65	287,57	251,36	213,19
Cauca	Cajibío	2,84	8,46	5,60	108,37	107,43	133,89	238,42	300,93	349,13	361,97
Cauca	Caldono	0,00	9,53	9,47	53,31	71,62	129,77	297,43	167,36	238,51	257,58
Cauca	Caloto	0,00	177,94	68,80	234,73	405,74	513,23	648,91	823,54	578,17	469,46
Cauca	Corinto	0,00	61,50	97,95	193,62	253,97	254,03	340,95	416,07	244,73	182,69
Cauca	El Tambo	19,49	53,92	38,67	44,95	132,23	144,52	175,79	166,77	286,17	243,32
Cauca	Florencia	0,00	148,69	263,90	345,79	49,31	196,88	654,99	719,07	603,39	748,45
Cauca	Guachené	0,00	0,00	35,73	35,68	61,06	116,77	385,16	364,10	348,22	382,70
Cauca	Guapi	0,00	30,99	78,89	27,34	68,11	115,40	186,09	151,82	215,33	171,16
Cauca	Inzá	0,00	82,17	123,47	132,35	68,72	88,10	260,63	306,32	249,98	252,78
Cauca	Jambaló	0,00	13,01	44,70	94,07	73,81	108,60	130,17	272,69	204,66	117,06
Cauca	La Sierra	0,00	157,51	111,45	428,11	344,86	326,89	458,72	431,44	601,33	498,78
Cauca	La Vega	2,49	34,19	79,11	235,60	152,99	178,09	168,83	206,62	162,41	160,59
Cauca	López	0,00	0,00	10,18	10,13	25,18	80,16	39,85	59,43	9,84	34,24
Cauca	Mercaderes	0,00	16,86	56,09	145,56	111,74	195,13	328,20	294,15	271,30	414,25
Cauca	Miranda	2,91	48,60	190,99	229,05	289,97	242,07	302,98	325,62	266,88	237,30
Cauca	Morales	4,06	4,04	28,10	59,85	186,28	137,76	257,92	217,21	261,91	278,98
Cauca	Padilla	0,00	24,43	380,74	222,33	496,34	411,99	753,20	933,87	482,11	561,44
Cauca	Paez	0,00	36,80	72,95	84,30	32,79	88,49	160,45	219,24	116,95	163,56
Cauca	Patía	11,85	155,82	306,25	179,30	280,82	278,28	295,33	473,84	301,06	336,58
Cauca	Piamonte	0,00	13,99	0,00	83,51	41,64	124,29	151,24	287,55	163,33	108,42
Cauca	Piendamó	0,00	84,82	250,04	184,20	356,64	401,89	508,22	368,15	443,04	514,71
Cauca	Popayán	19,60	45,05	72,40	179,81	241,39	284,83	404,46	471,42	528,93	604,17
Cauca	Puerto Tejada	0,00	129,78	183,02	126,85	237,30	377,98	433,97	346,94	350,28	220,33
Cauca	Puracé	13,28	6,62	158,60	118,71	177,75	124,89	479,22	629,55	648,71	504,29
Cauca	Rosas	0,00	195,76	226,00	511,55	362,07	528,41	509,85	643,01	548,79	627,52

Cauca	San Sebastián	0,00	7,63	83,21	75,02	37,21	258,13	314,14	391,11	258,55	341,54
Cauca	Santa Rosa	0,00	30,58	90,99	10,02	49,62	78,57	165,31	144,51	47,71	47,23
Cauca	Santander de Quilichao	119,71	477,73	119,72	182,65	507,56	556,76	587,77	653,54	602,92	542,92
Cauca	Silvia	0,00	41,59	89,31	184,35	227,85	258,41	326,29	218,61	289,19	290,89
Cauca	Sotara	0,00	18,75	99,16	172,16	97,58	199,42	179,82	225,73	218,06	216,07
Cauca	Suárez	0,00	0,00	116,00	137,56	466,60	377,48	410,58	384,72	310,89	397,64
Cauca	Sucre	89,69	359,15	190,95	202,22	179,67	472,55	382,32	494,77	315,10	236,19
Cauca	Timbío	9,78	209,37	407,33	273,43	530,78	600,71	562,85	684,26	637,49	705,09
Cauca	Timbiquí	0,00	4,76	4,75	28,43	28,32	37,59	84,18	65,15	9,25	32,20
Cauca	Toribio	0,00	150,97	69,32	238,51	185,99	276,08	301,11	429,48	390,58	366,15
Cauca	Totoró	11,17	11,01	16,29	117,71	137,13	83,13	245,79	353,16	407,49	450,56
Cauca	Villa Rica	6,81	424,16	259,45	302,33	538,51	621,64	550,70	593,94	475,63	426,91
Cesar	Aguachica	33,13	11,68	12,68	1,14	12,38	77,83	129,72	151,12	76,38	117,12
Cesar	Agustín Codazzi	1,87	13,17	3,78	0,00	30,63	80,91	182,29	166,03	143,62	148,66
Cesar	Astrea	0,00	5,36	0,00	0,00	0,00	0,00	78,70	250,91	250,07	145,42
Cesar	Becerril	0,00	0,00	0,00	14,56	109,65	80,76	339,01	444,18	341,93	380,94
Cesar	Bosconia	3,11	0,00	0,00	2,93	0,00	2,83	5,56	10,92	2,68	92,42
Cesar	Chimichagua	0,00	0,00	9,70	6,47	84,21	175,15	175,43	123,70	159,83	176,56
Cesar	Chiriguaná	9,23	18,65	0,00	4,77	96,66	166,35	138,76	135,56	203,56	196,13
Cesar	Curumaní	0,00	0,00	0,00	57,67	202,48	303,77	351,69	255,12	209,30	153,94
Cesar	El Copey	0,00	7,84	11,69	34,86	7,71	314,31	396,58	280,79	339,97	327,23
Cesar	El Paso	4,70	9,31	9,22	4,57	4,53	0,00	8,91	269,28	21,90	78,22
Cesar	Gamarra	0,00	13,24	0,00	0,00	38,03	43,77	80,19	79,09	12,02	17,80
Cesar	González	0,00	94,04	0,00	0,00	0,00	65,61	148,47	180,61	243,20	220,95
Cesar	La Gloria	0,00	0,00	0,00	0,00	44,08	81,80	195,89	244,11	216,42	164,58
Cesar	La Jagua de Ibirico	9,05	0,00	0,00	58,66	252,43	229,67	319,39	305,54	242,35	188,25
Cesar	La Paz	0,00	31,48	0,00	0,00	4,44	8,84	101,42	184,61	210,39	139,82

Cesar	Manaure	0,00	0,00	7,96	23,30	37,88	88,74	187,75	317,17	172,25	262,54
Cesar	Pailitas	0,00	0,00	6,07	0,00	5,98	403,94	365,87	328,31	326,23	335,88
Cesar	Pelaya	0,00	0,00	0,00	0,00	51,72	222,45	334,11	382,11	139,59	266,34
Cesar	Pueblo Bello	0,00	0,00	0,00	0,00	24,81	14,51	70,77	133,45	85,30	201,43
Cesar	Río de Oro	13,94	13,97	14,00	112,32	119,65	134,10	198,19	248,49	277,76	171,50
Cesar	San Alberto	38,26	0,00	0,00	13,46	188,95	150,71	156,12	173,74	101,41	91,56
Cesar	San Diego	0,00	0,00	0,00	95,55	36,86	81,35	155,84	141,51	261,66	187,66
Cesar	San Martín	28,42	16,93	16,81	61,18	359,33	54,91	92,78	292,84	161,74	58,98
Cesar	Tamalameque	0,00	28,53	0,00	21,45	21,47	50,18	100,52	172,71	173,14	202,34
Cesar	Valledupar	36,09	1,56	2,54	5,95	8,23	56,46	65,32	75,81	74,14	92,18
Choco	Riosucio	0,00	0,00	7,01	3,50	3,49	0,00	0,00	0,00	0,00	0,00
Chocó	Acandí	0,00	0,00	0,00	19,97	30,24	10,16	20,50	0,00	0,00	0,00
Chocó	Alto Baudo	0,00	51,52	31,47	0,00	3,00	2,93	0,00	5,58	0,00	0,00
Chocó	Atrato	0,00	0,00	0,00	0,00	0,00	0,00	42,54	0,00	0,00	29,43
Chocó	Bagadó	0,00	0,00	0,00	0,00	0,00	0,00	12,28	0,00	37,20	0,00
Chocó	Bahía Solano	0,00	0,00	32,66	10,86	0,00	0,00	0,00	21,50	42,89	53,47
Chocó	Bajo Baudó	0,00	0,00	297,94	5,92	0,00	0,00	0,00	23,13	5,75	0,00
Chocó	Belén de Bajira	0,00	0,00	0,00	0,00	0,00	0,00	0,00	0,00	0,00	0,00
Chocó	Bojaya	0,00	0,00	199,94	0,00	0,00	0,00	0,00	0,00	0,00	19,79
Chocó	Carmen del Darien	0,00	0,00	0,00	0,00	0,00	0,00	0,00	18,41	18,31	72,83
Chocó	Cértegui	0,00	0,00	0,00	0,00	0,00	10,09	0,00	0,00	0,00	0,00
Chocó	Condoto	14,97	44,42	80,45	36,13	42,85	0,00	48,86	41,41	0,00	6,75
Chocó	El Cantón del San Pablo	0,00	0,00	0,00	0,00	0,00	67,60	0,00	0,00	0,00	0,00
Chocó	El Carmen de Atrato	0,00	80,19	0,00	38,73	38,10	44,94	51,53	57,89	7,12	55,98
Chocó	El Litoral del San Juan	0,00	0,00	14,92	0,00	0,00	0,00	0,00	13,42	0,00	0,00
Chocó	Istmina	0,00	0,00	0,00	4,09	4,06	4,03	4,00	3,97	0,00	0,00
Chocó	Juradó	0,00	0,00	0,00	0,00	0,00	146,76	0,00	0,00	0,00	0,00

Chocó	Lloró	0,00	0,00	9,40	9,32	0,00	0,00	0,00	0,00	0,00	0,00
Chocó	Medio Atrato	0,00	0,00	0,00	0,00	0,00	0,00	3,62	3,50	0,00	0,00
Chocó	Medio Baudó	0,00	0,00	88,45	0,00	0,00	0,00	0,00	0,00	0,00	0,00
Chocó	Medio San Juan	0,00	71,83	91,32	20,60	13,43	39,38	6,41	0,00	0,00	0,00
Chocó	Nóvita	0,00	0,00	12,66	0,00	25,25	0,00	25,19	0,00	0,00	0,00
Chocó	Nuquí	0,00	0,00	262,63	12,36	0,00	12,08	23,88	11,79	23,32	57,68
Chocó	Quibdó	0,00	6,15	2,63	8,73	5,23	8,69	36,43	45,88	12,10	28,47
Chocó	Río Iro	0,00	0,00	34,48	0,00	0,00	0,00	0,00	0,00	0,00	0,00
Chocó	Río Quito	0,00	12,21	24,08	0,00	23,48	0,00	0,00	11,30	0,00	0,00
Chocó	San José del Palmar	0,00	0,00	20,12	60,62	142,07	0,00	82,05	102,99	0,00	0,00
Chocó	Sipí	0,00	0,00	0,00	0,00	0,00	0,00	0,00	0,00	0,00	0,00
Chocó	Tadó	0,00	0,00	0,00	5,40	0,00	0,00	32,00	0,00	0,00	0,00
Chocó	Unguía	0,00	0,00	0,00	0,00	0,00	13,36	6,66	6,63	0,00	0,00
Chocó	Unión Panamericana	0,00	0,00	0,00	0,00	22,23	0,00	0,00	0,00	0,00	0,00
Córdoba	Ayapel	0,00	0,00	2,19	6,45	21,09	49,66	79,19	151,39	115,32	69,02
Córdoba	Buenavista	0,00	30,40	150,07	98,72	97,43	62,47	75,89	98,30	60,11	164,27
Córdoba	Canalete	0,00	10,85	26,55	51,96	71,14	124,19	63,13	71,23	88,18	186,06
Córdoba	Cereté	2,34	15,09	69,04	95,80	84,78	146,79	176,62	139,89	255,67	212,34
Córdoba	Chimá	0,00	0,00	0,00	13,96	20,75	13,70	115,32	40,31	19,98	26,38
Córdoba	Chinú	2,26	6,72	70,86	146,64	82,23	128,37	196,76	171,58	225,65	219,08
Córdoba	Ciénaga de Oro	0,00	24,90	122,19	71,93	199,93	125,26	176,24	212,59	132,35	120,66
Córdoba	Cotorra	6,60	72,40	164,23	131,04	372,52	201,95	136,54	123,26	12,95	51,66
Córdoba	La Apartada	0,00	14,93	0,00	0,00	0,00	13,89	61,37	60,27	85,50	32,30
Córdoba	Lorica	42,94	73,75	146,46	121,77	150,47	130,39	163,76	138,80	166,61	241,05
Córdoba	Los Córdobas	5,30	20,61	10,02	121,72	118,27	114,85	107,15	99,71	130,47	102,16
Córdoba	Momil	0,00	7,07	105,37	6,98	48,50	337,00	375,58	250,81	302,74	287,39
Córdoba	Moñitos	0,00	0,00	0,00	3,94	7,76	38,18	63,93	51,83	61,97	17,95

Córdoba	Montelíbano	0,00	2,89	8,46	4,13	40,39	52,62	123,44	116,92	135,23	212,79
Córdoba	Montería	5,63	35,25	46,86	59,09	142,36	174,80	168,46	186,00	187,40	183,84
Córdoba	Planeta Rica	0,00	20,55	7,84	1,55	26,18	12,21	10,59	21,01	49,12	63,46
Córdoba	Pueblo Nuevo	0,00	0,00	23,43	54,55	61,86	112,99	94,51	58,21	31,12	27,97
Córdoba	Puerto Escondido	8,68	12,65	12,29	75,64	69,55	90,05	69,19	42,41	41,18	43,31
Córdoba	Puerto Libertador	0,00	5,25	0,00	7,39	62,02	30,03	60,41	125,68	128,04	89,47
Córdoba	Purísima	0,00	0,00	13,50	40,40	93,89	66,90	206,82	186,33	145,96	231,57
Córdoba	Sahagún	0,00	57,81	163,91	144,31	191,11	258,96	280,64	227,52	219,21	196,46
Córdoba	San Andrés Sotavento	42,36	64,62	38,33	122,69	140,27	141,66	128,14	158,44	119,31	59,26
Córdoba	San Antero	0,00	116,11	131,79	125,91	284,89	272,89	178,57	139,62	121,15	147,14
Córdoba	San Bernardo del Viento	0,00	43,34	144,05	127,40	198,04	151,39	152,72	183,04	207,00	187,71
Córdoba	San Carlos	0,00	0,00	8,02	0,00	77,99	92,30	121,37	123,42	243,51	127,34
Córdoba	San José de Uré	0,00	0,00	0,00	0,00	0,00	0,00	18,75	83,16	9,10	35,80
Córdoba	San Pelayo	0,00	4,94	29,35	29,04	16,76	18,95	37,49	64,92	29,83	29,50
Córdoba	Tierralta	0,00	34,32	110,97	79,02	77,15	52,72	38,85	20,50	19,02	68,39
Córdoba	Tuchín	0,00	0,00	27,30	47,50	31,94	76,66	108,26	119,37	92,80	111,38
Córdoba	Valencia	0,00	0,00	13,32	70,34	94,25	146,91	102,23	190,43	118,68	202,48
Cundinamarca	Agua de Dios	0,00	69,16	287,06	341,54	458,63	284,24	465,45	306,78	263,76	733,27
Cundinamarca	Albán	16,80	100,79	134,36	67,18	117,55	386,23	100,76	369,38	235,06	33,58
Cundinamarca	Anapoima	8,46	91,67	98,55	97,13	446,61	306,34	309,89	419,66	307,99	510,58
Cundinamarca	Anolaima	7,66	154,24	209,69	383,26	204,90	452,42	344,22	451,36	324,91	417,69
Cundinamarca	Apulo	0,00	51,17	115,15	435,06	588,69	652,76	652,76	614,44	460,83	448,03
Cundinamarca	Arbeláez	16,80	175,67	116,65	124,43	140,41	361,99	336,04	473,59	154,57	340,63
Cundinamarca	Beltrán	0,00	0,00	0,00	0,00	191,48	142,18	93,68	46,15	91,03	0,00
Cundinamarca	Bituima	0,00	38,23	38,52	77,31	77,40	234,47	195,77	157,67	277,78	119,62
Cundinamarca	Bojacá	31,94	103,59	151,39	236,03	201,28	168,10	364,43	319,89	441,37	506,54
Cundinamarca	Cabrera	0,00	0,00	324,96	239,23	349,42	219,30	418,50	530,97	577,91	334,97

Cundinamarca	Cachipay	10,03	0,00	10,06	110,81	100,91	192,07	273,47	192,82	325,43	407,71
Cundinamarca	Cajicá	6,29	86,04	234,19	168,30	212,46	434,48	430,80	418,25	374,51	491,07
Cundinamarca	Caparrapí	0,00	0,00	6,03	60,24	42,13	84,15	48,05	137,93	59,91	83,78
Cundinamarca	Caqueza	0,00	156,78	198,33	149,72	340,08	344,68	437,95	536,21	428,20	455,37
Cundinamarca	Carmen de Carupa	0,00	127,34	184,03	205,62	56,70	168,82	212,15	199,31	109,78	174,08
Cundinamarca	Chaguaní	0,00	24,65	74,33	149,22	74,70	175,04	150,04	426,71	251,19	150,79
Cundinamarca	Chía	10,62	121,29	146,57	207,15	117,51	174,04	192,18	302,41	334,79	366,37
Cundinamarca	Chipaque	0,00	95,27	95,26	190,52	142,87	369,09	273,84	607,14	523,81	607,14
Cundinamarca	Choachí	18,03	126,66	263,21	273,47	164,79	358,09	323,30	435,95	344,86	449,44
Cundinamarca	Chocontá	4,86	0,00	60,02	139,50	57,01	76,92	129,17	178,72	372,17	266,46
Cundinamarca	Cogua	0,00	35,95	130,87	138,11	188,57	189,59	148,72	145,91	237,02	324,76
Cundinamarca	Cota	28,74	135,66	164,59	272,67	270,99	483,22	569,11	602,31	678,28	802,14
Cundinamarca	Cucunubá	0,00	97,85	152,78	151,56	315,03	136,04	365,01	322,88	334,27	372,29
Cundinamarca	El Colegio	0,00	138,80	57,03	84,92	178,17	358,81	314,93	465,16	696,23	747,19
Cundinamarca	El Peñón	0,00	20,33	0,00	81,93	20,57	247,63	351,53	228,31	145,68	353,65
Cundinamarca	El Rosal	14,03	75,25	186,84	280,00	292,42	409,38	376,08	580,71	550,60	708,30
Cundinamarca	Facatativá	17,78	118,32	224,03	246,98	175,77	324,57	321,48	348,57	341,39	331,54
Cundinamarca	Fomeque	8,26	173,24	263,50	164,41	246,43	484,44	385,25	483,37	368,43	547,65
Cundinamarca	Fosca	0,00	58,15	114,93	70,96	98,11	166,04	150,21	94,31	199,36	262,12
Cundinamarca	Funza	3,11	59,41	79,07	96,50	97,44	290,84	299,04	278,52	321,17	394,83
Cundinamarca	Fúquene	37,90	188,25	112,17	37,12	239,45	219,26	126,83	143,70	373,87	476,27
Cundinamarca	Fusagasugá	14,93	175,26	158,04	144,81	154,70	261,27	306,26	287,31	252,74	239,13
Cundinamarca	Gachala	17,04	0,00	68,68	258,49	190,18	381,61	313,15	244,29	209,97	52,62
Cundinamarca	Gachancipá	0,00	42,03	73,52	190,72	309,02	210,34	190,09	405,46	498,55	445,01
Cundinamarca	Gachetá	0,00	28,33	243,93	121,12	259,14	128,74	283,52	399,38	342,77	277,70
Cundinamarca	Gama	25,69	76,86	204,39	0,00	203,10	202,43	151,29	100,50	350,35	274,25
Cundinamarca	Girardot	4,02	156,55	220,70	205,32	320,02	315,00	286,98	324,48	320,69	351,94

Cundinamarca	Granada	0,00	0,00	290,62	193,62	365,61	246,46	324,75	388,33	472,08	416,81
Cundinamarca	Guachetá	17,41	26,14	340,23	305,68	393,46	393,91	490,75	342,17	500,66	413,30
Cundinamarca	Guaduas	12,09	192,78	145,51	168,48	128,96	217,43	213,50	180,43	195,49	240,64
Cundinamarca	Guasca	178,74	190,83	262,51	339,08	572,05	512,67	518,10	557,85	589,47	566,55
Cundinamarca	Guataquí	0,00	79,30	276,13	196,16	234,10	116,32	77,07	153,14	228,14	37,75
Cundinamarca	Guatavita	14,85	59,20	191,91	235,68	322,63	336,50	175,00	247,31	376,92	375,51
Cundinamarca	Guayabal de Siquima	0,00	110,04	247,87	303,11	385,78	303,11	55,08	165,11	384,83	246,98
Cundinamarca	Guayabetal	0,00	228,64	413,48	123,61	513,98	594,26	408,00	548,67	365,04	282,31
Cundinamarca	Gutiérrez	27,86	0,00	80,97	371,55	260,69	256,94	126,17	173,40	244,08	215,62
Cundinamarca	Jerusalén	0,00	0,00	111,28	259,36	445,93	483,45	409,23	223,63	410,60	74,54
Cundinamarca	Junín	35,68	166,15	200,90	94,24	211,39	328,06	23,35	116,43	162,60	266,08
Cundinamarca	La Calera	48,87	164,37	201,43	175,18	95,87	325,15	615,44	754,54	773,79	713,82
Cundinamarca	La Mesa	28,68	208,54	181,12	257,39	236,76	286,70	289,08	391,73	417,86	468,21
Cundinamarca	La Palma	29,80	29,58	68,48	29,11	19,25	76,39	85,28	319,46	111,87	267,92
Cundinamarca	La Peña	0,00	128,59	185,66	214,10	99,86	228,08	313,43	270,50	256,08	127,93
Cundinamarca	La Vega	0,00	58,98	263,56	181,61	86,62	329,68	441,22	275,72	295,15	572,39
Cundinamarca	Lenguazaque	0,00	30,25	150,59	9,99	129,19	69,18	167,17	293,63	194,78	116,45
Cundinamarca	Macheta	0,00	164,97	150,94	76,08	214,76	216,52	249,45	125,71	237,49	302,93
Cundinamarca	Madrid	4,58	120,82	230,51	224,14	95,02	295,54	371,31	295,62	316,90	350,10
Cundinamarca	Manta	0,00	43,24	517,02	172,15	85,95	299,40	234,79	255,48	381,44	549,45
Cundinamarca	Medina	0,00	50,34	150,68	20,05	10,00	239,43	159,08	198,43	217,65	167,75
Cundinamarca	Mosquera	5,97	59,51	69,22	137,55	160,74	135,68	233,92	282,57	309,37	398,39
Cundinamarca	Nariño	0,00	188,77	328,79	420,56	697,35	693,48	367,65	182,73	363,14	180,34
Cundinamarca	Nemocón	0,00	8,36	172,50	129,11	206,14	280,55	183,91	308,99	318,80	335,59
Cundinamarca	Nilo	0,00	19,57	88,88	191,68	132,51	176,04	217,28	167,37	195,82	95,46
Cundinamarca	Nimaima	0,00	51,35	185,03	82,47	178,08	31,75	31,12	30,55	29,94	29,37
Cundinamarca	Nocaima	0,00	0,00	38,51	76,69	50,92	215,52	88,18	376,55	112,44	285,54

Cundinamarca	Pacho	11,68	7,73	7,68	19,07	41,66	240,60	373,33	318,67	312,74	379,73
Cundinamarca	Paime	0,00	0,00	0,00	0,00	0,00	0,00	64,25	261,49	155,49	22,69
Cundinamarca	Pandi	0,00	181,42	288,81	342,22	286,53	196,60	320,57	142,02	300,46	405,21
Cundinamarca	Paratebueno	0,00	66,53	198,75	171,55	223,42	431,99	352,07	246,82	375,36	386,90
Cundinamarca	Pasca	0,00	78,67	69,27	120,14	119,05	176,92	133,57	215,36	238,19	252,63
Cundinamarca	Puerto Salgar	18,57	18,22	53,67	35,12	143,74	146,65	260,53	223,23	203,34	273,55
Cundinamarca	Pulí	0,00	0,00	0,00	304,36	101,21	168,92	201,68	436,10	33,34	99,77
Cundinamarca	Quebradanegra	0,00	85,03	42,47	276,13	42,35	127,09	148,15	211,28	126,64	168,60
Cundinamarca	Quetame	14,96	74,22	132,33	233,17	144,53	831,66	99,56	155,15	364,09	194,63
Cundinamarca	Quipile	12,23	170,94	12,23	85,51	207,98	232,44	183,58	220,56	171,48	171,40
Cundinamarca	Ricaurte	0,00	46,95	185,08	330,64	415,36	563,91	447,16	365,04	402,50	511,54
Cundinamarca	San Antonio del Tequendama	7,99	7,94	236,89	117,79	62,43	372,58	447,91	422,43	374,50	479,16
Cundinamarca	San Bernardo	0,00	86,48	172,38	133,55	161,63	227,19	188,75	197,44	262,42	252,22
Cundinamarca	San Cayetano	0,00	18,86	0,00	94,04	37,57	0,00	18,74	131,09	74,85	186,99
Cundinamarca	San Francisco	0,00	289,12	319,05	538,72	476,14	610,69	547,68	518,46	365,12	554,87
Cundinamarca	San Juan de Río Seco	0,00	30,81	41,15	195,71	371,29	299,37	268,62	320,45	72,39	72,40
Cundinamarca	Sasaima	9,71	222,44	346,15	325,51	419,25	588,07	339,94	583,15	654,94	670,27
Cundinamarca	Sesquilé	0,00	18,30	17,66	136,40	312,71	405,53	430,01	393,38	365,96	409,44
Cundinamarca	Sibaté	15,15	53,47	180,60	274,25	266,25	324,56	291,66	238,66	236,91	283,76
Cundinamarca	Silvania	13,93	203,61	235,30	243,94	491,23	544,87	575,68	578,88	564,05	517,71
Cundinamarca	Simijaca	8,78	172,44	279,66	133,23	270,16	362,41	221,61	350,17	390,00	398,65
Cundinamarca	Soacha	18,65	61,70	64,02	64,26	112,22	145,42	192,23	213,76	241,75	246,92
Cundinamarca	Sopó	0,00	4,38	94,08	192,17	53,09	251,47	343,60	442,97	384,77	504,77
Cundinamarca	Subachoque	14,63	85,95	336,61	171,69	127,83	369,05	316,39	329,32	304,03	212,96
Cundinamarca	Suesca	6,75	99,23	123,30	171,87	212,18	348,97	348,18	453,29	381,11	322,76
Cundinamarca	Supatá	0,00	0,00	40,16	20,04	220,04	100,00	179,60	438,42	477,90	437,12
Cundinamarca	Susa	9,83	86,36	28,11	64,02	107,23	156,99	85,19	74,86	56,90	71,47

Cundinamarca	Sutatausa	0,00	100,26	217,05	194,17	267,23	225,52	166,70	200,36	269,59	230,37
Cundinamarca	Tabio	9,08	154,61	215,06	222,08	306,28	322,44	236,83	333,45	369,92	415,13
Cundinamarca	Tausa	0,00	0,00	73,57	254,18	227,16	436,17	116,43	114,98	363,60	336,89
Cundinamarca	Tena	0,00	12,52	36,94	145,47	214,49	140,63	196,24	295,32	346,72	352,31
Cundinamarca	Tenjo	37,27	21,12	167,60	343,21	134,26	225,69	673,06	597,89	574,34	561,04
Cundinamarca	Tibacuy	41,47	124,17	248,91	186,49	290,04	310,82	248,45	269,65	186,41	558,89
Cundinamarca	Tibirita	0,00	0,00	268,01	233,96	605,65	438,30	608,73	611,21	508,47	373,39
Cundinamarca	Tocaima	0,00	142,50	175,35	224,54	384,85	409,97	313,70	415,60	348,07	589,16
Cundinamarca	Tocancipá	0,00	3,78	165,50	186,11	135,74	152,49	220,93	279,33	315,87	365,62
Cundinamarca	Topaipí	0,00	21,29	0,00	0,00	21,69	21,83	43,86	87,93	44,16	44,32
Cundinamarca	Ubalá	0,00	0,00	140,42	70,87	134,16	253,03	155,28	184,45	242,58	254,93
Cundinamarca	Ubaque	0,00	15,05	45,61	506,37	403,41	392,28	443,60	512,25	551,41	704,23
Cundinamarca	Une	0,00	11,97	200,59	162,98	57,36	203,69	279,14	187,29	293,61	246,89
Cundinamarca	Útica	0,00	100,99	181,52	100,64	160,90	260,99	340,82	200,20	139,78	498,41
Cundinamarca	Venecia	0,00	75,15	200,25	149,66	149,18	272,95	396,14	444,01	221,67	294,84
Cundinamarca	Vergara	13,05	104,37	78,31	104,38	143,53	195,77	299,91	247,69	143,29	156,17
Cundinamarca	Vianí	0,00	24,21	193,33	265,38	120,39	168,19	431,55	239,18	262,47	356,97
Cundinamarca	Villa de San Diego de Ubate	10,81	96,70	138,77	84,87	173,98	309,15	294,31	344,50	414,85	397,25
Cundinamarca	Villagómez	0,00	46,08	0,00	0,00	0,00	230,95	322,88	230,52	46,06	0,00
Cundinamarca	Villapinzón	0,00	5,72	5,62	22,08	65,11	31,98	89,09	118,49	167,16	169,53
Cundinamarca	Villeta	0,00	114,09	316,87	408,96	589,16	434,26	404,60	446,91	453,03	534,23
Cundinamarca	Viotá	14,88	409,87	253,58	343,21	418,47	493,64	441,45	486,82	314,58	622,10
Cundinamarca	Yacopí	0,00	6,04	12,03	71,98	35,85	29,78	65,31	88,77	100,29	64,67
Cundinamarca	Zipacón	0,00	57,95	324,43	188,75	149,70	314,76	531,14	380,64	412,93	461,73
Cundinamarca	Zipaquirá	4,72	40,77	136,36	130,28	78,84	134,23	177,56	213,61	241,12	274,97
Guainía	Barranco Minas	0,00	0,00	0,00	0,00	21,34	0,00	0,00	62,25	0,00	0,00
Guainía	Cacahual	0,00	0,00	0,00	0,00	0,00	0,00	0,00	0,00	0,00	0,00

Guainía	Inírida	92,91	81,05	0,00	111,08	99,50	155,59	282,57	341,12	277,55	270,23
Guainía	La Guadalupe	0,00	0,00	0,00	0,00	0,00	0,00	0,00	0,00	0,00	0,00
Guainía	Mapiripana	0,00	0,00	0,00	0,00	0,00	0,00	0,00	0,00	0,00	0,00
Guainía	Morichal	0,00	0,00	0,00	0,00	0,00	0,00	0,00	0,00	0,00	0,00
Guainía	Pana Pana	0,00	0,00	0,00	0,00	0,00	0,00	0,00	0,00	0,00	0,00
Guainía	Puerto Colombia	0,00	0,00	0,00	0,00	69,09	0,00	0,00	0,00	0,00	0,00
Guainía	San Felipe	0,00	0,00	0,00	0,00	0,00	54,98	105,60	50,74	0,00	0,00
Guaviare	Calamar	9,27	47,26	86,77	147,62	120,63	195,19	304,65	268,70	363,00	337,99
Guaviare	El Retorno	15,11	24,70	33,92	23,78	46,70	119,25	67,59	75,28	121,87	77,04
Guaviare	Miraflores	0,00	8,19	15,97	46,74	38,02	89,11	116,06	120,47	110,81	74,48
Guaviare	San José del Guaviare	51,66	20,99	22,33	60,72	134,25	185,72	166,57	162,22	139,42	138,70
Huila	Acevedo	173,79	24,78	6,92	37,26	182,25	113,47	44,42	189,39	267,39	315,26
Huila	Agrado	151,11	254,13	57,36	56,98	113,16	292,53	323,88	355,36	419,38	384,07
Huila	Aipe	33,34	23,14	9,00	43,74	72,30	198,60	225,38	207,55	255,38	196,66
Huila	Algeciras	202,57	4,20	79,35	41,56	12,42	82,45	193,03	298,85	216,40	284,97
Huila	Altamira	0,00	0,00	0,00	0,00	49,96	196,17	72,22	142,05	163,06	205,62
Huila	Baraya	237,27	42,94	42,73	53,16	21,18	189,53	157,35	292,43	301,67	207,34
Huila	Campoalegre	198,86	6,08	30,18	8,99	119,18	17,77	159,06	208,01	227,37	153,76
Huila	Colombia	17,52	34,68	0,00	50,90	92,37	99,65	254,81	178,96	209,42	111,70
Huila	Elías	0,00	0,00	28,15	55,40	136,13	267,31	184,07	129,37	407,12	351,05
Huila	Garzón	77,81	17,33	16,92	29,25	146,57	98,31	142,34	192,47	157,57	187,39
Huila	Gigante	0,00	0,00	23,22	42,39	25,64	40,99	164,35	161,66	90,03	144,68
Huila	Guadalupe	71,16	96,67	52,66	82,66	157,08	154,18	165,87	234,60	150,42	327,70
Huila	Hobo	120,48	29,93	44,59	44,37	249,96	102,46	58,25	43,53	187,64	273,11
Huila	Iquira	145,16	8,90	96,13	77,24	75,86	140,71	40,65	127,82	31,41	100,32
Huila	Isnos	188,74	0,00	55,87	55,11	7,77	68,96	177,68	100,79	25,79	116,43
Huila	La Argentina	8,30	122,06	0,00	7,83	84,54	0,00	251,74	334,40	185,44	154,16

Huila	La Plata	165,88	101,27	39,05	54,02	23,96	23,53	92,47	243,33	97,25	164,50
Huila	Nátaga	134,61	16,72	148,96	180,86	179,42	226,39	272,57	238,63	142,00	156,52
Huila	Neiva	196,52	87,69	117,16	147,99	286,46	333,84	369,69	354,95	337,31	391,54
Huila	Oporapa	26,52	8,64	76,01	33,05	56,57	158,10	92,78	264,63	251,42	274,77
Huila	Paicol	189,68	94,25	0,00	0,00	240,25	348,75	309,88	380,09	610,96	249,73
Huila	Palermo	273,25	132,38	37,59	13,41	157,89	142,09	253,68	177,54	195,83	159,38
Huila	Palestina	9,51	84,59	111,42	211,09	199,38	116,47	26,54	104,99	164,29	239,52
Huila	Pital	69,24	61,14	60,77	196,24	262,62	238,45	229,60	301,63	233,83	297,71
Huila	Pitalito	159,67	48,45	49,25	62,28	168,49	283,12	249,49	249,53	204,23	198,03
Huila	Rivera	251,26	236,60	62,71	163,31	590,00	401,61	261,10	301,22	239,41	363,27
Huila	Saladoblanco	104,86	47,11	74,48	18,41	218,68	81,02	338,71	290,98	331,47	319,49
Huila	San Agustín	135,21	6,53	12,91	15,98	12,65	78,26	340,88	380,53	465,13	578,28
Huila	Santa María	152,82	75,56	18,70	64,78	9,16	45,33	314,49	311,22	290,80	165,94
Huila	Suaza	276,46	160,07	6,23	18,16	264,83	286,14	222,69	314,11	210,88	230,84
Huila	Tarqui	104,56	36,51	0,00	5,96	5,90	29,21	63,67	143,38	159,05	185,78
Huila	Tello	190,42	0,00	0,00	7,23	7,19	64,42	135,26	106,25	84,54	161,14
Huila	Teruel	264,65	275,02	261,16	129,67	398,27	244,33	231,19	195,60	240,16	227,61
Huila	Tesalia	178,63	177,78	0,00	22,04	76,85	196,83	185,06	195,44	129,77	312,50
Huila	Timaná	154,43	14,93	24,84	14,88	29,73	351,19	266,73	379,65	251,00	353,81
Huila	Villavieja	13,59	27,21	13,62	54,51	54,58	81,87	191,07	191,36	109,41	287,08
Huila	Yaguará	348,69	61,49	48,46	23,91	23,58	325,70	263,88	351,00	335,23	253,75
La Guajira	Albania	9,04	79,17	141,55	100,43	126,70	71,95	35,20	114,96	120,27	70,11
La Guajira	Barrancas	17,81	221,25	198,14	62,07	190,86	254,23	205,69	159,53	268,64	194,95
La Guajira	Dibulla	0,00	48,11	30,73	11,05	28,28	37,36	107,79	138,41	100,05	49,73
La Guajira	Distracción	0,00	0,00	0,00	50,20	13,96	34,03	46,47	32,40	25,33	80,55
La Guajira	El Molino	78,52	77,11	138,57	160,91	243,25	502,57	153,18	151,04	103,12	67,90
La Guajira	Fonseca	0,00	17,28	13,51	26,44	12,95	139,62	177,48	238,57	285,68	216,07

La Guajira	Hatonuevo	27,82	0,00	0,00	9,78	4,69	18,02	8,66	120,83	16,05	3,87
La Guajira	La Jagua del Pilar	0,00	0,00	33,99	100,23	229,89	0,00	0,00	0,00	93,37	122,96
La Guajira	Maicao	36,56	114,86	57,76	19,73	17,21	68,72	149,87	131,53	120,34	92,69
La Guajira	Manaure	0,00	9,02	24,65	1,18	1,13	2,17	17,69	11,00	15,39	9,26
La Guajira	Riohacha	1,08	42,11	67,20	37,07	28,33	53,53	122,43	191,42	168,41	164,49
La Guajira	San Juan del Cesar	58,49	81,15	126,31	59,68	112,46	161,24	241,76	331,06	278,62	248,50
La Guajira	Uribia	0,00	0,00	4,30	4,14	2,65	12,78	23,40	14,86	6,31	6,65
La Guajira	Urumita	0,00	6,80	19,77	70,37	136,66	96,61	76,42	62,99	50,25	70,84
La Guajira	Villanueva	0,00	4,01	31,52	85,28	80,09	191,66	203,91	292,63	213,33	164,49
Magdalena	Algarrobo	41,87	66,59	66,18	8,22	0,00	0,00	137,11	576,92	166,98	340,00
Magdalena	Aracataca	5,52	0,00	0,00	16,06	7,95	7,86	5,18	7,69	38,00	200,38
Magdalena	Ariguaní	0,00	12,76	0,00	12,67	66,27	12,58	78,29	156,04	143,01	176,54
Magdalena	Cerro San Antonio	48,89	0,00	0,00	12,42	0,00	213,41	315,26	443,54	178,46	179,14
Magdalena	Chivolo	0,00	0,00	30,79	37,05	99,08	285,63	342,74	849,89	676,69	326,88
Magdalena	Ciénaga	1,95	19,49	1,94	3,88	1,94	1,93	37,58	198,92	208,95	253,30
Magdalena	Concordia	9,98	0,00	0,00	0,00	0,00	0,00	315,59	465,95	489,99	471,19
Magdalena	El Banco	41,92	23,68	16,38	9,09	32,68	30,81	85,02	74,00	142,27	159,89
Magdalena	El Piñon	0,00	0,00	0,00	0,00	83,27	720,45	262,36	256,61	232,81	340,46
Magdalena	El Retén	0,00	0,00	0,00	0,00	0,00	0,00	14,63	0,00	104,86	734,88
Magdalena	Fundación	12,26	5,25	8,75	1,75	8,75	15,74	19,22	8,73	62,78	169,00
Magdalena	Guamal	0,00	0,00	0,00	11,45	60,63	82,73	194,01	207,22	187,14	127,24
Magdalena	Nueva Granada	17,85	0,00	5,72	5,61	10,99	26,92	79,12	61,97	5,05	54,42
Magdalena	Pedraza	0,00	0,00	0,00	0,00	37,37	0,00	0,00	24,84	148,77	396,09
Magdalena	Pijiño del Carmen	6,95	0,00	6,81	6,73	53,28	138,28	188,77	334,25	177,68	181,72
Magdalena	Pivijay	2,81	0,00	5,70	5,73	8,64	142,02	306,01	246,23	247,61	204,43
Magdalena	Plato	19,70	1,94	7,63	0,00	101,58	181,70	153,69	86,13	174,60	139,40
Magdalena	Puebloviejo	0,00	0,00	7,38	0,00	0,00	0,00	105,84	311,36	269,19	312,22

Magdalena	Remolino	0,00	0,00	0,00	11,86	0,00	36,07	157,29	182,66	0,00	61,77
Magdalena	Sabanas de San Angel	97,92	12,90	12,74	18,90	0,00	30,77	60,80	174,21	456,57	134,60
Magdalena	Salamina	0,00	25,17	25,60	13,00	0,00	80,71	245,77	263,85	324,45	515,39
Magdalena	San Sebastián de Buenavista	11,56	5,77	0,00	17,28	0,00	0,00	28,68	40,10	245,95	211,31
Magdalena	San Zenón	0,00	0,00	0,00	0,00	188,49	66,37	485,54	440,33	417,26	339,54
Magdalena	Santa Ana	4,22	0,00	8,27	0,00	0,00	99,86	90,81	308,26	200,48	228,51
Magdalena	Santa Bárbara de Pinto	70,21	0,00	0,00	8,45	0,00	24,74	97,69	200,93	428,23	242,70
Magdalena	Santa Marta	69,16	54,08	24,03	25,67	40,45	67,11	138,36	211,80	145,50	183,10
Magdalena	Sitionuevo	0,00	0,00	0,00	0,00	0,00	0,00	42,09	41,53	44,16	68,56
Magdalena	Tenerife	0,00	48,27	72,63	48,55	16,22	24,39	73,30	261,16	204,20	155,36
Magdalena	Zapayán	0,00	0,00	0,00	0,00	0,00	137,35	251,46	113,83	90,90	226,22
Magdalena	Zona Bananera	0,00	5,18	0,00	1,71	0,00	11,80	83,75	188,01	211,49	260,91
Meta	Acacías	1,76	39,41	242,41	114,21	46,20	160,22	331,31	331,12	287,42	317,87
Meta	Barranca de Upía	0,00	29,25	57,31	28,18	275,94	297,06	238,66	571,28	382,07	349,39
Meta	Cabuyaro	0,00	159,49	78,99	208,71	154,80	281,55	253,10	351,67	572,42	568,18
Meta	Castilla la Nueva	0,00	141,42	62,34	108,88	305,02	489,36	629,49	546,45	551,39	495,00
Meta	Cubarral	0,00	0,00	0,00	90,11	141,89	69,97	344,95	459,42	470,90	298,51
Meta	Cumaral	0,00	70,45	17,46	28,85	62,94	34,05	411,13	592,34	577,14	550,96
Meta	El Calvario	0,00	131,69	0,00	0,00	133,04	132,80	0,00	88,69	0,00	0,00
Meta	El Castillo	0,00	178,17	299,13	165,91	30,39	91,91	247,37	327,36	298,65	539,60
Meta	El Dorado	0,00	119,69	59,58	0,00	0,00	29,44	762,69	760,46	554,10	611,00
Meta	Fuente de Oro	0,00	0,00	83,64	131,47	338,93	230,09	335,31	444,85	256,35	341,45
Meta	Granada	1,91	63,37	47,40	17,84	20,95	97,43	249,42	265,73	199,33	209,61
Meta	Guamal	11,13	0,00	66,06	21,87	97,89	378,75	258,56	686,55	469,78	361,59
Meta	La Macarena	3,87	11,24	21,81	7,05	30,79	66,41	90,28	78,30	79,12	41,41
Meta	Lejanías	0,00	10,48	10,49	0,00	0,00	94,96	169,22	392,07	372,22	448,05
Meta	Mapiripán	0,00	0,00	74,37	13,17	70,61	50,07	61,02	53,54	17,41	11,32

Meta	Mesetas	0,00	64,52	18,34	0,00	9,06	0,00	215,05	231,77	230,35	167,34
Meta	Puerto Concordia	0,00	11,54	11,23	27,32	10,64	72,48	141,22	78,58	28,71	32,65
Meta	Puerto Gaitán	5,68	22,56	16,80	22,26	77,40	170,26	218,28	341,63	301,79	283,76
Meta	Puerto Lleras	0,00	323,90	9,60	9,68	9,78	59,19	269,00	241,76	162,98	175,11
Meta	Puerto López	6,72	178,58	71,63	51,31	53,72	56,06	285,70	327,35	337,92	339,69
Meta	Puerto Rico	0,00	0,00	44,50	105,05	27,46	32,77	141,17	91,81	91,27	90,90
Meta	Restrepo	0,00	9,68	19,27	0,00	47,76	199,79	929,44	955,44	1179,36	1053,82
Meta	San Carlos de Guaroa	14,06	13,53	0,00	12,56	0,00	326,19	438,05	400,48	459,24	382,37
Meta	San Juan de Arama	0,00	76,91	187,60	199,47	0,00	223,51	359,11	326,80	362,11	272,73
Meta	San Juanito	51,89	0,00	50,03	49,50	0,00	288,74	190,02	234,30	92,38	0,00
Meta	San Martín	0,00	13,44	66,16	143,35	42,80	198,36	341,27	377,78	364,82	380,12
Meta	Uribe	0,00	14,75	7,19	0,00	0,00	6,66	32,50	69,74	30,95	18,14
Meta	Villavicencio	3,00	42,11	93,80	98,95	158,60	213,25	294,96	285,58	248,93	244,13
Meta	Vistahermosa	0,00	53,50	0,00	4,30	54,84	49,68	113,89	159,80	137,47	154,39
Nariño	Alban	181,48	29,81	14,70	38,67	38,13	84,63	69,58	73,23	140,08	84,74
Nariño	Aldana	223,88	45,30	30,54	201,02	218,85	348,10	288,65	389,42	394,41	283,14
Nariño	Ancuyá	0,00	36,05	49,15	25,12	0,00	65,73	471,44	303,49	550,61	955,83
Nariño	Arboleda	26,78	53,48	0,00	119,95	292,83	398,78	398,25	251,92	304,64	211,70
Nariño	Barbacoas	31,56	200,60	129,85	714,58	60,65	76,26	110,45	140,40	87,18	62,00
Nariño	Belén	44,38	87,59	0,00	0,00	0,00	13,83	40,97	121,31	279,33	144,51
Nariño	Buesaco	0,00	8,68	34,30	42,37	184,20	186,11	273,85	306,86	179,55	402,18
Nariño	Chachagüí	46,11	45,75	7,57	0,00	59,67	251,78	264,59	438,21	399,01	345,92
Nariño	Colón	153,45	40,71	121,68	10,09	10,04	99,95	179,07	79,33	187,62	245,87
Nariño	Consaca	276,65	39,87	241,40	172,55	92,22	20,67	250,47	548,23	255,70	623,92
Nariño	Contadero	104,06	44,46	14,74	29,36	29,24	160,00	275,52	490,97	373,89	516,28
Nariño	Córdoba	95,39	0,00	0,00	0,00	0,00	115,20	143,55	207,59	385,55	349,08
Nariño	Cuaspud	0,00	12,12	84,27	71,87	107,21	165,74	105,94	198,92	162,94	185,08

Nariño	Cumbal	117,94	30,43	5,96	5,85	8,61	92,85	71,78	113,74	138,17	203,40
Nariño	Cumbitara	24,78	16,06	54,58	22,73	88,30	21,46	34,76	67,54	72,18	44,64
Nariño	El Charco	10,84	0,00	0,00	3,25	50,14	51,38	34,99	75,94	29,85	39,26
Nariño	El Peñol	29,54	29,67	0,00	0,00	45,17	287,18	395,08	336,44	323,08	309,21
Nariño	El Rosario	0,00	9,05	9,14	0,00	0,00	28,37	133,91	154,99	9,80	39,75
Nariño	El Tablón de Gómez	7,28	0,00	0,00	0,00	0,00	7,62	153,81	225,14	250,84	229,61
Nariño	El Tambo	138,08	0,00	37,39	204,76	361,37	343,29	166,17	257,07	171,14	380,61
Nariño	Francisco Pizarro	0,00	0,00	0,00	0,00	15,02	0,00	98,99	75,42	19,95	0,00
Nariño	Funes	0,00	58,49	0,00	74,25	104,71	210,68	485,36	473,57	400,12	371,98
Nariño	Guachucal	36,16	0,00	48,85	18,45	24,76	343,13	502,77	380,16	319,45	469,70
Nariño	Guaitarilla	67,60	38,00	46,19	0,00	23,69	136,02	137,82	238,37	183,17	185,73
Nariño	Gualmatán	70,20	0,00	52,46	0,00	52,28	174,00	243,31	138,86	69,36	190,54
Nariño	Iles	87,72	12,40	0,00	0,00	11,99	23,71	23,48	11,61	91,94	113,82
Nariño	Imués	41,57	0,00	14,35	0,00	163,67	197,03	107,97	283,15	224,50	326,21
Nariño	Ipiales	61,95	11,92	62,30	88,37	117,94	136,83	164,61	242,73	263,20	329,90
Nariño	La Cruz	146,14	89,57	0,00	16,67	16,62	11,05	77,16	175,97	170,14	328,79
Nariño	La Florida	9,15	9,35	0,00	9,71	29,66	50,24	142,78	144,75	209,31	169,24
Nariño	La Llanada	0,00	0,00	63,76	64,54	0,00	49,63	318,26	458,09	172,03	523,93
Nariño	La Tola	43,97	42,22	40,56	97,55	65,53	44,92	146,67	57,98	0,00	0,00
Nariño	La Unión	217,87	21,93	7,36	55,55	171,52	503,02	412,00	624,41	502,34	598,78
Nariño	Leiva	24,56	0,00	0,00	0,00	7,67	7,55	29,74	65,86	72,21	7,11
Nariño	Linares	8,73	8,86	0,00	0,00	18,60	189,18	327,11	420,54	298,75	354,29
Nariño	Los Andes	11,88	17,50	0,00	45,03	5,53	135,85	181,45	136,28	417,22	207,36
Nariño	Magüi	5,71	11,07	16,11	36,44	10,09	4,89	23,71	18,39	44,57	4,32
Nariño	Mallama	134,23	11,37	69,47	58,87	23,99	36,63	99,45	316,58	193,42	249,74
Nariño	Mosquera	47,00	7,60	0,00	21,45	0,00	40,34	78,29	82,27	18,44	23,85
Nariño	Nariño	23,24	22,85	22,50	44,27	21,81	0,00	84,66	291,97	328,54	709,36

Nariño	Olaya Herrera	24,81	0,00	17,27	0,00	74,06	6,65	85,37	139,47	147,42	164,59
Nariño	Ospina	0,00	0,00	35,49	0,00	11,70	151,34	381,94	426,32	206,59	331,16
Nariño	Pasto	113,63	15,75	86,97	53,44	101,56	156,42	244,58	358,58	377,96	418,27
Nariño	Policarpa	180,89	0,00	6,68	13,10	25,67	56,64	18,51	60,55	41,58	99,07
Nariño	Potosí	162,14	15,57	86,39	95,12	79,83	48,29	154,07	196,13	197,74	249,29
Nariño	Providencia	0,00	8,21	0,00	8,01	15,82	54,70	46,31	122,04	60,35	104,38
Nariño	Puerres	101,47	11,35	34,25	23,00	46,32	58,34	164,32	343,24	107,35	240,41
Nariño	Pupiales	5,37	37,37	21,24	5,28	5,26	203,95	182,07	0,00	30,95	313,14
Nariño	Ricaurte	51,24	0,00	0,00	0,00	0,00	85,84	218,34	158,86	48,22	136,28
Nariño	Roberto Payán	0,00	0,00	0,00	5,11	0,00	0,00	4,69	4,55	0,00	0,00
Nariño	Samaniego	47,75	9,97	21,96	0,00	62,12	164,61	126,69	120,88	157,43	234,57
Nariño	San Andres de Tumaco	1,19	2,34	1,71	1,12	8,74	52,92	94,66	92,62	74,63	71,09
Nariño	San Bernardo	72,39	19,15	30,99	36,11	5,84	124,83	154,30	155,31	78,12	136,61
Nariño	San Lorenzo	42,85	10,63	36,92	5,23	36,34	56,67	133,02	187,80	171,29	304,97
Nariño	San Pablo	44,41	22,27	16,76	5,60	0,00	101,63	204,08	193,54	177,22	5,75
Nariño	San Pedro de Cartago	139,86	41,67	41,34	13,69	95,26	148,81	362,56	413,77	265,29	289,93
Nariño	Sandoná	130,20	31,49	47,14	109,79	66,54	308,74	308,30	296,23	311,47	556,23
Nariño	Santa Bárbara	6,58	6,61	6,63	0,00	6,67	6,69	26,89	33,74	0,00	6,81
Nariño	Santacruz	18,19	0,00	4,27	4,14	16,07	0,00	22,65	18,30	35,50	68,81
Nariño	Sapuyes	83,08	42,11	57,00	0,00	0,00	104,77	228,14	278,59	110,15	272,57
Nariño	Taminango	78,68	16,56	48,80	106,52	209,29	102,73	171,55	218,07	116,86	129,17
Nariño	Tangua	0,00	9,52	0,00	0,00	9,87	159,95	161,98	133,22	186,94	315,59
Nariño	Túquerres	77,56	24,28	46,20	9,75	21,97	136,98	191,21	203,94	140,40	182,73
Nariño	Yacuanquer	19,65	48,66	38,54	57,27	56,70	252,86	408,20	422,68	364,70	280,19
Norte de Santander	Abrego	34,11	2,82	25,10	8,28	103,93	100,19	104,62	98,30	126,33	125,12
Norte de Santander	Arboledas	65,54	32,88	11,00	11,02	121,57	265,60	221,80	111,12	345,06	211,77
Norte de Santander	Bochalema	30,07	44,87	0,00	0,00	44,08	481,82	304,52	403,81	516,28	413,11

Norte de Santander	Bucarasica	43,85	0,00	175,21	87,74	43,87	43,81	218,87	175,17	87,53	21,88
Norte de Santander	Cachirá	18,70	27,99	0,00	0,00	64,71	64,48	174,41	137,32	127,62	208,94
Norte de Santander	Cácota	164,27	84,75	86,96	0,00	276,50	190,39	440,10	654,91	363,64	747,46
Norte de Santander	Chinácota	132,69	118,29	65,03	19,31	490,54	485,56	205,99	321,38	360,90	320,96
Norte de Santander	Chitagá	29,36	29,30	19,50	9,73	19,42	67,85	58,06	28,97	134,97	538,93
Norte de Santander	Convención	100,71	173,35	65,48	0,00	252,06	347,39	333,17	383,09	294,79	278,28
Norte de Santander	Cúcuta	118,52	0,83	39,86	65,33	170,01	303,03	300,64	288,19	275,53	256,71
Norte de Santander	Cucutilla	24,19	0,00	12,34	0,00	176,19	114,18	191,91	322,54	377,31	183,61
Norte de Santander	Durania	312,73	0,00	24,75	25,09	127,06	565,70	832,25	420,50	345,01	455,15
Norte de Santander	El Carmen	126,09	6,40	110,55	19,80	93,89	95,40	221,41	224,92	299,89	246,56
Norte de Santander	El Tarra	27,80	9,26	27,73	18,47	73,71	45,95	82,57	219,78	410,70	209,59
Norte de Santander	El Zulia	110,70	4,76	122,26	41,82	151,61	86,21	174,85	381,05	267,04	393,82
Norte de Santander	Gramalote	0,00	0,00	66,64	50,61	0,00	17,30	35,06	35,40	125,74	163,28
Norte de Santander	Hacarí	0,00	0,00	77,60	48,25	48,00	19,10	161,41	122,72	150,14	251,82
Norte de Santander	Herrán	91,16	92,02	0,00	23,49	0,00	47,89	48,37	48,84	24,72	224,66
Norte de Santander	La Esperanza	26,87	8,87	8,79	43,63	8,65	222,72	195,08	117,67	66,60	90,74
Norte de Santander	La Playa	47,41	59,14	59,01	70,69	164,65	93,94	117,27	187,42	105,31	151,99
Norte de Santander	Labateca	273,79	51,35	273,93	17,12	85,56	153,90	68,34	392,56	119,31	238,26
Norte de Santander	Los Patios	141,91	175,77	239,79	136,46	224,04	417,90	348,34	433,98	375,01	325,27
Norte de Santander	Lourdes	29,18	0,00	0,00	0,00	117,89	59,12	236,83	118,73	178,31	118,98
Norte de Santander	Mutiscua	0,00	0,00	78,06	0,00	26,17	184,21	52,69	185,73	53,21	53,38
Norte de Santander	Ocaña	15,20	30,15	65,14	37,07	175,44	189,67	247,10	239,03	291,16	281,84
Norte de Santander	Pamplona	149,91	14,69	27,33	3,62	166,88	277,81	304,05	412,40	449,53	482,67
Norte de Santander	Pamplonita	41,49	0,00	123,71	0,00	61,50	102,21	265,04	121,98	364,96	121,33
Norte de Santander	Puerto Santander	11,10	0,00	0,00	10,58	20,80	194,53	271,82	89,21	126,84	86,36
Norte de Santander	Ragonvalia	14,66	14,63	14,61	14,59	14,58	116,47	116,33	130,74	29,02	391,47
Norte de Santander	Salazar	96,66	10,80	21,71	0,00	186,30	341,33	254,26	322,04	457,39	279,58

Norte de Santander	San Calixto	23,56	23,41	7,75	69,27	152,81	45,50	52,66	67,16	44,40	66,03
Norte de Santander	San Cayetano	43,01	0,00	207,04	0,00	159,46	351,84	325,55	319,25	239,68	198,66
Norte de Santander	Santiago	110,66	0,00	255,85	36,36	144,51	143,73	250,09	249,91	141,69	386,78
Norte de Santander	Sardinata	44,02	48,45	101,33	8,82	83,79	52,94	220,70	189,90	110,46	84,00
Norte de Santander	Silos	0,00	0,00	40,41	20,53	20,94	85,00	108,34	154,29	224,97	206,14
Norte de Santander	Teorama	0,00	0,00	15,80	15,48	70,74	59,36	53,30	52,17	55,75	86,45
Norte de Santander	Tibú	48,51	8,52	36,75	64,71	162,36	139,24	180,03	195,57	175,33	198,87
Norte de Santander	Toledo	98,41	0,00	5,79	17,36	190,99	109,96	590,28	671,26	416,59	358,71
Norte de Santander	Villa Caro	0,00	0,00	19,58	58,56	0,00	116,46	116,14	57,93	192,60	288,24
Norte de Santander	Villa del Rosario	159,75	10,67	15,63	52,13	304,23	424,50	347,10	356,40	328,95	318,08
Putumayo	Colón	114,92	0,00	18,92	0,00	167,97	92,78	276,40	200,91	380,50	468,13
Putumayo	Leguízamo	144,86	120,24	133,48	25,53	115,29	302,02	457,56	445,79	304,31	291,89
Putumayo	Mocoa	157,57	275,74	231,90	176,33	397,89	351,17	278,47	508,43	499,12	594,66
Putumayo	Orito	159,61	54,46	98,45	113,48	165,01	135,57	239,97	229,30	262,46	269,72
Putumayo	Puerto Asís	49,70	102,29	78,85	43,48	108,71	191,63	135,71	246,96	300,97	228,65
Putumayo	Puerto Caicedo	14,02	258,85	97,72	55,70	104,16	235,44	82,85	165,20	308,75	143,60
Putumayo	Puerto Guzmán	17,51	56,69	21,72	0,00	17,24	8,58	59,76	25,47	25,32	83,91
Putumayo	San Francisco	72,84	29,03	43,37	14,39	0,00	28,55	184,95	212,49	127,06	238,76
Putumayo	San Miguel	57,29	173,06	33,95	129,05	106,17	128,17	117,76	130,77	131,82	191,77
Putumayo	Santiago	10,57	20,87	41,20	0,00	40,19	39,71	78,49	184,27	230,15	265,53
Putumayo	Sibundoy	81,83	7,39	7,34	7,30	58,01	57,65	214,85	391,46	304,19	274,20
Putumayo	Valle del Guamuez	111,92	161,06	31,31	2,06	77,12	88,12	152,23	236,25	156,24	114,39
Putumayo	Villagarzón	67,23	191,92	91,07	134,07	172,19	167,05	209,48	318,00	293,37	282,82
Quindio	Armenia	2,46	4,90	76,92	33,58	177,29	284,89	386,57	403,87	345,15	369,22
Quindio	Buenavista	230,64	0,00	33,50	0,00	238,34	274,82	207,90	699,55	458,88	534,76
Quindio	Calarca	1,34	0,00	31,90	17,19	23,68	426,62	460,82	555,69	521,89	559,07
Quindio	Circasia	3,58	0,00	17,59	31,40	543,20	490,57	449,09	694,96	642,20	723,22

Quindio	Córdoba	0,00	0,00	204,38	242,18	37,36	205,80	206,57	583,04	603,20	679,76
Quindio	Filandia	0,00	22,95	152,51	151,93	454,13	218,79	262,96	419,26	685,95	712,96
Quindio	Génova	0,00	0,00	168,73	160,66	117,10	95,47	559,27	408,72	618,61	527,53
Quindio	La Tebaida	0,00	0,00	8,17	29,27	200,29	111,85	556,56	538,99	464,86	412,43
Quindio	Montenegro	0,00	4,96	9,89	22,17	319,15	506,47	485,25	568,62	418,98	470,58
Quindio	Pijao	0,00	0,00	15,49	31,25	471,77	698,41	336,38	725,46	570,13	754,47
Quindio	Quimbaya	0,00	0,00	5,81	8,69	326,55	625,59	439,95	610,98	463,55	479,51
Quindio	Salento	0,00	97,30	69,66	41,86	405,08	168,09	140,27	365,37	463,81	562,67
Risaralda	Apía	95,49	44,57	55,24	175,35	228,26	302,05	326,34	281,33	247,60	256,16
Risaralda	Balboa	47,28	0,00	31,58	0,00	0,00	78,89	457,99	442,48	173,61	410,68
Risaralda	Belén de Umbría	198,39	209,24	165,94	227,26	288,62	335,43	389,57	375,11	270,58	331,84
Risaralda	Dosquebradas	48,57	72,89	87,09	124,79	202,54	219,66	270,80	275,74	223,25	305,23
Risaralda	Guática	102,11	159,98	224,65	205,91	225,94	278,39	305,27	384,26	339,65	301,34
Risaralda	La Celia	57,29	160,72	126,66	80,67	207,88	185,06	23,17	197,33	279,36	338,00
Risaralda	La Virginia	66,84	3,17	6,33	18,95	274,20	600,48	605,28	534,93	558,73	470,23
Risaralda	Marsella	68,78	0,00	13,54	161,07	306,26	299,30	440,89	411,18	441,98	463,75
Risaralda	Mistrató	104,39	12,97	167,67	25,63	25,46	18,97	12,55	168,19	24,71	128,69
Risaralda	Pereira	99,09	171,76	138,84	148,77	197,10	249,02	281,03	323,43	285,34	287,92
Risaralda	Pueblo Rico	0,00	0,00	56,37	119,51	118,27	85,77	161,92	137,14	165,50	141,18
Risaralda	Quinchía	131,78	146,57	134,44	95,50	32,78	119,04	231,79	359,03	355,48	369,65
Risaralda	Santa Rosa de Cabal	83,75	31,12	124,07	151,76	147,10	68,43	199,14	341,55	328,12	403,13
Risaralda	Santuario	135,76	77,44	25,76	0,00	115,47	275,46	440,87	465,44	502,51	399,97
Santander	Aguada	97,13	0,00	0,00	0,00	0,00	103,79	52,49	320,00	377,36	164,02
Santander	Albania	21,79	0,00	0,00	20,95	20,69	0,00	40,31	59,75	78,49	174,35
Santander	Aratoca	0,00	0,00	23,91	11,96	95,82	311,75	408,11	228,31	276,71	421,64
Santander	Barbosa	60,20	152,74	184,41	233,82	224,43	462,75	387,46	461,46	467,96	474,49
Santander	Barichara	118,89	39,88	13,37	429,70	135,30	149,62	465,37	605,98	429,66	586,10

Santander	Barrancabermeja	0,52	1,57	16,20	106,53	324,59	329,13	336,87	342,57	320,18	330,20
Santander	Betulia	18,88	0,00	19,03	76,48	153,52	134,93	329,90	272,69	78,28	98,52
Santander	Bolívar	0,00	22,27	45,10	30,45	69,38	163,88	142,20	112,02	113,35	131,10
Santander	Bucaramanga	60,01	44,30	50,31	52,10	139,78	230,01	229,87	234,71	211,97	215,99
Santander	Cabrera	151,06	0,00	48,88	0,00	141,51	46,43	91,37	313,90	88,22	259,40
Santander	California	0,00	53,94	106,84	52,99	52,69	104,17	257,20	255,62	655,24	398,80
Santander	Capitanejo	0,00	66,96	33,77	17,06	51,67	573,81	491,06	460,26	464,87	523,18
Santander	Carcasí	0,00	0,00	0,00	38,80	331,06	117,53	117,97	275,97	218,30	199,16
Santander	Cepitá	0,00	0,00	0,00	51,55	259,07	208,88	52,69	159,49	268,10	108,34
Santander	Cerrito	0,00	16,33	16,48	149,73	235,37	186,76	257,42	450,53	420,46	477,03
Santander	Charalá	26,73	8,98	0,00	63,85	128,72	268,67	177,40	150,59	170,78	191,31
Santander	Charta	98,78	0,00	0,00	137,69	35,22	285,51	180,96	294,33	262,17	303,38
Santander	Chima	152,25	0,00	92,82	0,00	94,55	95,21	256,16	257,48	129,58	195,95
Santander	Chipatá	0,00	77,88	38,98	117,07	117,23	117,39	137,15	215,86	176,89	334,65
Santander	Cimitarra	33,11	94,02	54,93	155,42	146,41	190,94	178,98	190,44	167,66	187,36
Santander	Concepción	17,27	0,00	0,00	285,61	361,21	292,18	942,18	355,47	321,24	114,72
Santander	Confines	36,31	0,00	0,00	0,00	0,00	257,54	257,83	517,18	554,53	259,55
Santander	Contratación	25,67	25,95	26,34	26,64	189,19	576,45	305,90	338,31	257,81	202,84
Santander	Coromoro	0,00	0,00	40,24	80,30	80,10	13,33	79,78	66,28	105,85	171,41
Santander	Curití	0,00	8,62	8,59	17,11	255,75	314,33	389,20	539,45	428,61	376,82
Santander	El Carmen de Chucurí	5,40	74,81	37,02	62,80	10,36	174,35	25,39	125,60	154,24	64,05
Santander	El Guacamayo	0,00	0,00	45,81	92,89	235,74	143,61	48,50	245,82	199,50	559,23
Santander	El Peñón	0,00	36,83	37,12	18,72	113,31	57,11	172,51	173,78	136,19	215,10
Santander	El Playón	7,78	47,21	0,00	24,10	32,51	361,34	224,33	302,24	365,15	317,71
Santander	Encino	0,00	0,00	0,00	0,00	77,73	0,00	0,00	119,19	0,00	80,65
Santander	Enciso	156,29	26,48	81,06	1073,49	645,16	628,21	58,09	709,64	571,77	705,95
Santander	Florián	47,15	204,53	173,28	236,56	236,85	63,24	110,81	15,85	174,58	63,56

Santander	Floridablanca	19,03	42,11	53,85	102,64	148,80	180,58	198,18	213,83	194,80	228,15
Santander	Galán	35,46	0,00	0,00	38,14	0,00	40,11	206,19	548,99	216,36	133,69
Santander	Gambita	58,64	19,59	0,00	39,39	39,46	177,94	79,16	99,13	118,95	198,49
Santander	Girón	15,96	29,66	46,51	124,79	113,28	166,75	149,91	158,95	142,48	172,68
Santander	Guaca	0,00	0,00	0,00	15,04	197,00	168,04	154,06	248,18	78,19	141,84
Santander	Guadalupe	55,26	0,00	19,05	116,14	216,19	420,25	244,00	330,65	357,44	384,45
Santander	Guapotá	267,26	0,00	90,17	0,00	365,63	415,90	416,67	466,85	233,75	94,34
Santander	Guavatá	23,64	0,00	0,00	199,40	304,26	181,07	210,14	187,32	163,09	635,36
Santander	Güepsa	383,23	169,61	170,57	371,38	599,70	226,99	203,98	515,73	779,42	867,51
Santander	Hato	41,75	0,00	0,00	126,16	126,42	253,70	634,79	297,87	469,08	341,88
Santander	Jesús María	0,00	0,00	0,00	30,38	153,14	30,92	218,48	535,94	350,65	450,60
Santander	Jordán	0,00	0,00	0,00	0,00	89,05	0,00	0,00	0,00	0,00	90,99
Santander	La Belleza	47,06	93,95	70,34	105,35	128,58	198,48	174,95	151,50	81,52	81,47
Santander	La Paz	90,81	0,00	55,37	55,80	150,15	56,74	152,56	96,12	174,69	176,33
Santander	Landázuri	0,00	19,69	6,55	19,64	78,43	182,74	228,24	169,28	266,68	227,35
Santander	Lebríja	169,90	99,67	94,55	225,50	288,49	232,44	330,39	365,67	355,29	398,50
Santander	Los Santos	8,91	96,88	26,15	120,77	187,91	109,91	175,79	149,19	172,34	284,58
Santander	Macaravita	0,00	0,00	0,00	0,00	159,17	242,03	328,27	124,84	84,10	214,04
Santander	Málaga	0,00	10,75	53,82	323,42	280,75	297,52	325,12	325,63	582,09	621,19
Santander	Matanza	0,00	70,51	88,97	287,46	163,37	458,55	424,90	524,15	377,57	305,46
Santander	Mogotes	109,72	82,39	36,63	210,76	238,42	220,18	311,90	358,16	294,12	349,10
Santander	Molagavita	17,76	17,96	0,00	0,00	129,61	149,76	132,28	229,62	231,08	175,44
Santander	Ocamonte	60,68	20,32	0,00	0,00	185,22	186,10	395,01	417,54	293,19	505,48
Santander	Oiba	27,04	53,68	0,00	0,00	183,98	530,99	388,84	506,09	494,12	423,19
Santander	Onzaga	126,17	54,81	0,00	0,00	37,84	171,95	154,56	195,31	178,08	159,90
Santander	Palmar	0,00	0,00	0,00	32,27	0,00	0,00	92,91	30,45	30,03	148,19
Santander	Palmas del Socorro	41,61	251,99	42,32	213,68	215,61	653,59	218,82	530,04	490,85	493,94

Santander	Páramo	0,00	0,00	0,00	0,00	126,90	528,30	198,61	319,88	607,98	481,00
Santander	Piedecuesta	18,64	50,60	36,27	158,98	208,93	183,22	150,19	209,81	192,30	184,01
Santander	Pinchote	219,44	21,57	148,40	62,62	164,17	181,60	556,33	117,32	57,68	340,59
Santander	Puente Nacional	7,11	7,22	0,00	0,00	113,16	45,96	194,40	299,97	320,62	399,35
Santander	Puerto Parra	29,84	0,00	14,48	28,54	70,34	111,03	136,67	26,94	39,75	78,38
Santander	Puerto Wilches	6,35	85,72	120,63	161,92	133,37	76,18	82,52	199,91	95,20	139,64
Santander	Rionegro	20,76	69,75	24,61	24,81	207,22	230,42	214,15	369,56	302,43	271,42
Santander	Sabana de Torres	163,13	128,04	154,43	222,59	98,95	550,40	506,76	462,72	407,46	594,82
Santander	San Andrés	42,12	95,98	0,00	219,01	233,23	135,07	307,73	381,06	222,48	154,17
Santander	San Benito	0,00	0,00	76,18	126,65	75,91	25,28	25,24	125,79	175,61	25,03
Santander	San Gil	161,63	9,06	27,05	67,32	319,55	396,11	348,01	291,49	336,67	372,77
Santander	San Joaquín	175,99	71,71	72,78	333,58	226,33	76,31	429,35	632,91	361,74	449,90
Santander	San José de Miranda	21,16	0,00	0,00	65,39	286,66	200,58	518,25	318,54	230,10	395,07
Santander	San Miguel	0,00	0,00	0,00	0,00	120,19	325,07	410,17	249,69	378,31	383,14
Santander	San Vicente de Chucurí	98,38	17,82	5,91	8,83	46,90	236,50	223,98	228,91	219,40	184,12
Santander	Santa Bárbara	0,00	0,00	89,73	225,53	318,33	457,46	462,32	279,59	467,95	329,88
Santander	Santa Helena del Opón	0,00	45,22	90,81	22,80	0,00	22,99	23,09	23,15	23,23	0,00
Santander	Simacota	34,56	93,51	35,57	312,20	194,72	160,36	287,64	253,52	320,97	299,17
Santander	Socorro	74,97	77,93	77,51	164,21	70,00	335,08	316,88	479,68	346,67	413,45
Santander	Suaita	9,24	27,91	0,00	0,00	151,63	238,62	211,38	270,85	282,18	254,60
Santander	Sucre	22,09	11,15	78,86	0,00	57,37	69,46	81,79	94,45	142,91	132,15
Santander	Suratá	0,00	0,00	0,00	57,49	87,31	117,72	386,67	120,30	121,40	91,91
Santander	Tona	29,61	14,71	43,89	130,72	57,75	229,82	42,85	127,73	56,46	70,14
Santander	Valle de San José	38,80	58,93	59,71	20,15	428,13	144,60	523,23	592,59	449,68	477,53
Santander	Vélez	61,26	107,57	30,83	30,94	362,24	249,29	99,04	224,92	173,16	410,68
Santander	Vetas	42,28	0,00	0,00	0,00	208,51	0,00	41,39	0,00	41,07	0,00
Santander	Villanueva	148,26	0,00	76,69	15,60	317,81	500,57	559,03	368,32	682,83	660,52

Santander	Zapatoca	0,00	10,83	0,00	21,87	131,74	220,73	255,02	200,69	246,39	213,70
Sucre	Buenavista	0,00	0,00	0,00	64,79	96,61	224,00	233,20	210,48	303,60	280,72
Sucre	Caimito	71,12	17,63	0,00	164,69	292,02	34,06	67,45	66,88	165,60	114,90
Sucre	Chalán	0,00	117,92	0,00	140,35	326,57	92,92	370,20	161,25	206,71	389,28
Sucre	Coloso	0,00	245,78	33,03	199,20	300,55	168,18	287,40	476,35	513,87	430,81
Sucre	Corozal	6,80	5,06	6,69	76,39	84,06	88,34	191,69	293,59	200,29	213,27
Sucre	Coveñas	85,19	451,54	41,09	266,41	460,24	514,34	520,67	466,17	524,76	362,87
Sucre	El Roble	0,00	41,09	10,15	50,18	178,59	147,06	242,44	201,30	132,70	93,72
Sucre	Galeras	0,00	0,00	0,00	75,10	337,84	145,51	240,34	171,15	302,16	219,35
Sucre	Guaranda	0,00	105,99	0,00	91,49	144,69	11,91	70,63	81,39	91,84	45,34
Sucre	La Unión	19,10	37,89	9,40	83,99	203,57	36,71	72,84	45,15	170,10	115,43
Sucre	Los Palmitos	0,00	0,00	0,00	15,56	0,00	10,37	83,03	140,07	72,70	176,67
Sucre	Majagual	9,40	21,82	15,51	58,66	128,99	24,44	151,96	120,93	84,19	77,76
Sucre	Morroa	60,88	112,82	22,30	227,74	355,74	430,85	397,53	378,60	325,73	363,44
Sucre	Ovejas	74,30	27,93	107,39	56,17	159,60	94,08	174,56	151,36	260,77	213,98
Sucre	Palmito	0,00	58,29	40,88	144,49	102,41	23,21	83,49	156,40	358,13	222,17
Sucre	Sampués	0,00	0,00	0,00	61,83	259,71	18,67	369,25	434,01	403,43	507,00
Sucre	San Benito Abad	4,27	8,45	4,18	33,15	90,21	85,21	104,42	39,73	39,31	42,76
Sucre	San Juan de Betulia	0,00	32,17	0,00	72,19	88,11	16,00	343,59	215,50	247,13	270,77
Sucre	San Luis de Sincé	6,43	19,09	12,60	12,47	49,40	73,37	87,78	128,89	100,93	173,45
Sucre	San Marcos	129,17	209,63	43,32	80,04	237,29	92,67	211,86	193,32	187,49	257,90
Sucre	San Onofre	0,00	86,49	23,02	58,13	123,54	157,27	137,73	176,76	175,25	175,73
Sucre	San Pedro	0,00	12,25	0,00	264,62	302,28	278,34	421,78	304,82	586,11	281,16
Sucre	Santiago de Tolú	41,30	33,83	9,98	291,04	382,53	426,66	186,41	287,19	321,36	248,00
Sucre	Sincelejo	95,88	8,84	15,05	127,22	242,68	238,46	304,59	319,12	260,17	240,12
Sucre	Sucre	4,46	22,33	22,34	13,41	35,77	26,83	76,01	75,98	35,74	93,74
Sucre	Tolú Viejo	100,33	21,13	79,26	253,73	259,09	550,03	354,44	391,53	291,05	211,70

Tolima	Alpujarra	116,64	0,00	176,13	117,83	157,67	178,04	119,19	259,27	180,29	261,36
Tolima	Alvarado	156,28	55,89	67,20	44,89	292,60	349,34	428,94	361,70	384,88	465,06
Tolima	Ambalema	53,26	215,63	27,26	358,67	850,89	635,23	485,71	651,42	248,65	444,12
Tolima	Anzoátegui	53,44	123,28	5,80	11,48	107,84	151,56	177,63	137,20	135,62	85,85
Tolima	Armero	155,81	7,86	15,85	231,83	749,94	626,58	829,30	670,86	426,42	726,41
Tolima	Ataco	4,54	63,41	0,00	4,51	62,94	125,53	138,57	102,49	102,16	79,68
Tolima	Cajamarca	30,36	30,38	10,13	152,08	319,60	309,66	396,24	264,35	330,69	285,12
Tolima	Carmen de Apicalá	211,89	468,71	256,50	395,12	612,93	633,64	573,72	947,49	693,73	837,58
Tolima	Casabianca	58,13	58,33	14,62	14,68	132,68	88,65	207,78	119,14	314,18	330,28
Tolima	Chaparral	96,12	153,64	61,84	157,68	251,17	214,77	261,25	324,64	256,38	190,48
Tolima	Coello	10,92	65,05	32,28	181,43	296,61	273,11	260,63	248,24	297,74	224,26
Tolima	Coyaima	17,84	17,83	3,56	67,57	206,04	113,57	113,39	187,59	127,19	144,70
Tolima	Cunday	76,53	67,60	68,17	49,16	69,46	150,20	161,65	50,94	0,00	207,60
Tolima	Dolores	89,66	0,00	0,00	151,16	105,89	178,74	289,12	414,38	333,00	274,49
Tolima	Espinal	49,78	51,06	115,19	168,84	459,44	534,19	487,29	564,94	543,11	506,90
Tolima	Falan	0,00	75,58	10,80	64,86	54,08	54,12	249,13	336,04	227,82	260,56
Tolima	Flandes	3,54	28,21	42,15	178,37	386,77	350,68	422,10	465,39	453,51	397,27
Tolima	Fresno	93,18	41,89	35,57	90,85	283,21	277,62	347,54	417,83	432,57	384,55
Tolima	Guamo	154,32	70,36	20,67	133,82	362,51	461,83	425,83	499,57	432,46	395,48
Tolima	Herveo	0,00	0,00	22,89	69,52	93,83	189,96	192,49	255,79	259,26	187,31
Tolima	Honda	100,94	283,04	38,10	196,08	283,45	266,47	253,33	299,62	290,55	293,31
Tolima	Ibagué	59,63	27,16	107,87	117,75	195,29	197,59	216,99	259,03	224,02	247,49
Tolima	Icononzo	112,58	8,70	0,00	105,59	212,45	302,92	322,93	234,93	173,01	275,38
Tolima	Lérida	62,78	258,97	74,78	129,46	441,51	418,59	578,87	523,00	386,72	557,63
Tolima	Líbano	26,25	122,23	21,67	58,02	247,63	234,08	252,31	236,24	308,98	295,53
Tolima	Mariquita	39,37	202,64	108,70	205,10	436,97	370,10	487,09	627,93	507,37	519,07
Tolima	Melgar	53,71	342,55	172,61	272,38	493,76	609,08	646,18	777,64	485,48	773,27

Tolima	Murillo	196,54	98,50	19,71	0,00	79,13	79,13	99,25	19,87	19,88	79,71
Tolima	Natagaima	112,70	152,13	0,00	113,59	438,10	267,98	224,69	278,31	172,77	182,09
Tolima	Ortega	53,78	15,01	48,22	208,79	103,26	204,21	229,36	174,79	178,33	160,34
Tolima	Palocabildo	52,53	21,08	10,59	212,81	213,65	118,13	215,59	443,72	195,72	272,93
Tolima	Piedras	54,92	18,24	0,00	54,29	36,05	233,52	250,72	339,16	302,54	212,77
Tolima	Planadas	203,00	303,95	74,17	266,00	198,39	104,05	238,00	287,75	183,73	156,80
Tolima	Prado	35,03	212,56	23,93	48,39	134,61	322,22	489,46	558,45	513,41	597,33
Tolima	Purificación	14,20	95,35	52,69	276,21	427,87	477,76	406,73	449,34	317,58	166,60
Tolima	Rioblanco	133,67	130,32	55,56	15,94	80,05	72,38	88,87	77,05	97,75	155,36
Tolima	Roncesvalles	0,00	31,82	0,00	0,00	15,87	31,73	94,98	189,90	173,75	126,10
Tolima	Rovira	9,33	9,37	23,54	156,09	237,64	363,05	455,77	409,66	295,53	413,79
Tolima	Saldaña	174,31	174,97	40,49	284,34	489,43	600,68	514,19	371,72	346,09	493,57
Tolima	San Antonio	6,60	79,73	80,29	175,10	304,92	225,07	329,33	220,95	284,72	286,51
Tolima	San Luis	41,57	15,60	78,02	156,11	98,92	218,80	260,61	318,12	323,52	287,16
Tolima	Santa Isabel	30,64	15,36	0,00	30,88	61,94	62,14	15,57	78,09	0,00	31,46
Tolima	Suárez	44,07	0,00	0,00	44,11	220,07	132,19	198,02	132,01	329,31	175,94
Tolima	Valle de San Juan	80,61	0,00	16,03	111,91	159,41	222,54	63,32	378,85	409,13	361,18
Tolima	Venadillo	5,29	0,00	15,71	250,10	326,73	118,81	149,20	143,47	194,02	183,19
Tolima	Villahermosa	108,03	81,41	9,08	100,29	100,71	147,17	249,58	130,22	215,03	281,64
Tolima	Villarrica	33,02	0,00	16,95	0,00	34,79	176,43	357,21	217,39	146,82	222,68
Valle del Cauca	Alcalá	21,94	430,43	316,57	320,63	365,24	412,73	438,77	444,34	430,63	403,69
Valle del Cauca	Andalucía	22,17	472,22	166,92	239,61	262,23	368,94	436,61	291,51	258,21	500,37
Valle del Cauca	Ansermanuevo	9,78	216,51	197,90	258,69	280,15	417,59	389,46	340,69	342,48	293,04
Valle del Cauca	Argelia	120,59	242,06	197,42	365,69	504,82	337,53	261,82	402,35	372,56	342,73
Valle del Cauca	Bolívar	140,40	182,96	20,59	41,73	148,00	299,98	245,90	410,20	474,78	533,71
Valle del Cauca	Buenaventura	3,51	26,07	20,80	27,29	17,57	27,05	22,37	20,66	13,51	23,79
Valle del Cauca	Bugalagrande	0,00	9,31	4,66	247,64	463,48	553,68	432,66	400,57	543,20	444,99

Valle del Cauca	Caicedonia	3,26	13,10	473,05	458,13	568,82	547,59	599,36	551,32	492,79	602,04
Valle del Cauca	Cali	3,78	152,00	90,64	118,95	151,92	171,83	162,91	204,03	175,41	187,81
Valle del Cauca	Calima	77,19	423,92	218,05	262,50	357,74	484,63	477,34	641,96	558,27	500,25
Valle del Cauca	Candelaria	55,20	174,02	65,62	80,48	51,98	204,84	97,10	285,77	208,08	37,39
Valle del Cauca	Cartago	2,38	205,59	256,74	256,72	281,47	318,28	240,79	286,59	235,16	239,17
Valle del Cauca	Dagua	126,74	151,63	274,41	237,32	567,75	540,98	625,02	675,27	552,11	561,27
Valle del Cauca	El Águila	37,19	166,84	184,74	220,87	265,98	219,28	163,83	172,32	108,31	215,91
Valle del Cauca	El Cairo	63,34	0,00	0,00	155,31	401,40	398,90	274,20	282,43	230,37	218,97
Valle del Cauca	El Cerrito	1,81	9,01	12,56	139,21	102,98	166,05	268,93	248,35	142,70	181,83
Valle del Cauca	El Dovio	42,90	314,67	175,84	155,57	224,82	204,73	379,27	418,75	423,13	344,99
Valle del Cauca	Florida	1,77	100,61	360,57	250,60	282,86	229,63	251,30	404,07	294,17	377,08
Valle del Cauca	Ginebra	40,85	35,44	120,45	24,87	113,36	102,54	416,16	608,91	555,90	640,33
Valle del Cauca	Guacarí	3,10	15,36	158,33	202,33	224,59	302,97	347,52	347,61	269,37	396,56
Valle del Cauca	Guadalajara de Buga	9,44	191,62	277,01	204,13	248,39	225,44	188,57	202,72	249,06	352,09
Valle del Cauca	Jamundí	2,96	306,68	56,85	285,80	328,98	380,97	314,71	381,67	317,82	387,48
Valle del Cauca	La Cumbre	277,08	490,07	302,14	433,71	238,20	518,50	691,89	924,63	807,71	821,52
Valle del Cauca	La Unión	115,48	253,91	237,91	138,60	119,22	195,33	235,93	428,85	262,52	224,19
Valle del Cauca	La Victoria	21,51	187,54	29,04	270,19	242,59	273,71	402,20	434,75	150,91	448,09
Valle del Cauca	Obando	55,26	406,11	411,44	266,36	312,88	406,28	363,95	442,63	560,82	358,52
Valle del Cauca	Palmira	24,62	229,30	125,47	148,01	203,97	262,16	285,00	277,15	248,08	225,62
Valle del Cauca	Pradera	4,00	400,98	314,20	364,39	453,32	445,97	368,00	440,61	346,35	342,04
Valle del Cauca	Restrepo	100,68	100,45	262,81	343,28	442,01	465,75	427,38	432,50	505,17	675,84
Valle del Cauca	Riofrío	83,62	315,84	240,70	269,69	325,09	362,72	335,68	488,07	312,39	400,11
Valle del Cauca	Roldanillo	125,36	278,57	306,69	296,58	363,86	638,81	555,00	503,59	460,63	585,87
Valle del Cauca	San Pedro	0,00	103,29	275,61	230,47	349,61	448,35	272,05	335,53	352,95	369,97
Valle del Cauca	Sevilla	27,48	25,51	0,00	98,92	131,93	398,13	474,88	468,90	443,04	363,23
Valle del Cauca	Toro	118,83	324,39	304,93	273,00	173,17	80,16	104,44	85,72	91,51	303,80

Valle del Cauca	Trujillo	113,15	86,45	173,39	168,46	119,93	185,90	274,38	346,95	436,39	332,65
Valle del Cauca	Tuluá	23,95	93,59	38,61	38,14	113,54	166,06	189,25	218,09	218,35	170,02
Valle del Cauca	Ulloa	17,54	35,27	531,35	374,06	661,90	504,23	670,29	564,66	384,97	701,62
Valle del Cauca	Versalles	0,00	37,78	293,63	413,97	131,25	266,17	418,30	519,27	332,64	393,31
Valle del Cauca	Vijes	40,00	148,27	39,07	202,66	76,24	160,06	213,99	468,49	490,11	421,83
Valle del Cauca	Yotoco	0,00	6,35	315,88	238,98	256,62	299,01	303,99	345,79	276,63	434,57
Valle del Cauca	Yumbo	4,13	405,26	301,19	318,11	374,44	384,86	369,57	417,73	282,53	301,00
Valle del Cauca	Zarzal	4,79	234,67	180,69	204,46	195,53	321,13	342,79	386,27	353,72	418,10
Vaupés	Carurú	0,00	0,00	0,00	0,00	0,00	30,21	30,15	120,41	30,06	0,00
Vaupés	Mitú	48,18	0,00	0,00	66,59	115,33	127,21	251,92	332,64	294,60	260,51
Vaupés	Pacoa	298,06	269,82	465,59	533,18	289,07	395,03	183,65	986,37	245,23	0,00
Vaupés	Papunahua	0,00	0,00	0,00	0,00	116,14	700,12	117,23	0,00	0,00	0,00
Vaupés	Taraira	0,00	0,00	0,00	98,52	3972,19	1100,00	806,45	1524,39	5840,16	1033,06
Vaupés	Yavarate	0,00	0,00	396,51	79,49	1752,99	2158,27	80,13	80,39	0,00	80,97
Vichada	Cumaribo	0,00	3,22	0,00	15,31	11,95	32,06	25,61	44,45	16,27	39,75
Vichada	La Primavera	8,72	209,98	258,92	155,95	37,59	43,51	132,92	101,28	91,25	144,78
Vichada	Puerto Carreño	0,00	121,17	231,16	337,42	115,12	266,45	412,90	883,59	628,45	643,75
Vichada	Santa Rosalía	116,96	114,22	251,33	218,64	26,78	131,34	128,97	278,76	224,33	122,67
