# Supplementary material for: Epidemiological scenarios for human rabies exposure notified in Colombia during ten years: A challenge to implement surveillance actions with a differential approach on vulnerable populations
Source: PLoS One. 2019 Dec 27;14(12):e0213120. doi: 10.1371/journal.pone.0213120 (PMC6934280; doi:10.1371/journal.pone.0213120)
Supplement: S2 Table — (PDF) [file pone.0213120.s002.pdf]

TABLE. Mean incidence of HRE by animal aggressor type in all cities of Colombia (2006-2017)

DEPARTMENT	CITY	DOG	CAT	BAT	FARM ANIMALS
Amazonas	Miriti - Paraná	18,90	0,00	6,53	0,00
Amazonas	Leticia	258,11	21,33	1,99	1,73
Amazonas	El Encanto	12,83	0,00	0,00	0,00
Amazonas	La Chorrera	10,58	0,00	0,00	0,00
Amazonas	La Pedrera	19,67	0,00	0,00	0,00
Amazonas	La Victoria	113,50	0,00	0,00	0,00
Amazonas	Puerto Alegría	23,33	0,00	0,00	0,00
Amazonas	Puerto Arica	7,20	0,00	0,00	0,00
Amazonas	Puerto Nariño	77,26	0,00	0,00	1,26
Amazonas	Puerto Santander	15,19	3,78	0,00	0,00
Amazonas	Tarapacá	43,94	2,53	0,00	0,00
Antioquia	Guatapé	444,65	27,77	5,64	0,00
Antioquia	Carolina	73,68	5,43	2,76	0,00
Antioquia	Nariño	309,91	9,44	2,38	1,21
Antioquia	Cisneros	170,79	31,53	2,20	1,10
Antioquia	Liborina	39,89	2,10	2,10	0,00
Antioquia	Hispania	168,70	20,56	2,05	0,00
Antioquia	Peñol	188,71	11,27	1,88	0,00
Antioquia	Toledo	68,20	9,58	1,69	0,00
Antioquia	Heliconia	127,64	8,33	1,63	0,00
Antioquia	La Pintada	261,59	29,83	1,52	0,00
Antioquia	Venecia	234,93	23,26	1,50	0,75
Antioquia	Girardota	234,84	20,43	1,39	0,81
Antioquia	Puerto Berrío	133,16	16,44	1,30	0,70
Antioquia	Dabeiba	58,86	4,23	1,28	0,85
Antioquia	Santafé de Antioquia	243,67	25,96	1,26	0,85
Antioquia	Yalí	111,92	8,58	1,25	1,22
Antioquia	Sabanalarga	73,25	8,55	1,22	2,44
Antioquia	Uramita	43,60	7,26	1,21	0,00
Antioquia	San Roque	215,16	15,32	1,13	0,00
Antioquia	Campamento	50,09	3,30	1,09	0,00
Antioquia	Puerto Nare	58,62	10,22	1,07	0,00
Antioquia	Entrerrios	108,31	12,73	1,04	0,00
Antioquia	Mutatá	78,23	10,07	0,98	4,09
Antioquia	San Luis	152,22	10,03	0,91	0,00

Antioquia	El Bagre	65,63	7,01	0,83	0,63
Antioquia	Barbosa	277,66	13,85	0,81	0,83
Antioquia	Tarazá	27,06	3,34	0,79	0,81
Antioquia	San Rafael	270,28	32,00	0,75	0,76
Antioquia	Remedios	110,81	8,48	0,73	3,05
Antioquia	Ciudad Bolívar	98,15	10,19	0,72	1,43
Antioquia	Chigorodó	98,23	12,19	0,70	1,27
Antioquia	Támesis	294,02	22,33	0,65	1,28
Antioquia	San Pedro de Uraba	68,07	7,20	0,65	5,82
Antioquia	Yondó	90,49	16,45	0,62	6,22
Antioquia	Caldas	153,88	11,83	0,54	0,00
Antioquia	La Unión	167,37	7,96	0,54	0,52
Antioquia	Segovia	61,07	6,23	0,53	0,26
Antioquia	Puerto Triunfo	87,43	14,15	0,52	0,55
Antioquia	Arboletes	109,62	5,77	0,51	3,94
Antioquia	Fredonia	166,98	12,27	0,46	0,45
Antioquia	Nechí	36,65	3,83	0,43	0,40
Antioquia	San Juan de Urabá	47,57	5,49	0,40	2,49
Antioquia	San Pedro	233,38	2,66	0,40	0,74
Antioquia	La Ceja	181,83	11,42	0,38	0,98
Antioquia	El Santuario	116,58	4,07	0,37	1,11
Antioquia	Envigado	77,26	9,57	0,37	0,22
Antioquia	Apartadó	59,83	5,82	0,35	0,31
Antioquia	Zaragoza	91,26	7,83	0,33	2,33
Antioquia	Caucasia	42,48	4,16	0,28	1,03
Antioquia	Urrao	95,70	6,83	0,25	0,24
Antioquia	Bello	87,64	9,26	0,23	0,16
Antioquia	Marinilla	123,01	3,48	0,21	0,00
Antioquia	Sabaneta	118,43	13,93	0,20	0,21
Antioquia	Medellín	86,87	8,73	0,20	0,11
Antioquia	Carepa	70,00	6,03	0,17	2,55
Antioquia	Necoclí	89,71	4,45	0,16	2,65
Antioquia	La Estrella	121,13	10,48	0,16	0,51
Antioquia	Copacabana	133,21	11,03	0,15	0,14
Antioquia	Itagui	91,62	12,31	0,12	0,08
Antioquia	Rionegro	135,61	10,51	0,08	0,34
Antioquia	Abejorral	97,78	11,29	0,00	2,00
Antioquia	Abriaquí	111,60	9,40	0,00	0,00
Antioquia	Alejandría	73,89	17,17	0,00	0,00
Antioquia	Amagá	171,41	15,43	0,00	0,00
Antioquia	Amalfi	81,47	6,91	0,00	0,00

Antioquia	Andes	130,14	9,56	0,00	0,23
Antioquia	Angelópolis	63,47	2,41	0,00	1,17
Antioquia	Angostura	89,18	3,48	0,00	0,00
Antioquia	Anorí	80,80	6,68	0,00	0,63
Antioquia	Anza	49,09	3,97	0,00	0,00
Antioquia	Argelia	279,42	3,31	0,00	0,00
Antioquia	Armenia	177,76	29,17	0,00	0,00
Antioquia	Belmira	56,25	6,11	0,00	3,02
Antioquia	Betania	127,35	6,20	0,00	0,00
Antioquia	Betulia	89,19	8,08	0,00	0,00
Antioquia	Briceño	64,71	4,60	0,00	0,00
Antioquia	Buriticá	91,70	2,98	0,00	0,00
Antioquia	Cáceres	27,29	2,27	0,00	1,19
Antioquia	Caicedo	75,37	3,70	0,00	0,00
Antioquia	Cañasgordas	106,02	5,95	0,00	0,59
Antioquia	Caracolí	194,55	36,44	0,00	4,27
Antioquia	Caramanta	135,27	11,16	0,00	0,00
Antioquia	Cocorná	30,00	0,67	0,00	0,00
Antioquia	Concepción	167,30	21,83	0,00	2,53
Antioquia	Concordia	118,43	9,13	0,00	0,00
Antioquia	Don Matías	82,95	7,98	0,00	0,00
Antioquia	Ebéjico	129,42	11,19	0,00	0,00
Antioquia	El Carmen de Viboral	181,79	11,98	0,00	0,67
Antioquia	Frontino	113,52	7,49	0,00	0,00
Antioquia	Giraldo	61,63	7,37	0,00	2,44
Antioquia	Gómez Plata	61,05	1,55	0,00	0,00
Antioquia	Granada	249,25	2,03	0,00	0,00
Antioquia	Guadalupe	183,19	14,34	0,00	0,00
Antioquia	Guarne	148,13	10,51	0,00	0,45
Antioquia	Ituango	76,56	2,76	0,00	0,00
Antioquia	Jardín	187,35	11,51	0,00	0,00
Antioquia	Jericó	99,13	25,28	0,00	0,00
Antioquia	Maceo	177,10	13,72	0,00	1,35
Antioquia	Montebello	48,91	1,49	0,00	0,00
Antioquia	Murindó	13,40	0,00	0,00	0,00
Antioquia	Olaya	93,97	21,79	0,00	0,00
Antioquia	Peque	66,36	4,69	0,00	0,92
Antioquia	Pueblorrico	231,92	13,60	0,00	1,38
Antioquia	Retiro	187,45	16,38	0,00	1,04
Antioquia	Salgar	130,57	4,51	0,00	0,57
Antioquia	San Andrés de Cuerquía	116,86	9,45	0,00	0,00

Antioquia	San Carlos	68,20	10,60	0,00	0,00
Antioquia	San Francisco	377,72	10,88	0,00	0,00
Antioquia	San Jerónimo	316,00	43,78	0,00	0,00
Antioquia	San José de La Montaña	168,59	15,09	0,00	3,00
Antioquia	San Vicente	48,54	3,51	0,00	0,00
Antioquia	Santa Bárbara	174,40	20,85	0,00	0,44
Antioquia	Santa Rosa de Osos	83,42	7,41	0,00	0,86
Antioquia	Santo Domingo	116,76	12,24	0,00	0,00
Antioquia	Sonson	150,90	10,73	0,00	0,28
Antioquia	Sopetrán	177,86	22,78	0,00	0,00
Antioquia	Tarso	198,04	27,52	0,00	0,00
Antioquia	Titiribí	102,97	16,90	0,00	0,70
Antioquia	Turbo	50,79	3,12	0,00	1,75
Antioquia	Valdivia	77,22	5,98	0,00	0,00
Antioquia	Valparaíso	269,82	30,51	0,00	1,60
Antioquia	Vegachí	105,33	13,33	0,00	0,00
Antioquia	Vigía del Fuerte	3,58	0,00	0,00	0,00
Antioquia	Yarumal	105,49	7,08	0,00	0,21
Antioquia	Yolombó	101,68	8,27	0,00	0,00
Arauca	Tame	155,39	14,00	3,82	29,31
Arauca	Fortul	105,52	10,04	3,53	6,44
Arauca	Saravena	170,04	20,33	3,19	3,92
Arauca	Cravo Norte	168,00	5,81	3,04	49,92
Arauca	Arauca	118,99	12,87	1,91	6,32
Arauca	Arauquita	88,03	6,57	1,00	7,30
Arauca	Puerto Rondón	209,36	18,20	0,00	36,17
Archipiélago de San Andrés	Providencia	38,22	2,91	0,00	0,00
Atlántico	Tubará	145,25	9,09	1,82	5,49
Atlántico	Santo Tomás	149,59	11,88	1,19	0,40
Atlántico	Suan	194,93	13,55	1,13	0,00
Atlántico	Santa Lucía	161,13	6,00	0,84	4,25
Atlántico	Luruaco	127,05	5,62	0,77	2,24
Atlántico	Puerto Colombia	182,27	26,52	0,73	0,00
Atlántico	Juan de Acosta	70,47	6,77	0,62	15,29
Atlántico	Ponedera	128,26	7,82	0,49	0,90
Atlántico	Palmar de Varela	146,28	3,19	0,39	1,20
Atlántico	Sabanagrande	123,95	10,07	0,34	0,64
Atlántico	Baranoa	99,57	7,48	0,18	0,34
Atlántico	Barranquilla	85,91	5,39	0,12	0,30
Atlántico	Sabanalarga	218,27	3,23	0,10	1,93
Atlántico	Malambo	60,83	5,96	0,09	0,34

Atlántico	Soledad	47,22	4,49	0,03	0,22
Atlántico	Campo de La Cruz	130,85	1,25	0,00	1,75
Atlántico	Candelaria	168,10	2,44	0,00	0,82
Atlántico	Galapa	111,84	3,96	0,00	0,00
Atlántico	Manatí	109,65	1,96	0,00	0,63
Atlántico	Piojó	93,58	7,80	0,00	7,85
Atlántico	Polonuevo	168,80	2,65	0,00	0,00
Atlántico	Repelón	99,40	3,06	0,00	0,39
Atlántico	Usiacurí	125,01	9,61	0,00	4,27
Bogotá, D.C.	Bogotá D.C.	108,21	17,49	0,17	0,13
Bolívar	Santa Rosa	269,88	10,03	2,11	2,27
Bolívar	San Juan Nepomuceno	140,42	12,01	1,80	13,54
Bolívar	Magangué	162,44	12,24	1,63	1,05
Bolívar	San Cristóbal	219,10	16,49	1,51	4,50
Bolívar	Calamar	153,16	1,83	1,38	0,86
Bolívar	Cantagallo	105,76	6,52	1,24	0,00
Bolívar	Mompós	125,93	15,83	1,17	1,14
Bolívar	El Carmen de Bolívar	107,28	4,10	1,14	1,94
Bolívar	Villanueva	275,55	11,38	1,11	5,61
Bolívar	Morales	122,86	0,51	1,04	5,58
Bolívar	Arjona	108,00	2,41	0,94	0,87
Bolívar	Talaigua Nuevo	211,37	4,43	0,89	0,00
Bolívar	Zambrano	126,45	2,62	0,88	0,87
Bolívar	Clemencia	144,93	4,02	0,85	3,28
Bolívar	San Fernando	32,58	0,75	0,73	0,73
Bolívar	San Martín de Loba	125,49	9,13	0,66	3,01
Bolívar	Turbaco	121,58	7,26	0,62	1,00
Bolívar	Santa Rosa del Sur	63,94	4,86	0,50	25,72
Bolívar	Achí	121,16	7,01	0,49	0,00
Bolívar	Pinillos	14,95	0,81	0,43	0,42
Bolívar	María La Baja	53,26	1,29	0,22	1,26
Bolívar	Cartagena	28,92	3,07	0,15	0,07
Bolívar	Altos del Rosario	59,12	3,70	0,00	0,00
Bolívar	Arenal	29,37	0,54	0,00	2,61
Bolívar	Arroyohondo	47,57	5,03	0,00	3,12
Bolívar	Barranco de Loba	75,09	5,15	0,00	0,63
Bolívar	Cicuco	196,41	17,11	0,00	0,00
Bolívar	Córdoba	251,74	2,35	0,00	1,58
Bolívar	El Guamo	168,75	5,15	0,00	3,87
Bolívar	El Peñón	138,75	3,41	0,00	4,35
Bolívar	Hatillo de Loba	77,60	3,36	0,00	0,00

Bolívar	Mahates	45,93	1,63	0,00	0,78
Bolívar	Margarita	29,48	3,08	0,00	1,06
Bolívar	Montecristo	7,39	0,00	0,00	5,30
Bolívar	Norosí	13,39	0,00	0,00	0,00
Bolívar	Regidor	19,96	0,99	0,00	0,00
Bolívar	Río Viejo	20,77	1,72	0,00	0,00
Bolívar	San Estanislao	43,39	0,62	0,00	1,23
Bolívar	San Jacinto	86,86	8,36	0,00	2,79
Bolívar	San Jacinto del Cauca	27,68	2,22	0,00	0,76
Bolívar	San Pablo	71,46	6,51	0,00	0,35
Bolívar	Santa Catalina	116,72	4,58	0,00	8,12
Bolívar	Simití	54,91	10,60	0,00	2,56
Bolívar	Soplaviento	60,81	3,58	0,00	0,00
Bolívar	Tiquisio	35,45	1,88	0,00	0,00
Bolívar	Turbaná	76,82	0,69	0,00	2,75
Boyacá	Cubará	46,20	1,49	10,42	32,97
Boyacá	Pisba	95,30	7,33	7,05	0,00
Boyacá	Boavita	215,10	27,23	4,74	2,65
Boyacá	Pajarito	119,50	22,87	4,44	5,99
Boyacá	Santana	260,24	24,62	2,60	1,30
Boyacá	Muzo	154,78	16,11	2,23	1,12
Boyacá	Miraflores	154,67	21,50	2,05	1,02
Boyacá	Boyacá	283,93	30,27	2,02	2,05
Boyacá	Togüí	161,60	23,81	1,97	2,03
Boyacá	Labranzagrande	65,58	7,76	1,93	9,55
Boyacá	Chitaraque	213,68	35,81	1,76	1,79
Boyacá	Puerto Boyacá	79,88	26,56	1,45	3,13
Boyacá	Motavita	135,69	10,14	1,37	0,00
Boyacá	Maripí	80,97	6,63	1,28	2,69
Boyacá	Quípama	62,91	3,75	1,27	0,00
Boyacá	Soatá	303,88	66,74	1,13	1,38
Boyacá	Tuta	120,02	16,66	1,05	1,03
Boyacá	Umbita	125,24	12,62	0,98	0,00
Boyacá	Otanche	106,30	20,67	0,95	0,00
Boyacá	Moniquirá	287,87	51,19	0,93	1,39
Boyacá	Ráquira	118,66	8,95	0,73	2,24
Boyacá	Villa de Leyva	397,90	29,63	0,69	0,00
Boyacá	Garagoa	147,92	19,52	0,60	0,60
Boyacá	Chiquinquirá	128,74	9,77	0,15	0,30
Boyacá	Almeida	230,91	15,93	0,00	0,00
Boyacá	Aquitania	248,55	26,77	0,00	1,91

Boyacá	Arcabuco	189,08	13,37	0,00	1,91
Boyacá	Belén	183,93	25,45	0,00	5,40
Boyacá	Berbeo	145,21	31,10	0,00	0,00
Boyacá	Betéitiva	81,19	9,10	0,00	8,61
Boyacá	Briceño	61,95	7,52	0,00	0,00
Boyacá	Buenavista	131,85	13,76	0,00	0,00
Boyacá	Busbanzá	146,01	0,00	0,00	0,00
Boyacá	Caldas	138,39	16,27	0,00	5,50
Boyacá	Campohermoso	102,55	18,18	0,00	12,55
Boyacá	Cerinza	161,67	31,25	0,00	5,12
Boyacá	Chinavita	199,00	39,12	0,00	8,42
Boyacá	Chíquiza	112,39	7,03	0,00	0,00
Boyacá	Chiscas	175,98	13,45	0,00	0,00
Boyacá	Chita	183,19	18,36	0,00	4,10
Boyacá	Chivatá	118,51	11,80	0,00	3,30
Boyacá	Chivor	101,81	10,53	0,00	0,00
Boyacá	Ciénega	104,65	4,14	0,00	2,13
Boyacá	Cómbita	129,31	13,87	0,00	0,72
Boyacá	Coper	183,83	13,32	0,00	0,00
Boyacá	Corrales	223,74	8,59	0,00	0,00
Boyacá	Covarachía	187,60	37,88	0,00	0,00
Boyacá	Cucaita	134,90	12,86	0,00	2,15
Boyacá	Cuítiva	314,09	10,40	0,00	5,29
Boyacá	Duitama	197,84	29,31	0,00	0,36
Boyacá	El Cocuy	130,32	16,83	0,00	0,00
Boyacá	El Espino	112,79	43,09	0,00	2,38
Boyacá	Firavitoba	162,93	15,04	0,00	0,00
Boyacá	Floresta	154,78	17,33	0,00	0,00
Boyacá	Gachantivá	317,70	29,80	0,00	7,47
Boyacá	Gameza	228,45	17,85	0,00	1,99
Boyacá	Guacamayas	217,44	16,43	0,00	0,00
Boyacá	Guateque	234,79	22,74	0,00	0,00
Boyacá	Guayatá	203,57	9,41	0,00	1,91
Boyacá	Güicán	61,77	2,75	0,00	0,00
Boyacá	Iza	178,20	12,93	0,00	0,00
Boyacá	Jenesano	181,27	14,44	0,00	0,00
Boyacá	Jericó	240,26	4,93	0,00	0,00
Boyacá	La Capilla	166,99	30,02	0,00	7,45
Boyacá	La Uvita	171,08	12,03	0,00	0,00
Boyacá	La Victoria	0,00	0,00	0,00	0,00
Boyacá	Macanal	187,21	12,50	0,00	2,07

Boyacá	Mongua	116,20	10,29	0,00	0,00
Boyacá	Monguí	164,40	28,07	0,00	2,01
Boyacá	Nobsa	243,02	37,12	0,00	0,62
Boyacá	Nuevo Colón	168,41	15,49	0,00	1,55
Boyacá	Oicatá	293,00	10,59	0,00	14,12
Boyacá	Pachavita	259,49	19,28	0,00	0,00
Boyacá	Páez	179,46	6,92	0,00	3,43
Boyacá	Paipa	270,35	25,76	0,00	1,01
Boyacá	Panqueba	123,04	63,97	0,00	0,00
Boyacá	Pauna	106,31	5,61	0,00	0,94
Boyacá	Paya	81,58	11,76	0,00	7,77
Boyacá	Paz de Río	233,50	46,07	0,00	0,00
Boyacá	Pesca	216,88	17,77	0,00	1,27
Boyacá	Ramiriquí	212,68	22,72	0,00	4,78
Boyacá	Rondón	98,98	0,00	0,00	0,00
Boyacá	Saboyá	128,36	8,05	0,00	0,80
Boyacá	Sáchica	326,08	18,42	0,00	2,65
Boyacá	Samacá	187,25	10,73	0,00	0,00
Boyacá	San Eduardo	212,76	10,67	0,00	0,00
Boyacá	San José de Pare	210,52	20,89	0,00	1,80
Boyacá	San Luis de Gaceno	103,36	20,79	0,00	3,95
Boyacá	San Mateo	288,54	51,75	0,00	0,00
Boyacá	San Miguel de Sema	120,52	15,34	0,00	2,19
Boyacá	San Pablo de Borbur	56,39	0,95	0,00	0,00
Boyacá	Santa María	140,41	14,81	0,00	79,12
Boyacá	Santa Rosa de Viterbo	182,90	26,13	0,00	5,23
Boyacá	Santa Sofía	233,17	21,59	0,00	0,00
Boyacá	Sativanorte	157,56	20,79	0,00	0,00
Boyacá	Sativasur	171,05	17,10	0,00	0,00
Boyacá	Siachoque	127,27	14,52	0,00	6,70
Boyacá	Socha	156,08	28,98	0,00	0,00
Boyacá	Socotá	97,85	8,32	0,00	1,12
Boyacá	Sogamoso	309,92	31,18	0,00	0,35
Boyacá	Somondoco	198,97	24,60	0,00	0,00
Boyacá	Sora	142,34	3,31	0,00	0,00
Boyacá	Soracá	227,22	11,24	0,00	1,89
Boyacá	Sotaquirá	110,97	12,98	0,00	0,00
Boyacá	Susacón	141,46	34,33	0,00	5,95
Boyacá	Sutamarchán	161,35	6,69	0,00	0,00
Boyacá	Sutatenza	149,76	24,29	0,00	0,00
Boyacá	Tasco	193,49	18,53	0,00	0,00

Boyacá	Tenza	190,53	26,16	0,00	4,94
Boyacá	Tibaná	238,56	25,83	0,00	1,09
Boyacá	Tibasosa	177,12	21,67	0,00	0,00
Boyacá	Tinjacá	244,60	26,45	0,00	0,00
Boyacá	Tipacoque	169,62	30,32	0,00	6,05
Boyacá	Toca	83,49	2,92	0,00	0,99
Boyacá	Tópaga	216,58	5,41	0,00	0,00
Boyacá	Tota	167,37	9,06	0,00	1,82
Boyacá	Tunja	184,66	14,69	0,00	0,32
Boyacá	Tununguá	99,88	16,30	0,00	5,92
Boyacá	Turmequé	145,12	39,21	0,00	0,00
Boyacá	Tutazá	165,14	21,18	0,00	0,00
Boyacá	Ventaquemada	149,56	4,61	0,00	0,65
Boyacá	Viracachá	226,04	30,52	0,00	3,05
Boyacá	Zetaquira	157,95	12,95	0,00	0,00
Caldas	Norcasia	228,59	32,19	6,15	1,48
Caldas	Viterbo	139,37	29,94	4,76	3,17
Caldas	Victoria	327,54	53,46	3,45	4,73
Caldas	Marulanda	29,22	8,76	2,90	0,00
Caldas	Palestina	328,91	28,57	2,78	1,12
Caldas	Marquetalia	143,14	15,38	2,01	0,00
Caldas	Chinchiná	330,84	32,80	1,70	0,77
Caldas	Aranzazu	204,91	20,53	1,66	2,66
Caldas	Samaná	132,84	25,26	1,55	2,33
Caldas	San José	191,15	27,68	1,32	0,00
Caldas	Pensilvania	120,94	17,44	1,14	1,14
Caldas	Neira	111,32	13,37	1,03	0,68
Caldas	Risaralda	183,11	17,27	0,96	0,00
Caldas	Riosucio	168,97	15,00	0,88	0,33
Caldas	Anserma	141,93	19,67	0,87	0,00
Caldas	Belalcázar	338,35	37,11	0,86	1,82
Caldas	La Dorada	97,84	21,30	0,79	0,91
Caldas	Supía	88,63	15,15	0,77	0,75
Caldas	Manizales	215,01	25,75	0,34	0,49
Caldas	Villamaría	204,37	19,78	0,21	0,94
Caldas	Aguadas	131,19	26,17	0,00	0,42
Caldas	Filadelfia	155,64	16,63	0,00	0,91
Caldas	La Merced	186,36	36,58	0,00	0,00
Caldas	Manzanares	120,83	13,92	0,00	0,84
Caldas	Marmato	160,88	12,45	0,00	0,00
Caldas	Pácora	280,57	28,10	0,00	0,00

Caldas	Salamina	138,72	25,78	0,00	1,12
Caquetá	Albania	79,63	4,67	1,56	0,00
Caquetá	San Vicente del Caguán	66,82	4,18	0,89	0,59
Caquetá	Florencia	332,17	19,90	0,81	0,36
Caquetá	San José del Fragua	92,84	8,15	0,68	4,79
Caquetá	El Paujil	93,26	12,25	0,55	2,97
Caquetá	La Montañita	23,09	2,97	0,42	3,36
Caquetá	Solano	19,02	1,28	0,41	0,00
Caquetá	Belén de los Andaquies	61,15	9,61	0,00	7,93
Caquetá	Cartagena del Chairá	38,32	3,66	0,00	1,49
Caquetá	Curillo	65,72	12,90	0,00	6,84
Caquetá	El Doncello	79,83	11,79	0,00	0,45
Caquetá	Milán	29,18	1,71	0,00	0,00
Caquetá	Morelia	70,94	7,90	0,00	2,61
Caquetá	Puerto Rico	19,30	5,99	0,00	4,23
Caquetá	Solita	66,71	5,47	0,00	1,09
Caquetá	Valparaíso	47,02	5,21	0,00	0,00
Casanare	San Luis de Palenque	171,35	16,96	23,65	54,46
Casanare	Nunchía	198,42	16,03	15,02	94,34
Casanare	Orocué	162,14	28,23	12,28	38,55
Casanare	Trinidad	144,74	20,74	11,92	26,98
Casanare	Hato Corozal	111,82	5,98	7,84	32,51
Casanare	Monterrey	223,58	34,09	5,88	8,56
Casanare	Támara	174,34	8,50	5,67	7,08
Casanare	Maní	259,29	25,13	4,48	7,18
Casanare	Paz de Ariporo	165,11	16,86	2,99	39,91
Casanare	Aguazul	253,04	32,30	2,86	5,22
Casanare	Pore	266,22	25,24	2,52	78,16
Casanare	Tauramena	213,25	30,65	2,49	9,83
Casanare	Villanueva	258,92	44,32	2,18	1,32
Casanare	Yopal	160,94	28,32	1,99	9,40
Casanare	Chameza	96,96	4,07	0,00	0,00
Casanare	La Salina	65,15	6,97	0,00	0,00
Casanare	Recetor	11,59	0,00	0,00	37,90
Casanare	Sabanalarga	55,62	73,74	0,00	6,15
Casanare	Sácama	192,04	9,83	0,00	15,20
Cauca	Guapi	88,29	6,75	6,07	3,40
Cauca	Florencia	121,21	32,70	3,27	4,90
Cauca	Villa Rica	379,60	36,52	1,99	1,90
Cauca	Timbiquí	26,67	0,46	1,86	0,47
Cauca	Piamonte	78,15	17,88	1,37	0,00

Cauca	Santander de Quilichao	356,92	72,60	1,35	4,27
Cauca	Caloto	352,75	34,75	1,14	3,41
Cauca	Toribio	225,92	10,96	1,10	2,84
Cauca	La Sierra	314,41	17,76	0,93	2,81
Cauca	Patía	217,62	41,44	0,85	2,00
Cauca	Rosas	361,66	41,24	0,76	11,64
Cauca	San Sebastián	158,31	16,15	0,73	1,47
Cauca	Mercaderes	153,32	28,89	0,56	0,56
Cauca	Miranda	186,22	26,37	0,51	0,54
Cauca	López	26,40	0,00	0,50	0,00
Cauca	Popayán	255,04	28,00	0,44	1,72
Cauca	Silvia	171,31	18,21	0,31	2,81
Cauca	Timbío	410,16	44,36	0,30	7,29
Cauca	Piendamó	277,76	31,92	0,23	1,26
Cauca	La Vega	114,15	7,89	0,22	15,83
Cauca	Puerto Tejada	216,57	21,87	0,22	1,99
Cauca	El Tambo	116,57	8,90	0,21	4,90
Cauca	Almaguer	119,84	9,01	0,00	5,25
Cauca	Argelia	96,38	6,43	0,00	0,40
Cauca	Balboa	128,24	11,13	0,00	0,00
Cauca	Bolívar	76,64	13,48	0,00	8,07
Cauca	Buenos Aires	123,38	15,94	0,00	0,95
Cauca	Cajibío	149,10	9,94	0,00	2,66
Cauca	Caldono	103,00	12,19	0,00	8,27
Cauca	Corinto	178,65	24,28	0,00	1,62
Cauca	Guachené	166,37	6,07	0,00	0,50
Cauca	Inzá	146,48	9,31	0,00	0,66
Cauca	Jambaló	96,45	8,28	0,00	1,15
Cauca	Morales	16,84	9,31	0,00	11,45
Cauca	Padilla	397,89	27,50	0,00	1,26
Cauca	Paez	89,13	7,55	0,00	0,88
Cauca	Puracé	267,80	17,71	0,00	0,66
Cauca	Santa Rosa	58,53	4,89	0,00	3,03
Cauca	Sotara	128,20	14,48	0,00	0,00
Cauca	Suárez	105,57	23,99	0,00	18,05
Cauca	Sucre	38,43	19,11	0,00	3,37
Cauca	Totoró	169,80	10,56	0,00	2,99
Cesar	Pailitas	164,89	10,58	1,18	0,58
Cesar	San Martín	157,59	13,14	1,12	8,34
Cesar	El Paso	27,32	1,31	0,90	11,53
Cesar	El Copey	151,12	5,35	0,76	14,87

Cesar	Becerril	162,97	5,92	0,74	1,47
Cesar	Gamarra	24,59	2,53	0,66	0,63
Cesar	Chimichagua	88,17	1,31	0,65	0,98
Cesar	Aguachica	54,00	5,79	0,57	1,96
Cesar	Pelaya	129,47	7,33	0,57	2,26
Cesar	Astrea	66,25	2,63	0,52	3,65
Cesar	San Alberto	81,29	9,25	0,48	0,41
Cesar	Chiriguaná	92,00	4,51	0,46	0,00
Cesar	Pueblo Bello	50,76	1,37	0,46	0,44
Cesar	La Paz	91,20	3,51	0,44	4,46
Cesar	Curumaní	140,60	9,27	0,40	3,13
Cesar	Valledupar	37,65	3,17	0,27	0,75
Cesar	Agustín Codazzi	68,10	3,31	0,19	5,49
Cesar	Bosconia	10,35	0,00	0,00	1,69
Cesar	González	74,79	20,50	0,00	0,00
Cesar	La Gloria	85,50	7,66	0,00	1,53
Cesar	La Jagua de Ibirico	152,43	5,39	0,00	2,70
Cesar	Manaure	8,00	4,89	0,00	0,00
Cesar	Río de Oro	109,22	9,24	0,00	11,94
Cesar	San Diego	75,27	2,99	0,00	17,78
Cesar	Tamalameque	69,11	5,05	0,00	2,88
Choco	Riosucio	1,40	0,00	0,00	0,00
Chocó	Nuquí	2,44	0,00	12,92	25,01
Chocó	Medio Baudó	0,00	0,00	8,85	0,00
Chocó	El Litoral del San Juan	1,42	0,00	1,42	0,00
Chocó	Bajo Baudó	2,31	0,00	1,17	29,79
Chocó	Acandí	6,05	0,00	1,03	1,02
Chocó	Unguía	2,00	0,00	0,66	0,00
Chocó	Medio Atrato	0,00	0,35	0,36	0,00
Chocó	Alto Baudo	1,17	0,00	0,30	7,98
Chocó	Quibdó	13,87	1,30	0,09	0,17
Chocó	Atrato	6,13	1,06	0,00	0,00
Chocó	Bagadó	3,71	1,24	0,00	0,00
Chocó	Bahía Solano	16,14	0,00	0,00	0,00
Chocó	Belén de Bajira	0,00	0,00	0,00	0,00
Chocó	Bojaya	0,99	0,99	0,00	19,99
Chocó	Carmen del Darien	10,96	0,00	0,00	0,00
Chocó	Cértegui	1,01	0,00	0,00	0,00
Chocó	Condoto	28,69	2,89	0,00	0,00
Chocó	El Cantón del San Pablo	4,06	1,35	0,00	1,35
Chocó	El Carmen de Atrato	32,07	5,38	0,00	0,00

Chocó	Istmina	2,01	0,00	0,00	0,00
Chocó	Juradó	11,74	2,94	0,00	0,00
Chocó	Lloró	1,87	0,00	0,00	0,00
Chocó	Medio San Juan	7,98	0,70	0,00	15,61
Chocó	Nóvita	6,31	0,00	0,00	0,00
Chocó	Río Iro	2,30	0,00	0,00	1,15
Chocó	Río Quito	7,11	0,00	0,00	0,00
Chocó	San José del Palmar	26,45	8,17	0,00	6,16
Chocó	Sipí	0,00	0,00	0,00	0,00
Chocó	Tadó	3,74	0,00	0,00	0,00
Chocó	Unión Panamericana	1,11	1,11	0,00	0,00
Córdoba	Chinú	112,51	7,21	2,37	2,92
Córdoba	Lorica	121,43	8,66	1,73	5,78
Córdoba	Sahagún	156,42	9,29	1,46	6,82
Córdoba	Chimá	22,30	0,00	1,38	1,36
Córdoba	Montería	105,27	11,16	1,10	1,44
Córdoba	San Antero	137,60	8,90	1,04	4,27
Córdoba	Buenavista	177,48	6,73	0,95	2,45
Córdoba	San José de Uré	11,97	1,82	0,90	0,00
Córdoba	Montelíbano	60,25	8,54	0,76	0,13
Córdoba	Cotorra	118,85	6,51	0,66	1,30
Córdoba	Ciénaga de Oro	109,09	6,02	0,63	2,86
Córdoba	Planeta Rica	18,39	1,36	0,61	0,89
Córdoba	Tuchín	55,14	0,52	0,57	3,94
Córdoba	Tierralta	44,17	3,96	0,53	1,43
Córdoba	Los Córdobas	74,37	2,54	0,46	5,65
Córdoba	Moñitos	21,97	0,36	0,36	1,86
Córdoba	San Bernardo del Viento	129,24	5,89	0,29	4,05
Córdoba	Pueblo Nuevo	44,27	1,10	0,28	0,81
Córdoba	San Andrés Sotavento	93,38	2,77	0,27	5,09
Córdoba	San Pelayo	23,48	1,43	0,24	0,92
Córdoba	Valencia	89,21	3,20	0,23	1,22
Córdoba	Ayapel	45,47	3,37	0,00	0,59
Córdoba	Canalete	59,05	2,00	0,00	8,27
Córdoba	Cereté	111,66	7,30	0,00	0,88
Córdoba	La Apartada	24,73	2,10	0,00	0,00
Córdoba	Momil	141,51	12,24	0,00	18,39
Córdoba	Puerto Escondido	38,90	4,12	0,00	3,47
Córdoba	Puerto Libertador	46,70	3,45	0,00	0,68
Córdoba	Purísima	89,88	5,32	0,00	3,33
Córdoba	San Carlos	102,34	3,41	0,00	7,78

Cundinamarca	Paratebueno	198,29	32,67	6,54	7,84
Cundinamarca	Jerusalén	208,29	26,04	3,73	3,73
Cundinamarca	Ricaurte	283,40	39,07	3,22	1,12
Cundinamarca	Villeta	319,32	47,67	2,81	0,40
Cundinamarca	Agua de Dios	267,42	50,83	2,74	0,00
Cundinamarca	Apulo	328,93	65,27	2,56	5,12
Cundinamarca	Gama	128,67	27,72	2,49	0,00
Cundinamarca	San Antonio del Tequendama	232,16	17,72	2,32	0,76
Cundinamarca	Topaipí	21,96	4,41	2,13	0,00
Cundinamarca	Quebradanegra	112,19	8,46	2,12	0,00
Cundinamarca	Supatá	169,42	19,91	2,00	0,00
Cundinamarca	Medina	102,45	14,91	2,00	1,99
Cundinamarca	Zipacón	251,10	27,49	1,83	1,78
Cundinamarca	Gachala	163,16	6,95	1,75	1,74
Cundinamarca	Guachetá	304,86	7,88	1,74	7,89
Cundinamarca	Arbeláez	194,50	27,08	1,68	0,82
Cundinamarca	Fomeque	291,98	18,88	1,64	0,00
Cundinamarca	Quetame	138,28	18,61	1,48	66,06
Cundinamarca	La Vega	213,53	35,55	1,41	0,00
Cundinamarca	Granada	97,94	19,42	1,15	0,00
Cundinamarca	San Francisco	93,74	40,98	1,09	2,20
Cundinamarca	San Juan de Río Seco	148,64	16,52	1,03	1,03
Cundinamarca	La Mesa	240,14	34,20	0,98	1,30
Cundinamarca	El Colegio	243,78	58,04	0,94	1,37
Cundinamarca	Silvania	363,61	31,48	0,92	0,91
Cundinamarca	Choachí	249,09	23,01	0,91	2,77
Cundinamarca	Guaduas	142,70	24,35	0,83	1,65
Cundinamarca	Girardot	209,74	39,37	0,77	0,69
Cundinamarca	Viotá	318,54	57,58	0,75	2,99
Cundinamarca	Nilo	111,19	16,12	0,59	0,56
Cundinamarca	Tenjo	301,48	28,52	0,52	3,03
Cundinamarca	Tabio	237,78	19,13	0,45	1,12
Cundinamarca	Sibaté	199,84	16,11	0,30	0,28
Cundinamarca	Fusagasugá	176,38	22,44	0,23	0,39
Cundinamarca	Madrid	213,22	16,45	0,13	0,66
Cundinamarca	Soacha	120,42	15,29	0,07	0,32
Cundinamarca	Albán	144,41	10,08	0,00	1,68
Cundinamarca	Anapoima	231,94	27,00	0,00	0,75
Cundinamarca	Anolaima	267,31	26,11	0,00	1,61
Cundinamarca	Beltrán	42,26	14,19	0,00	0,00
Cundinamarca	Bituima	109,78	11,90	0,00	0,00

Cundinamarca	Bojacá	234,70	16,85	0,00	0,91
Cundinamarca	Cabrera	105,27	13,14	0,00	0,00
Cundinamarca	Cachipay	148,12	14,21	0,00	0,00
Cundinamarca	Cajicá	261,17	23,22	0,00	1,26
Cundinamarca	Caparrapí	45,62	6,60	0,00	0,00
Cundinamarca	Caqueza	270,94	32,02	0,00	1,77
Cundinamarca	Carmen de Carupa	137,12	4,44	0,00	2,23
Cundinamarca	Chaguaní	127,60	17,56	0,00	2,51
Cundinamarca	Chía	178,86	17,69	0,00	0,74
Cundinamarca	Chipaque	265,49	25,00	0,00	0,00
Cundinamarca	Chocontá	121,57	6,07	0,00	0,84
Cundinamarca	Cogua	141,44	11,53	0,00	0,98
Cundinamarca	Cota	367,38	32,60	0,00	0,79
Cundinamarca	Cucunubá	216,59	5,43	0,00	2,75
Cundinamarca	El Peñón	71,85	6,21	0,00	0,00
Cundinamarca	El Rosal	325,12	18,67	0,00	3,56
Cundinamarca	Facatativá	226,31	18,41	0,00	0,33
Cundinamarca	Fosca	110,25	11,17	0,00	0,00
Cundinamarca	Funza	166,31	25,68	0,00	0,00
Cundinamarca	Fúquene	179,25	10,89	0,00	5,34
Cundinamarca	Gachancipá	223,80	11,92	0,00	0,75
Cundinamarca	Gachetá	193,93	13,63	0,00	0,91
Cundinamarca	Guasca	390,12	36,57	0,00	2,09
Cundinamarca	Guataquí	132,01	7,80	0,00	0,00
Cundinamarca	Guatavita	216,00	17,55	0,00	0,00
Cundinamarca	Guayabal de Siquima	195,43	24,76	0,00	0,00
Cundinamarca	Guayabetal	314,99	32,81	0,00	0,00
Cundinamarca	Gutiérrez	165,32	7,75	0,00	2,65
Cundinamarca	Junín	141,75	18,74	0,00	0,00
Cundinamarca	La Calera	345,70	39,23	0,00	1,91
Cundinamarca	La Palma	94,26	9,45	0,00	0,00
Cundinamarca	La Peña	159,64	21,36	0,00	1,42
Cundinamarca	Lenguazaque	108,29	6,82	0,00	1,01
Cundinamarca	Macheta	161,61	12,28	0,00	0,00
Cundinamarca	Manta	224,09	25,54	0,00	4,26
Cundinamarca	Mosquera	153,40	25,76	0,00	0,13
Cundinamarca	Nariño	140,48	32,37	0,00	0,00
Cundinamarca	Nemocón	178,23	15,38	0,00	0,78
Cundinamarca	Nimaima	55,19	8,22	0,00	1,56
Cundinamarca	Nocaima	114,38	10,06	0,00	0,00
Cundinamarca	Pacho	152,76	17,43	0,00	1,11

Cundinamarca	Paime	39,36	8,81	0,00	2,22
Cundinamarca	Pandi	214,22	32,16	0,00	0,00
Cundinamarca	Pasca	126,25	14,13	0,00	0,00
Cundinamarca	Puerto Salgar	113,53	23,57	0,00	0,55
Cundinamarca	Pulí	127,80	6,74	0,00	0,00
Cundinamarca	Quipile	132,15	14,69	0,00	0,00
Cundinamarca	San Bernardo	146,50	20,75	0,00	0,96
Cundinamarca	San Cayetano	46,85	9,36	0,00	0,00
Cundinamarca	Sasaima	380,94	34,06	0,00	0,95
Cundinamarca	Sesquilé	225,73	22,38	0,00	0,82
Cundinamarca	Simijaca	228,39	27,98	0,00	2,34
Cundinamarca	Sopó	210,68	15,72	0,00	0,73
Cundinamarca	Subachoque	207,46	17,51	0,00	1,88
Cundinamarca	Suesca	231,85	13,14	0,00	1,78
Cundinamarca	Susa	67,12	5,10	0,00	1,87
Cundinamarca	Sutatausa	183,12	2,01	0,00	2,01
Cundinamarca	Tausa	184,20	6,92	0,00	1,18
Cundinamarca	Tena	159,36	12,43	0,00	2,27
Cundinamarca	Tibacuy	227,87	16,58	0,00	2,07
Cundinamarca	Tibirita	337,74	23,66	0,00	3,36
Cundinamarca	Tocaima	255,91	43,38	0,00	1,09
Cundinamarca	Tocancipá	172,00	9,54	0,00	1,00
Cundinamarca	Ubalá	127,04	11,96	0,00	4,58
Cundinamarca	Ubaque	332,23	22,03	0,00	3,16
Cundinamarca	Une	149,52	13,71	0,00	1,12
Cundinamarca	Útica	146,32	48,10	0,00	4,01
Cundinamarca	Venecia	190,81	27,12	0,00	2,46
Cundinamarca	Vergara	130,39	18,26	0,00	0,00
Cundinamarca	Vianí	182,29	19,11	0,00	4,76
Cundinamarca	Villa de San Diego de Ubate	208,31	16,14	0,00	2,07
Cundinamarca	Villagómez	64,59	23,06	0,00	0,00
Cundinamarca	Villapinzón	60,24	7,24	0,00	0,00
Cundinamarca	Yacopí	36,77	10,11	0,00	0,60
Cundinamarca	Zipaquirá	127,65	15,10	0,00	0,50
Guainía	Puerto Colombia	2,30	0,00	4,61	0,00
Guainía	Inírida	137,36	28,18	4,11	1,50
Guainía	Barranco Minas	6,28	2,08	0,00	0,00
Guainía	Cacahual	0,00	0,00	0,00	0,00
Guainía	La Guadalupe	0,00	0,00	0,00	0,00
Guainía	Mapiripana	0,00	0,00	0,00	0,00
Guainía	Morichal	0,00	0,00	0,00	0,00

Guainía	Pana Pana	0,00	0,00	0,00	0,00
Guainía	San Felipe	21,13	0,00	0,00	0,00
Guaviare	San José del Guaviare	91,30	15,01	1,15	0,80
Guaviare	Miraflores	56,24	5,02	0,73	0,00
Guaviare	El Retorno	51,43	7,65	0,48	0,96
Guaviare	Calamar	46,41	29,89	0,00	5,06
Huila	Agrado	219,55	18,95	2,29	0,00
Huila	Neiva	216,21	43,51	1,99	0,60
Huila	Paicol	191,32	49,23	1,81	0,00
Huila	Timaná	166,35	15,79	1,49	0,49
Huila	Hobo	103,79	10,20	1,44	0,00
Huila	Teruel	229,16	16,40	1,20	0,00
Huila	Baraya	135,80	17,73	1,04	0,00
Huila	Palestina	120,63	5,30	0,86	0,00
Huila	Tarqui	67,73	5,10	0,57	0,00
Huila	Garzón	94,03	11,58	0,49	0,49
Huila	Pitalito	155,69	10,68	0,44	0,44
Huila	Algeciras	125,13	15,21	0,42	0,82
Huila	Isnos	77,77	1,54	0,41	0,00
Huila	Aipe	113,44	12,29	0,38	0,40
Huila	Acevedo	127,30	7,53	0,36	0,30
Huila	San Agustín	192,24	9,48	0,31	0,61
Huila	Palermo	136,38	15,31	0,31	2,30
Huila	Gigante	60,16	8,83	0,31	0,00
Huila	La Plata	91,80	8,37	0,18	0,17
Huila	Altamira	80,62	2,28	0,00	0,00
Huila	Campoalegre	103,85	8,79	0,00	0,29
Huila	Colombia	96,87	8,13	0,00	0,00
Huila	Elías	135,31	20,55	0,00	0,00
Huila	Guadalupe	100,35	19,58	0,00	0,00
Huila	Iquira	79,42	5,00	0,00	0,00
Huila	La Argentina	104,30	10,55	0,00	0,00
Huila	Nátaga	158,36	11,31	0,00	0,00
Huila	Oporapa	118,08	6,17	0,00	0,00
Huila	Pital	173,02	22,10	0,00	0,00
Huila	Rivera	258,74	27,73	0,00	0,58
Huila	Saladoblanco	174,60	6,14	0,00	1,77
Huila	Santa María	121,80	4,47	0,00	0,00
Huila	Suaza	185,49	13,55	0,00	0,00
Huila	Tello	67,15	8,49	0,00	0,00
Huila	Tesalia	130,07	17,42	0,00	0,00

Huila	Villavieja	91,51	10,92	0,00	0,00
Huila	Yaguará	167,01	36,56	0,00	0,00
La Guajira	Urumita	56,15	1,66	1,26	0,00
La Guajira	San Juan del Cesar	155,95	10,67	1,10	2,20
La Guajira	Maicao	73,55	6,00	0,72	0,65
La Guajira	Barrancas	166,59	9,14	0,70	0,89
La Guajira	Albania	37,34	6,89	0,42	3,11
La Guajira	Dibulla	52,77	1,40	0,29	0,69
La Guajira	Manaure	104,87	0,75	0,09	0,31
La Guajira	Riohacha	81,34	5,89	0,05	0,33
La Guajira	Distracción	22,39	5,26	0,00	0,65
La Guajira	El Molino	158,96	6,15	0,00	2,51
La Guajira	Fonseca	104,77	7,39	0,00	0,60
La Guajira	Hatonuevo	20,14	0,42	0,00	0,42
La Guajira	La Jagua del Pilar	54,76	3,28	0,00	0,00
La Guajira	Uribia	7,09	0,18	0,00	0,24
La Guajira	Villanueva	279,51	5,57	0,00	0,00
Magdalena	Santa Bárbara de Pinto	58,48	4,79	4,30	39,72
Magdalena	Pivijay	103,13	3,52	2,62	7,65
Magdalena	Tenerife	62,79	2,45	2,43	22,74
Magdalena	Zapayán	66,07	5,70	2,26	7,94
Magdalena	Plato	60,78	4,23	2,22	19,40
Magdalena	Fundación	26,34	1,22	2,10	1,57
Magdalena	El Retén	82,08	0,94	1,46	0,95
Magdalena	Salamina	244,11	4,19	1,35	5,14
Magdalena	Pijiño del Carmen	75,40	5,10	1,34	27,61
Magdalena	Nueva Granada	13,71	0,49	1,05	11,51
Magdalena	Algarrobo	84,34	6,43	0,83	48,78
Magdalena	Guamal	204,00	2,96	0,73	0,37
Magdalena	Zona Bananera	73,13	2,32	0,67	0,17
Magdalena	Sabanas de San Angel	58,29	3,56	0,65	37,44
Magdalena	Aracataca	25,76	1,78	0,53	0,79
Magdalena	Santa Marta	82,65	12,17	0,49	0,62
Magdalena	El Banco	56,15	3,25	0,36	1,81
Magdalena	Ariguaní	45,50	3,76	0,31	16,25
Magdalena	Ciénaga	65,99	5,94	0,10	0,96
Magdalena	Cerro San Antonio	130,33	7,51	0,00	1,27
Magdalena	Chivolo	151,13	8,74	0,00	105,01
Magdalena	Concordia	169,97	4,25	0,00	1,05
Magdalena	El Piñon	166,32	7,76	0,00	15,52
Magdalena	Pedraza	53,27	6,20	0,00	1,24

Magdalena	Puebloviejo	93,34	7,26	0,00	0,00
Magdalena	Remolino	40,10	2,42	0,00	2,45
Magdalena	San Sebastián de Buenavista	48,04	5,15	0,00	2,87
Magdalena	San Zenón	189,35	4,40	0,00	0,00
Magdalena	Santa Ana	49,90	3,17	0,00	40,97
Magdalena	Sitionuevo	18,06	0,95	0,00	0,62
Meta	Fuente de Oro	170,00	29,29	13,68	3,23
Meta	San Juanito	81,30	4,75	9,64	0,00
Meta	Barranca de Upía	159,14	41,70	8,28	13,80
Meta	Cabuyaro	181,69	45,05	5,09	31,06
Meta	Castilla la Nueva	283,56	46,12	3,25	0,00
Meta	El Dorado	257,41	26,41	2,93	2,94
Meta	Puerto Gaitán	123,19	17,43	2,69	2,73
Meta	San Carlos de Guaroa	176,01	23,82	2,21	2,61
Meta	Guamal	82,98	25,81	2,15	3,26
Meta	Cubarral	156,84	28,98	1,75	0,00
Meta	Cumaral	195,42	34,09	1,68	3,34
Meta	El Castillo	199,36	15,36	1,56	1,57
Meta	Puerto López	135,45	29,33	1,25	4,84
Meta	San Martín	91,80	27,90	1,24	6,08
Meta	Villavicencio	137,03	29,03	1,02	1,26
Meta	Restrepo	377,98	53,91	0,94	6,62
Meta	Granada	57,92	18,48	0,70	0,18
Meta	Acacías	152,37	32,79	0,62	1,41
Meta	Puerto Concordia	27,75	7,59	0,49	5,61
Meta	Vistahermosa	60,75	10,01	0,45	1,58
Meta	El Calvario	44,20	4,43	0,00	0,00
Meta	La Macarena	34,89	5,88	0,00	2,26
Meta	Lejanías	134,89	13,80	0,00	1,06
Meta	Mapiripán	23,44	3,68	0,00	8,03
Meta	Mesetas	83,89	9,75	0,00	0,00
Meta	Puerto Lleras	83,60	10,04	0,00	32,46
Meta	Puerto Rico	56,45	6,04	0,00	0,00
Meta	San Juan de Arama	153,08	11,24	0,00	36,50
Meta	Uribe	17,99	0,00	0,00	0,00
Nariño	Barbacoas	39,16	3,55	34,94	83,70
Nariño	Nariño	75,67	12,39	2,05	0,00
Nariño	Contadero	187,91	13,02	1,49	1,43
Nariño	Olaya Herrera	63,08	1,61	1,28	0,00
Nariño	La Cruz	94,39	7,71	1,11	0,00
Nariño	Yacuanquer	179,19	12,95	0,97	1,83

Nariño	Cumbal	74,10	2,75	0,93	1,09
Nariño	Magüi	11,89	0,52	0,92	4,20
Nariño	Ipiales	141,33	6,07	0,34	0,23
Nariño	El Charco	27,88	1,42	0,26	0,00
Nariño	Túquerres	94,91	7,37	0,25	0,98
Nariño	San Andres de Tumaco	38,09	1,74	0,10	0,16
Nariño	Pasto	181,58	10,41	0,10	0,63
Nariño	Alban	73,57	1,93	0,00	0,00
Nariño	Aldana	229,66	11,09	0,00	1,58
Nariño	Ancuyá	221,03	24,71	0,00	0,00
Nariño	Arboleda	201,84	3,99	0,00	0,00
Nariño	Belén	65,09	8,10	0,00	0,00
Nariño	Buesaco	151,62	9,77	0,00	0,42
Nariño	Chachagüí	172,79	13,07	0,00	0,00
Nariño	Colón	157,40	1,02	0,00	11,15
Nariño	Consaca	234,28	16,83	0,00	1,05
Nariño	Córdoba	125,34	3,58	0,00	0,72
Nariño	Cuaspud	101,23	8,18	0,00	0,00
Nariño	Cumbitara	44,01	0,70	0,00	0,00
Nariño	El Peñol	166,33	7,72	0,00	1,48
Nariño	El Rosario	36,58	1,93	0,00	0,00
Nariño	El Tablón de Gómez	81,23	4,69	0,00	1,51
Nariño	El Tambo	194,20	11,79	0,00	0,00
Nariño	Francisco Pizarro	20,94	0,00	0,00	0,00
Nariño	Funes	201,11	12,19	0,00	4,62
Nariño	Guachucal	204,18	10,16	0,00	0,00
Nariño	Guaitarilla	99,13	6,53	0,00	0,00
Nariño	Gualmatán	93,88	5,22	0,00	0,00
Nariño	Iles	34,19	2,32	0,00	1,15
Nariño	Imués	132,64	3,21	0,00	0,00
Nariño	La Florida	74,27	3,15	0,00	0,00
Nariño	La Llanada	159,83	5,19	0,00	0,00
Nariño	La Tola	52,84	1,10	0,00	0,00
Nariño	La Unión	74,01	14,48	0,00	7,61
Nariño	Leiva	20,75	0,00	0,00	0,72
Nariño	Linares	139,25	21,43	0,00	1,92
Nariño	Los Andes	94,13	4,17	0,00	17,51
Nariño	Mallama	109,26	8,94	0,00	1,18
Nariño	Mosquera	30,66	1,27	0,00	0,00
Nariño	Ospina	140,62	4,60	0,00	9,23
Nariño	Policarpa	47,25	3,02	0,00	0,00

Nariño	Potosí	121,98	6,48	0,00	0,00
Nariño	Providencia	137,31	3,76	0,00	0,00
Nariño	Puerres	107,14	4,69	0,00	1,17
Nariño	Pupiales	76,82	3,12	0,00	0,52
Nariño	Ricaurte	64,48	4,35	0,00	1,05
Nariño	Roberto Payán	0,92	0,00	0,00	0,51
Nariño	Samaniego	90,77	3,82	0,00	0,00
Nariño	San Bernardo	78,64	1,69	0,00	1,04
Nariño	San Lorenzo	86,84	5,14	0,00	6,59
Nariño	San Pablo	70,33	5,11	0,00	0,56
Nariño	San Pedro de Cartago	174,52	6,70	0,00	0,00
Nariño	Sandoná	204,51	9,77	0,00	2,34
Nariño	Santa Bárbara	8,73	0,67	0,00	0,66
Nariño	Santacruz	18,45	0,00	0,00	0,34
Nariño	Sapuyes	109,82	6,22	0,00	1,60
Nariño	Taminango	112,86	6,96	0,00	0,00
Nariño	Tangua	89,41	8,30	0,00	0,00
Norte de Santander	Bochalema	192,04	28,86	2,95	0,00
Norte de Santander	San Cayetano	167,57	14,92	1,95	0,00
Norte de Santander	Villa Caro	57,96	19,24	1,95	5,80
Norte de Santander	Pamplona	209,81	17,43	1,30	0,36
Norte de Santander	Toledo	128,47	16,20	1,16	100,11
Norte de Santander	Los Patios	240,55	39,74	1,14	0,42
Norte de Santander	El Zulia	153,67	17,48	0,91	1,35
Norte de Santander	Tibú	101,15	11,02	0,85	7,98
Norte de Santander	Cúcuta	155,67	25,01	0,78	0,40
Norte de Santander	Chinácota	228,21	22,58	0,66	0,61
Norte de Santander	El Carmen	120,31	19,39	0,63	4,15
Norte de Santander	Ocaña	138,84	16,04	0,52	1,78
Norte de Santander	Sardinata	75,91	8,38	0,44	9,71
Norte de Santander	Villa del Rosario	199,56	31,57	0,27	0,34
Norte de Santander	Abrego	67,78	5,10	0,00	0,00
Norte de Santander	Arboledas	104,14	4,44	0,00	31,16
Norte de Santander	Bucarasica	89,79	0,00	0,00	0,00
Norte de Santander	Cachirá	78,77	3,65	0,00	0,00
Norte de Santander	Cácota	285,78	9,78	0,00	5,34
Norte de Santander	Chitagá	58,96	4,81	0,00	29,84
Norte de Santander	Convención	205,32	16,78	0,00	0,74
Norte de Santander	Cucutilla	131,30	6,38	0,00	2,55
Norte de Santander	Durania	269,74	25,70	0,00	15,38
Norte de Santander	El Tarra	101,57	2,74	0,00	8,25

Norte de Santander	Gramalote	44,15	3,59	0,00	1,67
Norte de Santander	Hacarí	85,96	1,94	0,00	0,00
Norte de Santander	Herrán	57,62	2,50	0,00	0,00
Norte de Santander	La Esperanza	61,30	6,72	0,00	10,94
Norte de Santander	La Playa	97,47	7,04	0,00	1,17
Norte de Santander	Labateca	140,13	8,55	0,00	18,73
Norte de Santander	Lourdes	80,00	2,96	0,00	2,95
Norte de Santander	Mutiscua	58,07	2,67	0,00	2,60
Norte de Santander	Pamplonita	108,03	12,19	0,00	0,00
Norte de Santander	Puerto Santander	74,98	6,15	0,00	0,00
Norte de Santander	Ragonvalia	61,03	10,18	0,00	14,50
Norte de Santander	Salazar	175,97	19,93	0,00	1,12
Norte de Santander	San Calixto	39,21	2,26	0,00	13,79
Norte de Santander	Santiago	135,66	10,71	0,00	25,58
Norte de Santander	Silos	81,48	4,58	0,00	0,00
Norte de Santander	Teorama	33,53	3,37	0,00	4,00
Putumayo	Leguízamo	201,92	27,71	3,20	1,27
Putumayo	Villagarzón	177,98	11,41	2,38	0,96
Putumayo	Mocoa	323,74	18,60	1,71	3,08
Putumayo	Puerto Asís	133,21	13,42	0,86	1,19
Putumayo	San Miguel	157,66	3,50	0,82	1,93
Putumayo	Valle del Guamuez	98,99	8,78	0,81	4,49
Putumayo	Sibundoy	134,70	4,98	0,74	0,00
Putumayo	Puerto Caicedo	125,97	7,63	0,69	12,35
Putumayo	Orito	152,80	9,85	0,61	9,53
Putumayo	Colón	100,61	14,65	0,00	0,00
Putumayo	Puerto Guzmán	29,89	1,73	0,00	0,00
Putumayo	San Francisco	61,39	1,40	0,00	0,00
Putumayo	Santiago	87,16	3,94	0,00	0,00
Quindio	Montenegro	239,00	39,17	1,46	0,98
Quindio	Armenia	211,13	29,19	1,05	0,48
Quindio	Quimbaya	253,27	41,64	0,86	0,29
Quindio	Circasia	316,59	40,29	0,34	2,36
Quindio	Calarca	221,99	37,06	0,13	0,65
Quindio	Buenavista	73,64	52,64	0,00	0,00
Quindio	Córdoba	33,46	24,49	0,00	0,00
Quindio	Filandia	269,95	37,44	0,00	0,76
Quindio	Génova	234,78	30,83	0,00	0,00
Quindio	La Tebaida	203,31	27,37	0,00	1,57
Quindio	Pijao	313,62	46,72	0,00	0,00
Quindio	Salento	213,17	18,24	0,00	0,00

Risaralda	Marsella	222,29	34,40	2,20	1,78
Risaralda	Belén de Umbría	242,76	34,63	1,44	0,36
Risaralda	Santuario	203,64	38,30	1,29	0,64
Risaralda	Pereira	189,81	27,01	0,98	0,44
Risaralda	Pueblo Rico	90,08	7,72	0,76	0,00
Risaralda	Santa Rosa de Cabal	164,17	22,39	0,70	0,56
Risaralda	La Virginia	276,02	36,95	0,63	0,32
Risaralda	Dosquebradas	157,49	24,78	0,63	0,16
Risaralda	Apía	179,26	21,44	0,00	0,54
Risaralda	Balboa	134,24	28,43	0,00	1,58
Risaralda	Guática	230,05	22,70	0,00	0,00
Risaralda	La Celia	143,59	22,03	0,00	0,00
Risaralda	Mistrató	61,33	7,60	0,00	0,00
Risaralda	Quinchía	181,01	15,42	0,00	1,18
Santander	Valle de San José	244,19	24,67	8,39	2,09
Santander	Sabana de Torres	281,39	35,54	7,77	4,23
Santander	Albania	31,87	3,87	5,89	0,00
Santander	Hato	203,71	21,25	4,21	0,00
Santander	Palmas del Socorro	271,32	35,23	4,20	4,49
Santander	San Joaquín	279,35	0,00	3,71	0,00
Santander	Jesús María	142,06	28,57	3,19	3,19
Santander	Barrancabermeja	166,03	39,90	2,97	1,93
Santander	Tona	70,28	5,73	2,88	1,47
Santander	Contratación	183,74	11,20	2,57	0,00
Santander	Páramo	211,62	12,24	2,41	0,00
Santander	Güepsa	392,46	33,95	2,40	0,00
Santander	Puerto Wilches	94,27	13,33	1,59	0,95
Santander	Lebríja	224,33	27,91	1,51	2,29
Santander	Bucaramanga	120,33	24,09	1,39	1,09
Santander	San Gil	206,61	24,79	1,34	0,23
Santander	Barichara	247,95	48,11	1,32	0,00
Santander	Piedecuesta	117,39	19,74	1,25	0,92
Santander	Sucre	64,30	3,53	1,19	0,00
Santander	San Vicente de Chucurí	106,15	18,59	1,16	1,17
Santander	Rionegro	152,13	15,34	1,07	4,98
Santander	Floridablanca	113,58	22,32	1,06	1,23
Santander	Cimitarra	120,28	17,36	1,00	1,28
Santander	Mogotes	193,61	26,62	0,91	0,00
Santander	El Playón	146,53	17,53	0,86	3,32
Santander	Los Santos	113,95	17,63	0,84	0,83
Santander	Girón	91,42	19,63	0,74	0,31

Santander	Barbosa	165,09	30,75	0,73	1,83
Santander	Málaga	245,15	34,70	0,54	1,63
Santander	Vélez	164,03	9,40	0,51	1,04
Santander	Socorro	204,28	31,02	0,34	0,00
Santander	Aguada	100,90	5,19	0,00	5,39
Santander	Aratoca	159,79	16,83	0,00	1,20
Santander	Betulia	123,23	15,46	0,00	13,58
Santander	Bolívar	202,32	6,33	0,00	0,00
Santander	Cabrera	286,38	8,81	0,00	0,00
Santander	California	188,64	5,11	0,00	0,00
Santander	Capitanejo	227,33	40,93	0,00	0,00
Santander	Carcasí	120,11	9,77	0,00	0,00
Santander	Cepitá	84,08	26,73	0,00	0,00
Santander	Cerrito	186,56	34,46	0,00	0,00
Santander	Charalá	106,51	11,28	0,00	0,92
Santander	Charta	144,97	14,83	0,00	0,00
Santander	Chima	114,56	12,84	0,00	0,00
Santander	Chipatá	133,31	0,00	0,00	0,00
Santander	Concepción	174,89	24,16	0,00	77,53
Santander	Confines	177,21	11,09	0,00	0,00
Santander	Coromoro	59,74	3,99	0,00	0,00
Santander	Curití	204,37	29,48	0,00	0,00
Santander	El Carmen de Chucurí	68,27	3,61	0,00	1,52
Santander	El Guacamayo	152,19	4,92	0,00	0,00
Santander	El Peñón	90,37	5,69	0,00	0,00
Santander	Encino	23,78	3,97	0,00	0,00
Santander	Enciso	321,37	141,24	0,00	3,01
Santander	Florián	123,16	9,48	0,00	0,00
Santander	Galán	109,12	8,45	0,00	4,33
Santander	Gambita	73,20	7,91	0,00	1,97
Santander	Guaca	97,17	3,07	0,00	0,00
Santander	Guadalupe	129,72	29,09	0,00	2,07
Santander	Guapotá	202,61	27,73	0,00	4,72
Santander	Guavatá	177,52	12,91	0,00	0,00
Santander	Jordán	8,90	9,10	0,00	0,00
Santander	La Belleza	105,14	8,18	0,00	0,00
Santander	La Paz	59,69	3,82	0,00	5,84
Santander	Landázuri	111,39	7,82	0,00	0,65
Santander	Macaravita	103,25	11,99	0,00	0,00
Santander	Matanza	249,81	20,29	0,00	0,00
Santander	Molagavita	106,48	1,87	0,00	0,00

Santander	Ocamonte	193,82	12,54	0,00	0,00
Santander	Oiba	228,00	32,79	0,00	0,00
Santander	Onzaga	96,09	11,77	0,00	0,00
Santander	Palmar	33,38	0,00	0,00	0,00
Santander	Pinchote	168,87	16,21	0,00	1,89
Santander	Puente Nacional	122,21	16,56	0,00	0,00
Santander	Puerto Parra	45,30	6,87	0,00	1,43
Santander	San Andrés	166,81	11,22	0,00	1,05
Santander	San Benito	52,94	10,10	0,00	2,53
Santander	San José de Miranda	171,87	29,50	0,00	2,21
Santander	San Miguel	113,73	24,79	0,00	4,20
Santander	Santa Bárbara	216,70	46,37	0,00	0,00
Santander	Santa Helena del Opón	22,83	2,30	0,00	0,00
Santander	Simacota	178,06	19,91	0,00	1,25
Santander	Suaita	112,79	29,94	0,00	1,91
Santander	Suratá	83,51	14,77	0,00	0,00
Santander	Vetas	29,15	4,17	0,00	0,00
Santander	Villanueva	121,13	51,87	0,00	1,59
Santander	Zapatoca	110,05	20,04	0,00	0,00
Sucre	Coloso	191,68	16,83	15,39	41,63
Sucre	Tolú Viejo	205,22	3,70	3,17	39,14
Sucre	Coveñas	338,09	23,50	3,17	4,57
Sucre	Ovejas	100,01	9,45	2,35	20,21
Sucre	Palmito	101,17	4,56	2,29	10,92
Sucre	El Roble	96,09	8,64	2,05	2,94
Sucre	Morroa	236,31	22,72	1,45	7,08
Sucre	San Pedro	246,11	6,19	1,25	4,33
Sucre	San Marcos	147,46	13,85	1,15	1,82
Sucre	Buenavista	215,20	4,21	1,04	4,23
Sucre	Sincelejo	167,32	14,40	1,04	2,45
Sucre	Caimito	92,64	4,26	0,83	1,70
Sucre	Corozal	105,92	9,07	0,82	0,81
Sucre	Majagual	63,86	3,36	0,62	1,52
Sucre	Santiago de Tolú	201,57	13,99	0,61	6,67
Sucre	Galeras	139,51	7,08	0,51	2,04
Sucre	San Luis de Sincé	60,64	3,96	0,30	1,54
Sucre	Sampués	191,92	7,65	0,26	5,55
Sucre	San Onofre	94,10	3,63	0,21	13,45
Sucre	Chalán	150,51	6,91	0,00	23,10
Sucre	Guaranda	62,58	1,75	0,00	0,00
Sucre	La Unión	289,38	3,63	0,00	1,78

Sucre	Los Palmitos	43,61	2,60	0,00	3,63
Sucre	San Benito Abad	43,57	1,20	0,00	0,40
Sucre	San Juan de Betulia	124,55	4,00	0,00	0,00
Sucre	Sucre	269,78	2,23	0,00	0,00
Tolima	Ambalema	313,08	78,37	4,26	1,38
Tolima	Armero	374,32	64,93	4,05	0,79
Tolima	Valle de San Juan	166,08	11,06	3,16	0,00
Tolima	Saldaña	312,02	32,21	2,69	2,06
Tolima	Carmen de Apicalá	498,83	62,02	2,29	0,00
Tolima	Suárez	218,10	19,80	2,20	0,00
Tolima	Piedras	132,33	14,33	1,77	1,79
Tolima	Natagaima	177,10	14,07	1,75	1,32
Tolima	Honda	186,98	41,20	1,56	0,75
Tolima	Venadillo	124,23	12,88	1,54	0,00
Tolima	Melgar	384,65	75,50	1,40	1,12
Tolima	Purificación	241,86	23,44	1,39	1,75
Tolima	Flandes	227,21	44,21	1,38	0,00
Tolima	Prado	251,31	20,44	1,28	20,51
Tolima	Alvarado	223,47	33,84	1,12	2,26
Tolima	Falan	115,96	16,26	1,08	0,00
Tolima	Chaparral	177,88	14,66	1,06	3,19
Tolima	Coello	166,10	18,81	1,04	3,08
Tolima	Fresno	208,01	31,17	0,97	0,33
Tolima	Villahermosa	126,62	14,76	0,94	0,00
Tolima	Ibagué	144,62	19,01	0,92	0,74
Tolima	Rioblanco	86,25	3,64	0,81	0,00
Tolima	Espinal	303,12	43,52	0,79	0,66
Tolima	Guamo	267,70	25,88	0,60	1,51
Tolima	Mariquita	287,27	58,05	0,60	4,51
Tolima	San Luis	59,26	25,54	0,52	1,56
Tolima	Cajamarca	193,05	18,80	0,51	0,51
Tolima	Planadas	184,80	15,44	0,34	1,01
Tolima	Ortega	129,97	6,11	0,31	1,22
Tolima	Líbano	166,34	13,22	0,25	0,49
Tolima	Alpujarra	138,76	13,91	0,00	3,97
Tolima	Anzoátegui	86,59	12,38	0,00	0,00
Tolima	Ataco	63,92	3,57	0,00	0,90
Tolima	Casabianca	115,98	16,36	0,00	1,50
Tolima	Coyaima	93,91	5,31	0,00	0,71
Tolima	Cunday	76,12	13,03	0,00	0,98
Tolima	Dolores	164,23	18,17	0,00	1,25

Tolima	Herveo	115,01	10,86	0,00	1,23
Tolima	Icononzo	153,17	21,68	0,00	0,00
Tolima	Lérida	305,49	37,17	0,00	0,57
Tolima	Murillo	67,19	1,98	0,00	0,00
Tolima	Palocabildo	163,81	10,78	0,00	1,08
Tolima	Roncesvalles	55,34	9,50	0,00	1,58
Tolima	Rovira	208,99	27,43	0,00	0,96
Tolima	San Antonio	178,82	20,51	0,00	0,00
Tolima	Santa Isabel	32,61	0,00	0,00	0,00
Tolima	Villarrica	111,50	9,03	0,00	0,00
Valle del Cauca	Yotoco	208,14	34,69	3,09	1,86
Valle del Cauca	Ginebra	211,70	50,77	2,91	0,50
Valle del Cauca	Roldanillo	301,41	105,63	2,68	1,78
Valle del Cauca	La Cumbre	501,46	43,79	2,65	2,63
Valle del Cauca	Alcalá	309,25	46,21	2,55	0,49
Valle del Cauca	Bolívar	122,33	42,51	2,14	2,85
Valle del Cauca	Ansermanuevo	227,26	42,84	1,99	2,54
Valle del Cauca	La Unión	174,82	43,04	1,97	1,37
Valle del Cauca	Bugalagrande	266,22	40,98	1,41	1,40
Valle del Cauca	Candelaria	108,27	15,90	1,35	0,52
Valle del Cauca	Jamundí	238,69	34,57	1,34	1,79
Valle del Cauca	Cartago	188,07	41,50	1,32	1,40
Valle del Cauca	Calima	342,21	55,41	1,28	1,28
Valle del Cauca	Toro	146,04	37,46	1,25	1,25
Valle del Cauca	Guacarí	196,55	28,15	1,20	0,87
Valle del Cauca	Guadalajara de Buga	181,03	31,66	1,12	1,04
Valle del Cauca	Dagua	386,39	42,38	1,11	1,37
Valle del Cauca	Trujillo	176,56	38,44	1,09	7,67
Valle del Cauca	El Águila	148,17	25,51	0,92	0,92
Valle del Cauca	Zarzal	213,55	48,63	0,92	1,11
Valle del Cauca	Sevilla	211,40	30,72	0,86	0,22
Valle del Cauca	La Victoria	209,74	29,62	0,74	5,95
Valle del Cauca	Buenaventura	17,23	1,60	0,72	0,70
Valle del Cauca	Obando	286,77	67,63	0,66	3,36
Valle del Cauca	Palmira	178,27	23,53	0,60	0,54
Valle del Cauca	Andalucía	264,95	34,69	0,56	1,67
Valle del Cauca	Tuluá	103,21	22,59	0,56	0,60
Valle del Cauca	San Pedro	49,85	27,12	0,55	0,00
Valle del Cauca	Cali	119,72	21,18	0,55	0,47
Valle del Cauca	Yumbo	272,61	41,32	0,55	1,40
Valle del Cauca	El Cerrito	114,55	11,74	0,52	0,53

Valle del Cauca	Caicedonia	379,11	49,18	0,34	2,32
Valle del Cauca	Pradera	313,40	32,89	0,19	1,51
Valle del Cauca	Argelia	102,82	32,31	0,00	3,02
Valle del Cauca	El Cairo	183,12	19,37	0,00	0,00
Valle del Cauca	El Dovio	219,35	44,69	0,00	4,43
Valle del Cauca	Florida	225,35	23,59	0,00	6,32
Valle del Cauca	Restrepo	340,89	34,70	0,00	0,00
Valle del Cauca	Riofrío	265,20	47,56	0,00	0,63
Valle del Cauca	Ulloa	393,94	39,96	0,00	10,70
Valle del Cauca	Versalles	236,28	44,35	0,00	0,00
Valle del Cauca	Vijes	194,76	31,31	0,00	0,00
Vaupés	Taraira	336,93	0,00	1100,55	0,00
Vaupés	Yavaraté	32,03	0,00	391,17	39,67
Vaupés	Pacoa	22,60	0,00	201,16	142,84
Vaupés	Mitú	99,10	5,46	39,46	5,68
Vaupés	Papunahua	81,73	0,00	11,61	0,00
Vaupés	Caruru	15,06	6,02	0,00	0,00
Vichada	Puerto Carreño	288,76	43,08	5,71	26,45
Vichada	La Primavera	94,33	9,54	3,12	11,50
Vichada	Santa Rosalía	131,09	22,70	2,63	4,99
Vichada	Cumaribo	15,43	1,45	0,55	1,43
