# Supplementary material for: Epidemiological scenarios for human rabies exposure notified in Colombia during ten years: A challenge to implement surveillance actions with a differential approach on vulnerable populations
Source: PLoS One. 2019 Dec 27;14(12):e0213120. doi: 10.1371/journal.pone.0213120 (PMC6934280; doi:10.1371/journal.pone.0213120)
Supplement: S1 Appendix — (PDF) [file pone.0213120.s003.pdf]

Spatial Autocorrelation Report-Incidence HRE due Dog

Moran's Index: 0,108848
z-score: 58,451458
p-value: 0,000000

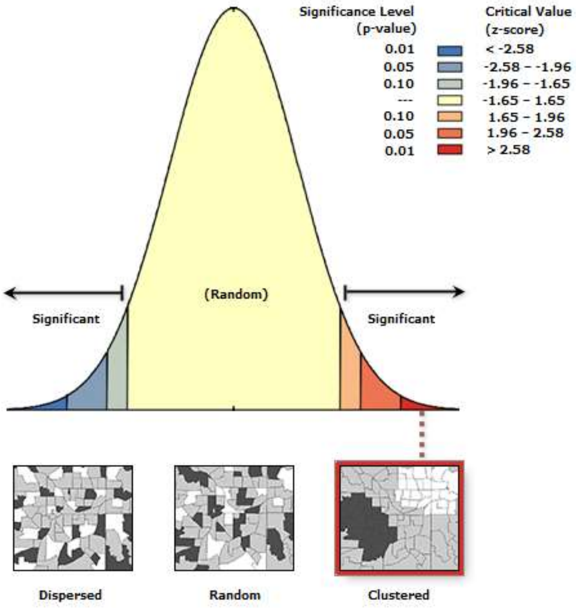

Given the z-score of 69.3591419716, there is a less than 1% likelihood that this clustered pattern could be the result of random chance.

Global Moran's I Summary

**Moran's Index:**	0,108848
**Expected Index:**	-0,000892
**Variance:**	0,000004
**z-score:**	58,451458
**p-value:**	0,000000

**Input Feature Class:**	Colombia_Incidences_Cities

Dataset Information

**Input Field:**	DOG_INCIDENCE
**Conceptualization:**	INVERSE_DISTANCE
**Distance Method:**	EUCLIDEAN
**Row Standardization:**	False
**Distance Threshold:**	681461,0293 Meters
**Weights Matrix File:**	None
**Selection Set:**	False

Spatial Autocorrelation Report-Incidence HRE due Cat

Moran's Index: 0,128900
z-score: 69,359142
p-value: 0,000000

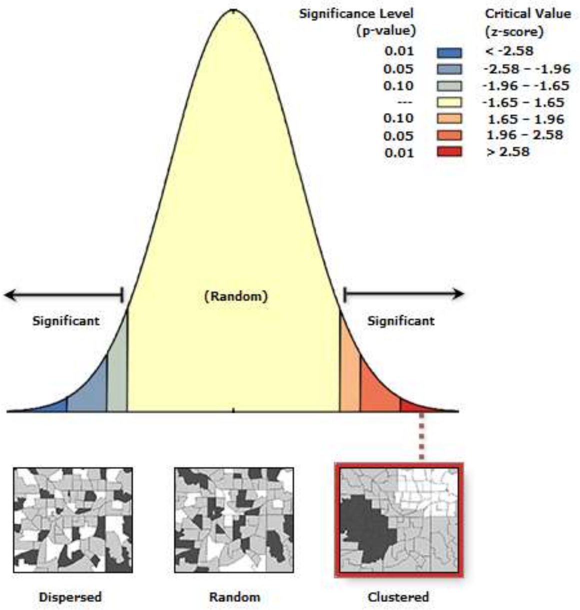

Given the z-score of 69.3591419716, there is a less than 1% likelihood that this clustered pattern could be the result of random chance.

Global Moran's I Summary

**Moran's Index:**	0,128900
**Expected Index:**	-0,000892
**Variance:**	0,000004
**z-score:**	69,359142
**p-value:**	0,000000

**Input Feature Class:**	Colombia_Incidences_Cities

Dataset Information

**Input Field:**	CAT_INCIDENCE
**Conceptualization:**	INVERSE_DISTANCE
**Distance Method:**	EUCLIDEAN
**Row Standardization:**	False
**Distance Threshold:**	681461,0293 Meters
**Weights Matrix File:**	None
**Selection Set:**	False

Spatial Autocorrelation Report-Incidence HRE due Bat

Moran's Index: 0,003397
z-score: 3,967254
p-value: 0,000073

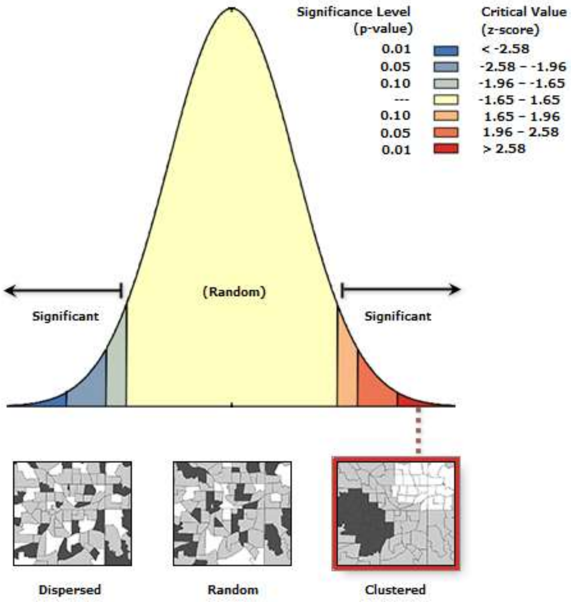

Given the z-score of 3.96725446905, there is a less than 1% likelihood that this clustered pattern could be the result of random chance.

Global Moran's I Summary

**Moran's Index:**	0,003397
**Expected Index:**	-0,000892
**Variance:**	0,000001
**z-score:**	3,967254
**p-value:**	0,000073

Dataset Information

**Input Feature Class:**	Colombia_Incidences_Cities
**Input Field:**	BAT
**Conceptualization:**	INVERSE_DISTANCE
**Distance Method:**	EUCLIDEAN
**Row Standardization:**	False
**Distance Threshold:**	681461,0293 Meters
**Weights Matrix File:**	None
**Selection Set:**	False

Spatial Autocorrelation Report-Incidence HRE due Farm Animal

Moran's Index: 0,023751
z-score: 13,468863
p-value: 0,000000

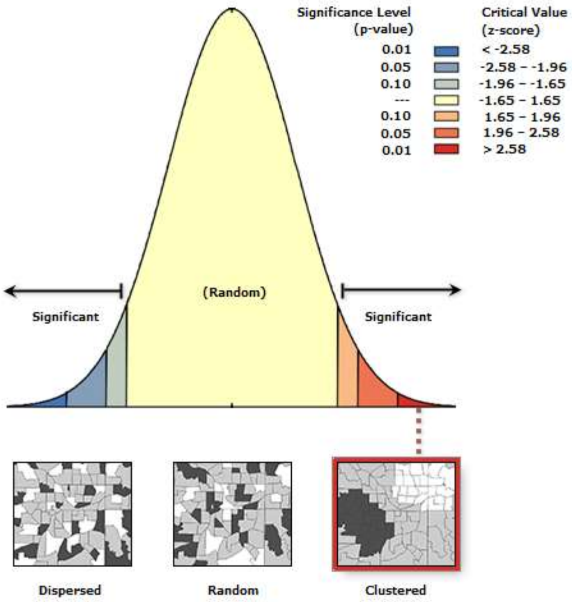

Given the z-score of 13.4688634282, there is a less than 1% likelihood that this clustered pattern could be the result of random chance.

Global Moran's I Summary

**Moran's Index:**	0,023751
**Expected Index:**	-0,000892
**Variance:**	0,000003
**z-score:**	13,468863
**p-value:**	0,000000

Dataset Information

**Input Feature Class:**	Colombia_Incidences_Cities
**Input Field:**	FARM_ANIMALS
**Conceptualization:**	INVERSE_DISTANCE
**Distance Method:**	EUCLIDEAN
**Row Standardization:**	False
**Distance Threshold:**	681461,0293 Meters
**Weights Matrix File:**	None
**Selection Set:**	False
